# Sex‐Specific Reproductive Factors Augment Cardiovascular Disease Risk in Women: A Mendelian Randomization Study

**DOI:** 10.1161/JAHA.122.027933

**Published:** 2023-02-27

**Authors:** Maddalena Ardissino, Eric A. W. Slob, Paul Carter, Tormod Rogne, Joanna Girling, Stephen Burgess, Fu Siong Ng

**Affiliations:** ^1^ National Heart and Lung Institute Imperial College London London United Kingdom; ^2^ Nuffield Department of Population Health University of Oxford Oxford United Kingdom; ^3^ Medical Research Council Biostatistics Unit University of Cambridge Cambridge United Kingdom; ^4^ Department of Applied Economics, Erasmus School of Economics Erasmus University Rotterdam Rotterdam The Netherlands; ^5^ Erasmus University Rotterdam Institute for Behavior and Biology, Erasmus University Rotterdam Rotterdam The Netherlands; ^6^ Department of Medicine University of Cambridge Cambridge United Kingdom; ^7^ Department of Chronic Disease Epidemiology Yale School of Public Health New Haven CT; ^8^ Department of Circulation and Medical Imaging Norwegian University of Science and Technology Trondheim Norway; ^9^ Centre for Fertility and Health Norwegian Institute of Public Health Oslo Norway; ^10^ Department of Obstetrics and Gynaecology Chelsea and Westminster Hospital NHS Foundation Trust London United Kingdom; ^11^ Cardiovascular Epidemiology Unit, Department of Public Health and Primary Care University of Cambridge Cambridge United Kingdom

**Keywords:** age at first birth, cardiovascular disease, menarche, menopause, parity, reproductive, Cardiovascular Disease, Epidemiology, Pregnancy, Risk Factors, Women

## Abstract

**Background:**

Observational studies suggest that reproductive factors are associated with cardiovascular disease, but these are liable to influence by residual confounding. This study explores the causal relevance of reproductive factors on cardiovascular disease in women using Mendelian randomization.

**Methods and Results:**

Uncorrelated (*r* ^2^<0.001), genome‐wide significant (*P*<5×10^−8^) single‐nucleotide polymorphisms were extracted from sex‐specific genome‐wide association studies of age at first birth, number of live births, age at menarche, and age at menopause. Inverse‐variance weighted Mendelian randomization was used for primary analyses on outcomes of atrial fibrillation, coronary artery disease, heart failure, ischemic stroke, and stroke. Earlier genetically predicted age at first birth increased risk of coronary artery disease (odds ratio [OR] per year, 1.49 [95% CI, 1.28–1.74], *P*=3.72×10^−7^) heart failure (OR, 1.27 [95% CI, 1.06–1.53], *P*=0.009), and stroke (OR, 1.25 [95% CI, 1.00–1.56], *P*=0.048), with partial mediation through body mass index, type 2 diabetes, blood pressure, and cholesterol traits. Higher genetically predicted number of live births increased risk of atrial fibrillation (OR for <2, versus 2, versus >2 live births, 2.91 [95% CI, 1.16–7.29], *P*=0.023), heart failure (OR, 1.90 [95% CI, 1.28–2.82], *P*=0.001), ischemic stroke (OR, 1.86 [95% CI, 1.03–3.37], *P*=0.039), and stroke (OR, 2.07 [95% CI, 1.22–3.52], *P*=0.007). Earlier genetically predicted age at menarche increased risk of coronary artery disease (OR per year, 1.10 [95% CI, 1.06–1.14], *P*=1.68×10^−6^) and heart failure (OR, 1.12 [95% CI, 1.07–1.17], *P*=5.06×10^−7^); both associations were at least partly mediated by body mass index.

**Conclusions:**

These results support a causal role of a number of reproductive factors on cardiovascular disease in women and identify multiple modifiable mediators amenable to clinical intervention.

**Nonstandard Abbreviations and Acronyms**
EA: educational attainment
MR: Mendelian randomization
SBP: systolic blood pressure
T2D: type 2 diabetes

**Clinical Perspective**

**What Is New?**

- This study provides genetic evidence to support that earlier first birth, higher number of live births, and earlier menarche are associated with higher risk of atrial fibrillation, coronary artery disease, heart failure, and stroke in women.
- For age at first birth, this increased risk was at least partly mediated by traditional cardiometabolic risk factors: body mass index, high‐density lipoprotein cholesterol, and systolic blood pressure.
- For age at menarche, this increased risk was largely mediated by higher body mass index.

**What Are the Clinical Implications?**

- The results support the emerging research focus on female‐specific risk factors, stressing the importance of their routine evaluation in clinical risk stratification.
- Additionally, the results highlight that close monitoring and early modification of cardiometabolic factors is a key strategy that will at least partly mitigate the increased cardiovascular risk conferred by these reproductive factors.

Cardiovascular disease (CVD) is a leading cause of morbidity and mortality in women. In the general population, a large proportion of the burden of CVD can be explained by well‐established “traditional” risk factors that include family history, hypertension, diabetes, obesity, smoking, hypercholesterolemia, and male sex.^1^ Importantly though, women with cardiovascular events tend to have different clinical presentations than men, are more often mischaracterized as low risk^2^ and ultimately have been reported to have a worse prognosis.^3^, ^4^ Sex‐specific factors might thus improve prediction of cardiovascular risk in women.

In recent years, observational research has identified that reproductive factors such as early menarche, early menopause, recurrent pregnancy loss, and the timing and number of births are all associated with later life CVD in women,^5^, ^6^, ^7^, ^8^, ^9^, ^10^, ^11^, ^12^, ^13^, ^14^ with important implications for CVD prevention and risk profiling. However, such observational studies are limited by potential bias from residual confounding. This limits causal inference relating to the role of reproductive factors on CVD, and their causal role above and beyond that of other “traditional” cardiovascular risk factors. Indeed, many reproductive factors that are associated with CVD, such as higher parity, are also associated with adverse cardiovascular risk factor profiles^15^, ^16^, ^17^ and differences in socioeconomic and behavioral factors,^17^ providing viable pathways for confounding. The potential influence of these time‐varying socioeconomic confounders is difficult to account for using observational data, owing to limitations in the ability to optimally measure and adjust for them.

The Mendelian randomization (MR) framework can be used to provide more reliable estimates of the causal effects of risk factors on outcomes in this setting. Because the process of random allele assortment at conception leads to an effective “randomization” of individuals to high or low genetic risk of diseases or phenotypes, the genetic liability for a risk factor (eg, age at menarche) can be used as a proxy indicator for the exposure. Because the allocation to “high” or “low” genetic risk is random and therefore not influenced by confounding or reverse causation, this framework can be used to infer causality of the risk factor on an outcome under a set of key assumptions.

The aim of this study was to use the MR framework to explore the causal pathways underlying the associations between female reproductive history (age at first birth, number of live births, age at menarche, age at menopause) and risk of multiple CVDs (atrial fibrillation, coronary artery disease, heart failure, ischemic stroke, and stroke). For any associations discovered, potential mediating pathways through traditional, modifiable cardiovascular risk factors of body mass index (BMI), type 2 diabetes (T2D), systolic blood pressure (SBP), high‐density lipoprotein cholesterol (HDL), and low‐density lipoprotein cholesterol were also explored. Finally, based on prior evidence of a genetic correlation between reproductive traits and educational attainment (EA),^18^ we aimed to assess whether accounting for EA, an important measure of social, behavioral, and economic domains, might explain part of any putative associations between reproductive factors and CVD.

## METHODS

### Ethics and Data Access

Publicly available genome‐wide association summary data were used for all primary analyses. All data and materials for these are publicly available at cited sources. Ethical approval and participant consent were obtained in each of the original studies that generated the data. Replication analysis on UK Biobank data was performed under application number 48666, covered by the general ethical approval for UK Biobank studies from the National Health Service National Research Ethics. Because of the sensitive individual‐level nature of these data, they are not available to share by the authors but can be accessed by application directly to the UK Biobank. The paper is reported on the basis of recommendations by the Strengthening the Reporting of Observational Studies in Epidemiology Using Mendelian Randomization Guidelines.^19^ All statistical analyses were performed using R version 4.1.1 (2021‐02‐15)^20^ using the TwoSampleMR^21^ and Mendelian Randomization packages.^22^

### Instrumental Variable Selection

Instrumental variables were extracted from summary statistics of published sex‐specific studies on the exposures of interest: self‐reported age at first birth (n=131 987 parous women, unit = years) and number of live births (n=193 953 parous women, number of live births coded into 3 categories of <2, 2, or >2 live births) from Neale laboratory's second release analysis of UK Biobank data (http://www.nealelab.is/uk‐biobank/), age at menarche (n=329 345, unit = years) from the genome‐wide association study (GWAS) on European ancestry participants the ReproGen consortium,^23^ and age at menopause (n=106 048, unit=years) from the GWAS of Ruth et al in participants of European ancestry.^24^ Further details on study cohorts are provided in Table 1. Instrumental variable single‐nucleotide polymorphisms (SNPs) were selected if they were associated with the exposure of interest in the respective GWAS at genome‐wide significance (*P*<5×10^−8^). After harmonization with the outcome data, which was performed using the “*harmonise_data*” function in the TwoSampleMR package (with attempt to infer positive strand alleles using allele frequencies for palindromes), SNPs were clumped to retain only uncorrelated variants (pair‐wise linkage disequilibrium *r* ^2^<0.001). Instrument strength was quantified using *F*‐statistics. *F*‐statistic for univariable analyses was calculated using the formula

$$F=\frac{\left(n-k-1\right)}{k}\;\frac{\left(R^{2}\right)}{\left(1-R^{2}\right)}$$

where $R^{2}$ is the explained variance in the regression of all SNPs, *n* is the number of participants in the study, and *k* is the number of instrumental variants. The $R^{2}$ was calculated as the sum of SNP‐wise $R^{2}$ of instruments, which is obtained with the formula

**Table 1:** Information on the Studies and Consortia From Which Genetic Association Data Were Obtained

Phenotype	Study or consortium	Ancestry	Sex	Cases (/controls)	Units	Link
Exposures						
Age at first birth	Neale laboratory	EUR	Female only	131 987	Year	http://www.nealelab.is/uk‐biobank/
Number of live births	Neale laboratory	EUR	Female only	193 953	Categorical number of live births (<2, 2, >2)	http://www.nealelab.is/uk‐biobank/
Age at menarche	Day et al	EUR	Female only	329 345	Year	https://doi.org/10.1038/ng.3841
Age at menopause	Ruth et al	EUR	Female only	106 048	Year	https://doi.org/10.1038/s41586‐021‐03779‐7
Additional exposure for multivariable analysis						
Educational attainment	Lee et al	EUR	Both (sex‐adjusted)	257 841	Years of education	https://doi.org/10.1038/s41588‐018‐0147‐3
Outcomes						
Coronary artery disease	Van der Harst et al	EUR	Both (sex‐adjusted)	122 733/424528	Log(OR) for coronary artery disease	https://doi.org/10.1161/CIRCRESAHA.117.312086
Stroke	Malik et al	EUR	Both (sex‐adjusted)	40 585/406111	Log(OR) for any stroke	https://doi.org/10.1038/s41588‐018‐0058‐3
Ischemic stroke	Malik et al	EUR	Both (sex‐adjusted)	34 217/406111	Log(OR) for ischemic stroke	https://doi.org/10.1038/s41588‐018‐0058‐3
Heart failure	Shah et al	EUR	Both (sex‐adjusted)	47 309/930014	Log(OR) for heart failure	https://doi.org/10.1038/s41467‐019‐13690‐5
Atrial fibrillation	Nielsen et al	EUR	Both (sex‐adjusted)	60 620/970216	Log(OR) for atrial fibrillation	https://doi.org/10.1038/s41588‐018‐0171‐3
Mediators						
Body mass index	Pulit et al	EUR	Both (sex‐adjusted)	434 794	1‐SD body mass index	https://doi.org/10.1093/hmg/ddy327
High‐density lipoprotein cholesterol	Willer et al	EUR	Both (sex‐adjusted)	187 167	1‐SD high‐density lipoprotein cholesterol	https://doi.org/10.1038/ng.2797
Low‐density lipoprotein cholesterol	Willer et al	EUR	Both (sex‐adjusted)	187 167	1‐SD low‐density lipoprotein cholesterol	https://doi.org/10.1038/ng.2797
Type 2 diabetes	Xue et al	EUR	Both (sex‐adjusted)	80 154 /596424	Log(OR) type 2 diabetes	https://doi.org/10.1038/s41467‐018‐04951‐w
Systolic blood pressure	Evangelou et al	EUR	Both (sex‐adjusted)	757 601	1‐mm Hg systolic blood pressure	https://doi.org/10.1038/s41588‐018‐0205‐x
Replication						
Coronary artery disease	UK Biobank	EUR	Female only	11 802/198 815	Log(OR) for coronary artery disease	
Stroke	UK Biobank	EUR	Female only	5411/198 815	Log(OR) for any stroke	
Ischemic stroke	UK Biobank	EUR	Female only	2777/198 815	Log(OR) for ischemic stroke	
Heart failure	UK Biobank	EUR	Female only	4128/198 815	Log(OR) for heart failure	
Atrial fibrillation	UK Biobank	EUR	Female only	9420/198 815	Log(OR) for atrial fibrillation	

EUR indicates European; and OR, odds ratio.

$R^{2}=\frac{F}{\left(n-2+F\right)}$ with $F={\left(\frac{\beta }{\mathrm{SE}\left(\beta \right)}\right)}^{2}$

where β represents the effect size of the genetic variant in the exposure GWAS, and SE(β) represents the standard error of the effect size of the genetic variant in the exposure GWAS. For multivariable analyses, instrument strength was assessed using conditional F‐statistics calculated using the MVMR package.^25^, ^26^

### Study Outcomes

Genetic association estimates for the outcomes were extracted from publicly available GWAS summary statistics on atrial fibrillation (60 620 cases and 970 216 controls),^27^ coronary artery disease (122 733 cases and 424 528 controls),^28^ heart failure (47 309 cases and 930 014 controls),^29^ ischemic stroke (34 217 cases and 406 111 controls),^30^ and stroke of any type (40 585 cases and 406 111 controls).^30^ All GWASs were on populations of predominantly European ancestry. Further details on study cohorts are provided in Table 1 and Table S1.

### Statistical Analysis

**Figure 1:**
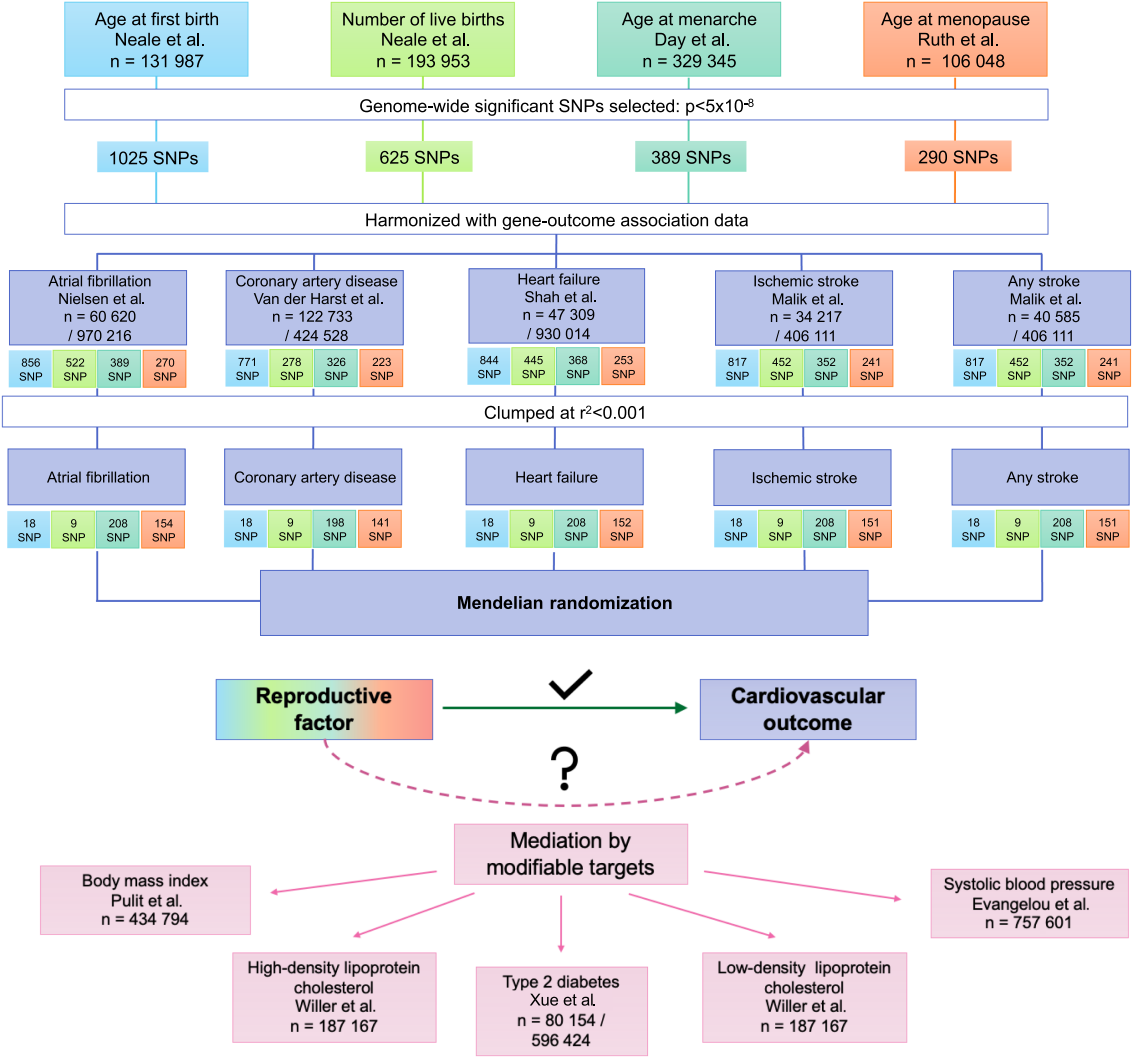
Flow chart of study methodology. SNP indicates single‐nucleotide polymorphism.

The flow chart for the study methods is displayed in Figure 1. The data sources for gene‐exposure and gene‐outcome associations and methods for primary analysis were established by authors before the commencement of analysis. Inverse‐variance weighted (IVW) MR with multiplicative random effects^31^ was used as the primary analysis method for all models, to estimate the association between each genetically predicted reproductive factor and each cardiovascular outcome.^32^ Results are presented as odds ratios (ORs) with respective 95% CIs for each genetically predicted reproductive factor (exposure) and CVD (outcome) pair. Statistical significance for the primary analyses was considered at a value of *P*<0.0125, based on 4 independent hypotheses tested for each outcome.

### Sensitivity Analyses

The first sensitivity analysis was carried out using weighted median MR, MR‐Egger, and MR‐PRESSO.^33^ The validity of the results of the primary IVW analysis rely on each instrumental variable satisfying a set of 3 core assumptions:

1. The instrumental variant must be associated with the exposure.
2. The instrumental variant must not be associated with confounders of the association between the exposure and the outcome.
3. The instrumental variant must exert effects on the outcome only through the exposure, and not directly or through alternative (horizontally pleiotropic) pathways.

In situations where genetic variants act through additional parallel biological pathways, these assumptions are violated. This is termed horizontal pleiotropy. Sensitivity analysis using weighted median MR,^34^ MR‐PRESSO, and MR‐Egger were performed to explore this. The weighted median method can provide consistent estimates assuming at least half the weight is derived from valid SNPs.^34^ The MR‐Egger method can be used to identify the presence of directional pleiotropy under a weaker assumption that the instrument strength is independent of direct effects (InSIDE assumption).^35^ Additionally, the MR‐PRESSO analysis aims to detect SNPs with outlier effects and provides an estimate of the causal effect after adjusting for the outlier effects. Finally, the full list of SNPs used for each exposure was queried in PhenoScanner,^36^, ^37^ to investigate the presence of association with additional phenotypes in published GWASs.

The second sensitivity analysis involved performing bidirectional MR analyses. Though CVD does not tend to occur during reproductive years, some individuals at extremely high risk develop CVD before the end of the reproductive timespan, and this may feasibly affect future reproductive choices because it makes pregnancy higher risk. This implies that a bidirectional association might exist. In order to explore potential bias stemming from this, bidirectional MR was carried out for exposure‐outcome pairs significant on primary analysis. This entailed reversing the direction of analysis for exposure‐outcome pairs, thereby assessing the impact of cardiovascular events on reproductive factors. Where the outcomes in these analyses are continuous, results are presented as beta coefficients (*b*) with respective SE.

The third sensitivity analysis involved accounting for potential shared genetic effects with sociobehavioral traits. Age at first birth and number of live births have previously been reported to be highly genetically correlated with multiple sociobehavioral traits^18^ that are also known causes of CVD. In light of this, we set out to explore whether accounting for EA, an important measure of social, behavioral, and economic domains, might explain part of any putative associations between reproductive factors and CVD. Multivariable MR was thus used to estimate the impact of each reproductive factor on CVD after accounting for EA (measured as number of years of schooling completed, n=257 841 individuals),^38^ for the exposure‐outcome pairs significant on primary analysis.

The final sensitivity analysis involved replication of the analyses on female‐only outcome data. For this analysis, SNP‐outcome genetic associations were calculated in the female participants of the UK Biobank. Details on genotyping, outcome definition, and protocol of the UK Biobank have been reported previously.^39^

### Mediation Analyses

Where an association was discovered between a reproductive factor and an outcome, mediation analysis was carried out using multivariable MR to explore potential mediating pathways amenable to intervention.^40^ The putative mediators considered include BMI^41^ (n=434 794, European ancestry), HDL^42^ (n=187 167, European ancestry), low‐density lipoprotein cholesterol^42^ (n=187 167, European ancestry), T2D^43^ (n=80 154 cases and n=596 424 controls, European ancestry), and SBP^44^ (n=757 601, European ancestry).

Mediation analysis was carried out using a step‐wise approach. First, exposure‐outcome associations that were nominally significant (*P* < 0.05) on primary analysis with IVW MR were identified. Effect estimates from these analyses are considered the ‘total effect’ of the exposure on the outcome. Second, for each of these exposure‐outcome associations, putative exposure‐mediator associations were tested for each exposure‐mediator pair, using univariable IVW MR. Only pathways where both analyses produced nominally significant results (*P* < 0.05) were carried forward, under the implicit assumption of no reverse causation between the mediator and the outcome. Third, multivariable MR was carried out to estimate the effect of the exposure on the outcome that is conditional on the mediator (“direct” effect, reported as an adjusted OR with respective 95% CI). This was done by extracting genome‐wide significant (*P*<5×10^−8^) variants associated with either the exposure or the mediator, harmonization of these variants with outcome data, subsequent clumping, and multivariable IVW analysis. Because the study outcomes are binary and not rare, the “indirect” effect and proportion mediated were not calculated, as this calculation relies on linearity of relationships that cannot be assumed when using OR effect measures for a common outcome. The “direct” effect was thus qualitatively compared with the “total” effect, where substantial attenuation of the effect estimates after conditioning by the mediator is taken to suggest the presence of a mediating pathway.^40^

## RESULTS

### Age at First Birth

**Figure 2:**
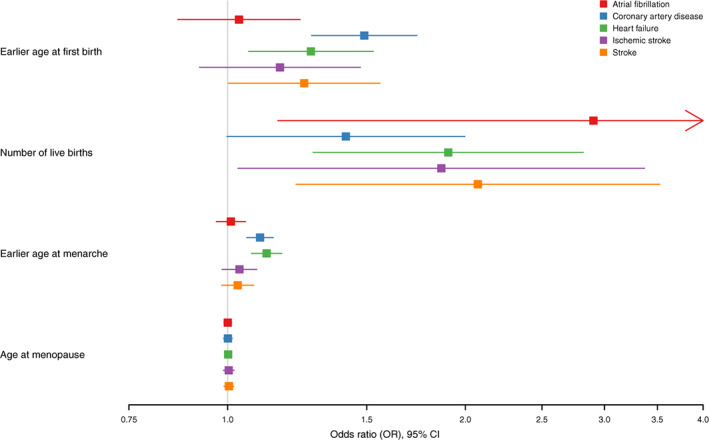
Mendelian randomization estimates for the effects of age at first birth, number of live births, age at menarche, and age at menopause on cardiovascular outcomes. OR indicates odds ratio.

Earlier genetically predicted age at first birth was associated with increased risk of coronary artery disease (OR per 1‐year earlier age at first birth, 1.49 [95% CI, 1.28–1.74], *P*=3.72×10^−7^), increased risk of heart failure (OR, 1.27 [95% CI, 1.06–1.53], *P*=0.009), and increased risk of stroke (OR, 1.25 [95% CI, 1.00–1.56], *P*=0.048) at nominal significance. There was no significant association between genetically predicted age at first birth and atrial fibrillation (OR, 1.03 [95 %CI, 0.86–1.24], *P*=0.716) or ischemic stroke (OR, 1.16 [95% CI, 0.92–1.47], *P*=0.202). The results are summarized in Table 2 and Figure 2.

**Table 2:** Mendelian Randomization Estimates for the Effects of Reproductive Factors on Cardiovascular Outcomes, Using an Inverse Variance Weighted Model With Multiplicative Random Effects, or Wald Ratio Method in Cases Where Only 1 Instrument Was Present

Exposure	Outcome	#SNP	Odds ratio	Lower 95% CI	Upper 95% CI	*P* value
Age at first birth (per 1‐y reduction)	Atrial fibrillation	18	1.03	0.86	1.24	0.716
	Coronary artery disease	18	1.49	1.28	1.74	3.72×10^−7^
	Heart failure	18	1.27	1.06	1.53	0.009
	Ischemic stroke	18	1.16	0.92	1.47	0.202
	Stroke	18	1.25	1.00	1.56	0.048
Number of live births (per increase in category across <2, vs 2, vs >2 live births)	Atrial fibrillation	9	2.91	1.16	7.29	0.023
	Coronary artery disease	9	1.41	1.00	2.00	0.051
	Heart failure	9	1.90	1.28	2.82	0.001
	Ischemic stroke	9	1.86	1.03	3.37	0.039
	Stroke	9	2.07	1.22	3.52	0.007
Age at menarche (per 1‐y reduction)	Atrial fibrillation	208	1.01	0.97	1.05	0.664
	Coronary artery disease	198	1.10	1.06	1.14	1.68×10^−6^
	Heart failure	208	1.12	1.07	1.17	5.06×10^−7^
	Ischemic stroke	208	1.04	0.98	1.09	0.182
	Stroke	208	1.03	0.98	1.08	0.222
Age at menopause (per 1‐y increase)	Atrial fibrillation	154	1.00	0.99	1.01	0.940
	Coronary artery disease	141	1.00	0.99	1.01	0.894
	Heart failure	152	1.00	0.99	1.01	0.735
	Ischemic stroke	151	1.00	0.98	1.01	0.693
	Stroke	151	1.00	0.98	1.01	0.606

#SNP indicates number of SNPs used in analysis; and SNP, single‐nucleotide polymorphism.

Sensitivity MR analyses were not suggestive of pleiotropy, and outlier‐adjusted analyses remained consistent as outlined in Table S2. However, there was evidence suggestive of a bidirectional relationship between genetically predicted coronary artery disease and age at first birth (*b*=−0.029, SE=0.010, *P*=0.005). There was no evidence of other bidirectional associations. The full results of bidirectional analysis are outlined in Table S3. The associations remained consistent when evaluated on female‐specific outcome data from UK Biobank (Table S4), though additional associations were identified between age at first birth and atrial fibrillation (OR, 1.77 [95% CI, 1.31–2.39], *P*=2.11×10^−4^) and ischemic stroke (OR, 2.25 [95% CI, 1.29–3.93], *P*=0.004) that were consistent in direction with the other outcomes and main analysis. Additional adjustment for genetically predicted EA attenuated all associations between age at first birth and cardiovascular outcomes: coronary artery disease (OR, 1.30 [95% CI, 0.85–1.98], *P*=0.231), heart failure (OR, 0.93 [95% CI, 0.68–1.27], *P*=0.664) and stroke (OR, 1.01 [95% CI, 0.75–1.36], *P*=0.961), as reported in Table S5.

Earlier genetically predicted age at first birth was associated with higher BMI (*b*=0.357, SE=0.054, *P*=4.00×10^−11^), lower HDL (*b*=−0.216, SE=0.065, *P*=0.001), higher T2D odds (*b*=0.682, SE=0.138, *P*=8.24×10^−7^), and higher SBP (*b*=1.681, SE=0.506, *P*=0.001), identifying these factors as potential mediators as displayed in Table S6.

**Figure 3:**
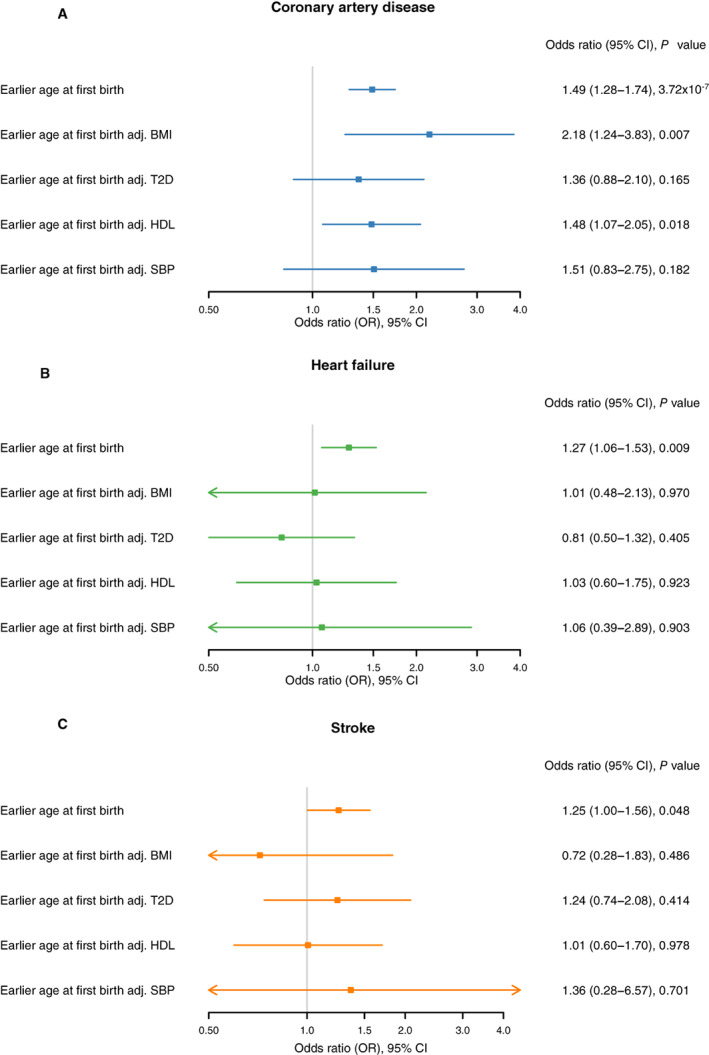
Mendelian randomization estimates for the effects of age at first birth on the cardiovascular outcomes significant on primary analysis, before and after adjustment for potential mediators. **A**, Mendelian randomization estimates for the effects of age at first birth on coronary artery disease, before and after adjustment for potential mediators. **B**, Mendelian randomization estimates for the effects of age at first birth on heart failure, before and after adjustment for potential mediators. **C**, Mendelian randomization estimates for the effects of age at first birth on stroke, before and after adjustment for potential mediators. Adj. indicates adjusted for; BMI, body mass index; HDL, high‐density lipoprotein; SBP, systolic blood pressure; and T2D, type 2 diabetes.

Mediation analysis for the association between genetically predicted age at first birth and coronary artery disease (unadjusted OR, 1.49 [95% CI, 1.28–1.74]) revealed some attenuation after adjustment for T2D (adjusted OR, 1.36 [95% CI, 0.88–2.10], *P*=0.165), suggesting T2D is a mediator, as reported in Table 3 and Figure 3. However, the instruments used in this mediation analysis were weak (all *F*‐statistics <10, as displayed in Table S7). Additional phenotypic associations for the instrumental SNPs are reported in Table S8.

**Table 3:** Mediation Analysis Results Using Multivariable Mendelian Randomization

Exposure	Outcome	Mediator adjusted for in analysis	#SNP	Odds ratio	Lower 95% CI	Upper 95% CI	*P* value
Age at first birth (per 1‐y reduction)	Coronary artery disease	None	18	1.49	1.28	1.74	3.72×10^−7^
		Body mass index	4	2.18	1.24	3.83	0.007
		High‐density lipoprotein cholesterol	6	1.48	1.07	2.05	0.018
		Type 2 diabetes	9	1.36	0.88	2.10	0.165
		Systolic blood pressure	3	1.51	0.83	2.75	0.182
	Heart failure	None	18	1.27	1.06	1.53	0.009
		Body mass index	4	1.01	0.48	2.13	0.970
		High‐density lipoprotein cholesterol	6	1.03	0.60	1.75	0.923
		Type 2 diabetes	9	0.81	0.50	1.32	0.405
		Systolic blood pressure	3	1.06	0.39	2.89	0.903
	Stroke	None	18	1.25	1.00	1.56	0.048
		Body mass index	4	0.72	0.28	1.83	0.486
		High‐density lipoprotein cholesterol	6	1.36	0.28	6.57	0.701
		Type 2 diabetes	11	1.24	0.74	2.08	0.414
		Systolic blood pressure	3	1.01	0.60	1.70	0.978
Age at menarche (per 1‐y reduction)	Coronary artery disease	None	198	1.10	1.06	1.14	1.68×10^−6^
		Body mass index	42	0.95	0.80	1.13	0.561
		Type 2 diabetes	71	1.10	0.97	1.25	0.124
	Heart failure	None	208	1.12	1.07	1.17	5.06×10^−7^
		Body mass index	42	0.98	0.79	1.21	0.827
		Type 2 diabetes	71	1.08	0.96	1.21	0.228

For each exposure‐outcome pair, the univariable, inverse‐variance weighted Mendelian randomization estimate of the marginal effect of the exposure on the outcome (“total” effect) is reported in the first row, and subsequently the effect of the exposure on the outcome that is conditional on each putative mediator (“direct” effect) is reported. #SNP indicates number of SNPs used in analysis; and SNP, single‐nucleotide polymorphism.

Mediation analysis for the association between genetically predicted age at first birth and heart failure (unadjusted OR, 1.27 [95% CI, 1.06–1.53]) revealed an attenuation of effect estimates after adjustment for BMI (adjusted OR, 1.01 [95% CI, 0.48–2.13], *P*=0.970), T2D (adjusted OR, 0.81 [95% CI, 0.50–1.32], *P*=0.405), HDL (adjusted OR, 1.03 [95% CI, 0.60–1.75], *P*=0.923), and SBP (adjusted OR, 1.06 [95% CI, 0.39–2.89], *P*=0.903), as reported in Table 3 and Figure 3.

Mediation analysis for the association between genetically predicted age at first birth and stroke (unadjusted OR, 1.25 [95% CI, 1.00–1.56]) revealed an attenuation of effect estimates after adjustment for BMI (adjusted OR, 0.72 [95% CI, 0.28–1.83], *P*=0.486), T2D (adjusted OR, 1.24 [95% CI, 0.74–2.08], *P*=0.414), and HDL (adjusted OR, 1.01 [95% CI, 0.60–1.70], *P*=0.978), as reported in Table 3 and Figure 3.

### Number of Live Births

Higher genetically predicted number of live births was associated with increased risk of heart failure (OR, 1.90 [95% CI, 1.28–2.82], *P*=0.001), ischemic stroke (OR, 1.86 [95% CI, 1.03–3.37], *P*=0.039) stroke of any type (OR, 2.07 [95% CI, 1.22–3.52], *P*=0.007), and increased risk of atrial fibrillation (OR per increase in category of <2, 2, or >2 live births, 2.91 [95% CI, 1.16–7.29], *P*=0.023) at nominal significance. Higher genetically predicted number of live births was not significantly associated with coronary artery disease (OR, 1.41 [95% CI, 1.00–2.00], *P*=0.051). Results are summarized in Table 2 and Figure 2.

Sensitivity analysis results were suggestive of pleiotropy in the association between genetically predicted number of live births and stroke (MR‐Egger intercept *P*=0.050). The remaining sensitivity analyses and outlier‐adjusted analyses remained consistent as outlined in Table S2. There was no evidence of bidirectional associations, as outlined in Table S3. The associations remained consistent when evaluated on female‐specific outcome data from UK Biobank (Table S4), though additional association were identified between number of live births and coronary artery disease (OR, 2.29 [95% CI, 1.10–4.74], *P*=0.026) that was consistent in direction with the other outcomes and main analysis. Additional adjustment for genetically predicted EA only attenuated the association between number of live births and atrial fibrillation (OR, 1.71 [95% CI, 0.43–6.85], *P*=0.451) but not for heart failure nor stroke, as reported in Table S5. Additional phenotypic associations for the SNPs used in this analysis are reported in Table S9.

Higher number of live births was not associated with BMI (*b*=0.069, SE=0.135, *P*=0.609), HDL (*b*=0.264, SE=0.418, *P*=0.527), low‐density lipoprotein cholesterol (*b*=0.867, SE=0.645, *P*=0.179), T2D (0.227, SE=0.322, *P*=0.481), or SBP (*b*=−2.946, SE=3.903, *P*=0.450) as displayed in Table S6, these factors were thus not carried forward to mediation analysis.

### Age at Menarche

Earlier genetically predicted age at menarche was associated with increased risk of coronary artery disease (OR per 1‐year earlier age, 1.10 [95% CI, 1.06–1.14], *P*=1.68×10^−6^) and increased risk of heart failure (OR, 1.12 [95% CI, 1.07–1.17], *P*=5.06×10^−7^). Earlier genetically predicted age at menarche was not associated with atrial fibrillation (OR, 1.01 [95% CI, 0.97–1.05], *P*=0.664), stroke (OR, 1.03 [95% CI, 0.98–1.08], *P*=0.222), or ischemic stroke (OR, 1.04 [95% CI, 0.98–1.09], *P*=0.182). The results are summarized in Table 2 and Figure 2.

Sensitivity analysis results were not suggestive of pleiotropy, and outlier‐adjusted estimates remained consistent as outlined in Table S2. There was no evidence of bidirectional associations, as outlined in Table S3. The associations remained consistent when evaluated on female‐specific outcome data from UK Biobank (Table S4). Additional adjustment for genetically predicted EA attenuated none of the associations between age at menarche and cardiovascular outcomes, as reported in Table S6. Additional phenotypic associations for the SNPs used in this analysis are reported in Table S10.

Earlier genetically predicted age at menarche was associated with BMI (*b*=0.145, SE=0.024, *P*=1.43×10^−9^) and T2D (*b*=0.173, SE=0.035, *P*=9.02×10^−7^), but there was no association with SBP (*b*=0.272, SE=0.178, *P*=0.127), HDL (*b*=−0.035, SE=0.020, *P*=0.078), or low‐density lipoprotein cholesterol (*b*=−0.003, SE=0.017, *P*=0.864), as displayed in Table S6. Mediation analysis was therefore carried out to explore potential mediation by BMI and T2D.

**Figure 4:**
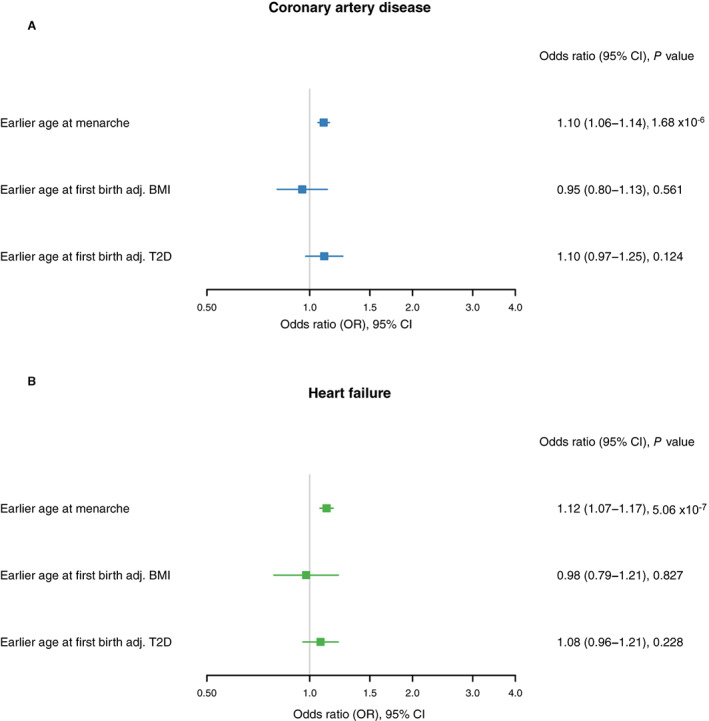
Mendelian randomization estimates for the effects of age at menarche on the cardiovascular outcomes significant on primary analysis, before and after adjustment for potential mediators. **A**, Mendelian randomization estimates for the effects of age at menarche on coronary artery disease, before and after adjustment for potential mediators. **B**, Mendelian randomization estimates for the effects of age at menarche on heart failure, before and after adjustment for potential mediators. Adj. indicates adjusted for; BMI, body mass index; and T2D, type 2 diabetes.

Mediation analysis for the association between age at menarche and coronary artery disease (unadjusted OR, 1.10 [95% CI, 1.06–1.14]) revealed an attenuation after adjustment for BMI (adjusted OR, 0.95 [95% CI, 0.80–1.13], *P*=0.561) but not T2D (adjusted OR, 1.10 [95% CI, 0.97–1.25], *P*=0.124), as presented in Figure 4 and Table 3.

Mediation analysis for the association between age at menarche and heart failure (unadjusted OR, 1.12 [95% CI, 1.07–1.17]) revealed an attenuation after adjustment for BMI (adjusted OR, 0.98 [95% CI, 0.79–1.21], *P*=0.827) and T2D (adjusted OR, 1.08 [95% CI, 0.96–1.21], *P*=0.228), as presented in Figure 4 and Table 3.

### Age at Menopause

Higher genetically predicted age at menopause was not associated with atrial fibrillation (OR, 1.00 [95% CI, 0.99–1.01], *P*=0.940), coronary artery disease (OR, 1.00 [95% CI, 0.99–1.01], *P*=0.894), heart failure (OR, 1.00 [95% CI, 0.99–1.01], *P*=0.735), ischemic stroke (OR, 1.00 [95% CI, 0.98–1.01], *P*=0.693), or stroke (OR, 1.00 [95% CI, 0.98–1.01], *P*=0.606). The results are summarized in Table 2 and Figure 2. The results were consistent on sensitivity analyses as displayed in Table S2. Because no associations were identified on primary analysis, mediation analysis was not carried out. Additional phenotypic associations for the SNPs used in the analysis are reported in Table S11.

## DISCUSSION

We used genetic epidemiology to evaluate the causal relevance of female reproductive factors on risk of multiple CVDs. Our results support an association between earlier age at first birth, higher number of live births, and earlier menarche, with higher risk of multiple CVDs, including atrial fibrillation, coronary artery disease, heart failure, and stroke. In most instances, these associations are of likely causal relevance, though some evidence suggestive of pleiotropy was observed for age at first birth and number of live births that likely relates to sociobehavioral traits such as EA. We also demonstrate important causal associations of reproductive factors with established cardiovascular risk markers such as obesity, diabetes, dyslipidemia, and hypertension. Mediation analyses suggested that these at least partly drive the augmented CVD caused by reproductive factors. Importantly, these mediators are amenable to clinical intervention. Overall, our results highlight the importance of taking a detailed reproductive history in women when assessing cardiovascular risk and highlight key opportunities for personalized preventive strategies.

### Age at First Birth and Number of Live Births

In this study, earlier genetically predicted age at first birth and higher genetically predicted number of live births were associated with increased risk of multiple CVD. It is well known that age at first birth and number of live births are tightly correlated, and an inverse genetic correlation between these factors has also been established.^18^ Broadly, our results demonstrating higher cardiovascular risk with a more “reproductive” phenotype corroborate observational findings^5^, ^6^, ^10^, ^16^, ^45^, ^46^, ^47^, ^48^, ^49^, ^50^ but additionally offer insight on the likely causal relevance of these factors. There are multiple potential mechanisms by which earlier first birth and higher numbers of live births might affct future cardiovascular risk.

The association between a more “reproductive” phenotype and CVD might result from direct effects of physiological changes that occur during pregnancy. These include changes that, at least temporarily, augment “traditional” cardiovascular risk factors including increased weight, hyperlipidemia, and insulin resistance but also other processes that promote CVD including heightened inflammatory profiles, more prothrombotic clotting function, and endothelial reactivity.^51^, ^52^, ^53^ Exposure to these factors for 9 months might suffice to increase cardiovascular risk. Repeated exposure across multiple pregnancies and the potential persistence of some of these changes beyond delivery is likely to augment risk, especially for some factors such as weight gain that require active motivation to reverse. The results of our study corroborate a key role of these cardiometabolic factors: we demonstrate that BMI, SBP, HDL, and T2D all mediate at least part of the associations between age and first birth and the outcomes of heart failure and stroke. This suggests a mechanistic relevance of these “traditional” risk factors. Beyond the mechanistic relevance, the mediating role of these factors should be considered clinically as it identifies an important opportunity for targeted risk stratification and prevention strategies.

A further potential explanation for the associations observed might be that the impact of at least some of the reproductive factors is not directly causal but rather might relate to other phenotypes that share a genetic basis with reproductive factors. This is a distinct possibility, in light of previous genetic studies that identified a close correlation between reproductive traits and multiple established social and behavioral risk factors for CVD: lower EA, higher adult risk tolerance, higher risk of attention deficit hyperactivity disorder and major depressive disorder, and earlier age at onset of smoking.^18^ In order to investigate this, we performed multivariable MR to account for the potential effects of EA, an established marker of social, behavioral, and economic status. The results of this analysis highlighted that EA, and therefore the broader sociobehavioral domain, is likely to account for at least part of the association between age at first birth and CVD. Though this suggests that at least a part of the association between reproductive factors and CVD is driven by pleiotropy, these associations still bear important clinical relevance for risk stratification. Our results demonstrate that, whether causal or not, age at first birth is able to capture the cardiovascular impact of a notoriously difficult‐to‐quantify sociobehavioral trajectory. Age at first birth, which is easy to measure, thus remains instrumental for quantifying a broad underlying set of circumstances that, when taken together, contribute to CVD risk in women.

Overall, the results of our study suggest that the association between a more “reproductive” phenotype and higher risk of CVD is likely to be driven by a combination of direct cardiometabolic effects of pregnancy and indirect effects of underlying sociobehavioral trajectories that share a genetic basis with reproductive behaviors. Specifically, the association between age at first birth and CVD appeared to strongly relate to EA, though we establish that this factor remains useful in clinical risk stratification as it captures the augmented cardiovascular risk conferred by sociobehavioral factors, which is otherwise difficult to quantify. On the other hand, the association between number of live births and CVD did not appear to be influenced by EA, highlighting a likely direct causal relevance of this factor. Finally, we identify multiple modifiable mediators of the association between reproductive factors and CVD, which should be key targets for clinical monitoring and personalized prevention.

### Age at Menarche

In our study, we identified an association between genetically predicted age at menarche and higher risk of both coronary artery disease and heart failure. Earlier menarche has been established as a predictor of cardiovascular risk in multiple studies.^5^, ^8^, ^9^, ^54^ However, age at menarche is closely correlated with childhood and adult‐life adiposity, and both are associated with higher risk of CVD. This makes BMI both an important potential confounder and a potential mediator. By design, MR mitigates the potential confounding role of childhood BMI, because childhood BMI cannot influence genetic liability to early menarche. Prior MR analyses have established an association between earlier age at menarche and higher coronary artery disease risk.^55^, ^56^, ^57^ The results of our study corroborate this evidence supporting a causal role of age at menarche on coronary artery disease on a larger study cohort and additionally provide evidence supporting a causal association of earlier age at menarche with heart failure.

Early menarche is known to be strongly associated with higher rates of obesity and metabolic ill health in adulthood, and this is a clear potential mediating pathway.^58^ In light of this well‐established association, we performed mediation analysis to explore the potential role of BMI and other cardiovascular risk factors in the association between age at menarche and CVD. This has not been done in prior MR studies. There was substantial evidence of mediation by BMI in for both coronary artery disease and heart failure. From a clinical perspective, the fact that adjustment for genetically predicted BMI in our study appeared to explain the vast majority of the increase in risk conferred by earlier age at menarche identifies BMI the chief driver of increased cardiovascular risk in women with early menarche. This should therefore be a major focus for primary prevention in women whose reproductive history features this factor.

### Age at Menopause

We investigated the impact of age at menopause on CVD. Observationally, earlier menopause has been associated with increased cardiovascular risk,^59^, ^60^ and this is postulated to be an effect of diminishing cardioprotective effects of estrogen. In our study, we had high statistical power to detect potentially small associations per year difference in timing of menopause, as reflected by the small CIs in the result, but there was no evidence of an association between age at menopause and CVDs. Considering the high power of this analysis, the results suggest that timing of menopause is unlikely to be causally related to CVD risk.

### Clinical Implications

Our results have important implications for both clinical risk stratification and targeted primary prevention strategies. In terms of risk stratification, these results suggest that reproductive history should be an important component of clinical evaluation of cardiovascular risk in women, given the multiple associations between reproductive factors and CVD of causal relevance. Even where we detect presence of pleiotropy and therefore suspect that some associations are not of causal relevance, information on reproductive factors is still likely to improve clinical risk stratification, because the underlying pleiotropic pathway is likely to relate to notoriously difficult‐to‐quantify metrics of a broad sociobehavioral and socioeconomic trajectory. Women are at particular risk from mischaracterization as low risk for cardiovascular risk, and the majority of those with CVD have an absence of traditional risk factors. However, despite the growing wealth of evidence supporting associations between reproductive factors and CVD,^61^ there is a paucity of evidence directly assessing the uplift in predictive performance of established clinical risk scores after additional incorporation of reproductive factors. Where 1 study exists on the outcome of heart failure,^62^ it was performed in a relatively small cohort and assessed only a few reproductive factors, and no diagnostic uplift was demonstrated. Given the results of our study, future work should imperatively focus on large‐scale assessment of the incremental benefit of addition of key reproductive factors to conventional cardiovascular risk stratification.

The results of this study can also help guide prevention strategies. Because reproductive factors such as age at menarche are not modifiable, and others such as age at first birth are unlikely to be realistically modifiable for the majority of women for the purpose of cardiovascular risk reduction, we explored multiple potentially modifiable mediators of the effect of reproductive factors on CVD. The rationale behind this was to establish the relevance of clinically “targetable” factors that can be monitored for, and aggressively managed, in order to curtail the augmented risk conferred by the reproductive factors. We demonstrate that the effects of age at menarche were substantially mediated by BMI. We also demonstrate that the effects of age at first birth on multiple CVDs were at least partly mediated by BMI, T2D, HDL, and SBP. Clinical surveillance of at‐risk women and early, aggressive management of these risk factors is a key priority that will at least partly mitigate the unfavorable effects of reproductive factors on CVD burden.

### Strengths and Limitations

The major strengths of this study stem from its genetic epidemiological approach, which mitigates the potential impact of confounding. In the hierarchy of evidence, MR has been advocated as providing “critical” evidence on risk factor–outcome relationships,^63^ especially when the risk factor in question is not practically or ethically amenable to randomization. The confidence with which causal relationships can be drawn from MR results depends on the plausibility of the instrumental variable assumptions for the selected genetic instruments. We explored these assumptions through checking instrument strength using F‐statistics, multiple sensitivity analyses more robust to pleiotropy, and bidirectional MR. This was used to distinguish the reproductive factors of causal relevance.

There are some limitations to consider. First, our analysis was carried out in populations of European ancestry; therefore, the results may not be generalizable to populations of other ancestries. Second, the second assumption of MR (of no existing confounders of the association between the variant and outcome) can be violated owing to population stratification. Population stratification can lead to a degree of confounding that is only avoidable through the use of within‐sibship GWASs. Although we attempted this, the largest available within‐sibship GWAS did not have sufficient instruments at genome‐wide significance level to allow analysis. This remains a target for further research when larger studies are available. Third, the lack of individual‐level data for the analyses is a limitation as summary‐level analysis is less flexible, which precludes exploration of potential nonlinear effects. This is an important target for future work, especially for the exposures of number of live births and age at first birth, for which prior observational studies have highlighted nonlinear associations with cardiovascular and mortality outcomes.^10^, ^64^ Fourth, there was partial sample overlap in the primary analyses and complete sample overlap in the sex‐specific sensitivity analyses for the exposures of age at first birth and number of live births. However, this is expected to have very limited impact on the results, bcause 2‐sample MR methods (except MR‐Egger) have been shown to produce reliable results in the setting of large biobanks even with complete sample overap.^65^ Finally, negative results in both univariable and multivariable analyses might be related to lack of a true causal association but might also be because of lack of sufficient statistical power. The results should therefore be interpreted in the context of instrument strength in all cases. This is particularly true for the mediation analyses, where attenuation to null was observed in some cases where instruments were weak (F‐statistics <10). Attenuation to the null in the mediation analyses should thus not be taken as indication of “full” mediation, as reaching the null invariably partly relates to a reduction in power.

## CONCLUSIONS

This study comprehensively explored the role and causal relevance of female‐specific reproductive risk factors on multiple CVDs, including atrial fibrillation, coronary artery disease, heart failure, and stroke. The findings support the emerging research focus on female‐specific risk factors for CVD, by demonstrating that earlier first birth, higher number of live births, and earlier menarche are all associated with increased CVD in women. Importantly, the associations for age at first birth are at least partly driven by pleiotropy through EA. By providing evidence to support the causal relevance of these factors, and additionally identifying key potential modifiable pathways to mitigate the increased risk that they entail, we stress the importance of routine evaluation of reproductive history in clinical risk stratification and consideration of targeted prevention strategies for women.

## Sources of Funding

This study was supported by the Medical Research Council (MRC scholarship for MA, United Kingdom Research and Innovation MRC MC_UU_00002/7 for EAWS and SB), the National Institute for Health Research (NIHR Cambridge Biomedical Research Centre BRC‐1215‐20014 for EAWS and SB, Imperial NIHR Biomedical Research Centre funding for FSN), and the British Heart Foundation (RG/16/3/32175 for FSN, British Heart Foundation Clinical Training Research Fellowship for PC). The views expressed are those of the authors and not necessarily those of the National Institute for Health Research or the Department of Health and Social Care.

## Disclosures

None.

## Supporting information

Tables S1–S11
