# Supplementary material for: Sex‐Specific Reproductive Factors Augment Cardiovascular Disease Risk in Women: A Mendelian Randomization Study
Source: J Am Heart Assoc. 2023 Feb 27;12(5):e027933. doi: 10.1161/JAHA.122.027933 (PMC10111460; doi:10.1161/JAHA.122.027933)
Supplement: Supplementary file 1 — Tables S1–S11 [file JAH3-12-e027933-s001.pdf]

SUPPLEMENTAL MATERIAL

Table S1 – Case definitions for study outcomes in the genome-wide association studies used for genetic association estimates. Further details are available at the individual study publications. EUR = European, ICD = International Classification of Diseases

Outcome	Case definition
**Atrial fibrillation**	HUNT: ICD-9 427.3 and ICD-10 I48 codes on hospital or outpatient record<br>deCODE: ICD-10 code I48 and ICD-9 code 427.3 on hospital record<br>MGI: ICD-9 427.31 billing code<br>DiscovEHR: ICD-10 I48 on electronic health record either as one problem code, or two separate diagnosis codes<br>UK Biobank: ICD-9 427.3 and ICD-10 I48 on healthcare record
**Coronary artery disease**	UKB: ICD10 codes I21-I25, OPCS-4 codes K40-K46, K49, K50 and K75<br>Cardiogram/C4D: See original publications
**Heart failure**	ARIC: ICD9 428.x code; ICD10 I50<br>BIOSTAT-CHF: Physician diagnosis<br>CHS: Self-report validated by doctor; or medical records<br>COGEN: LVEF<40% or clinical HF with NYHA>1 by clinician<br>deCODE: ICD9 428.x code; ICD10 I50<br>EGCUT: ICD10 I50 code<br>EPHESUS: Physician diagnosis<br>EPIC-Norfolk: ICD10 I50 code<br>FHS: Physician/clinical diagnosis<br>FINRISK: ICD-10: I50, I110, I130 and I132; ICD-9: 4029B, 404, 4148, 428; ICD-7: 42700, 42710, 428 or HF medication use<br>GODARTS: Physician/clinical diagnosis<br>GRADE: Physician/clinical diagnosis<br>LURIC: Physician/clinical diagnosis<br>MDCS: ICD8 427.00, 427.10, and 428.99; ICD9 428; ICD10 I50 and I11.0<br>PHFS: Physician/clinical diagnosis<br>PIVUS: ICD9 427.00, 427.10, 428; ICD10 I50 or I11.0<br>PREVEND: Physician/clinical diagnosis<br>PROSPER: Physician/clinical diagnosis<br>Regeneron/Geisinger:<br>Rotterdam study 1: Physician/clinical diagnosis<br>SHIP: Physician/clinical diagnosis<br>SOLID: Physician/clinical diagnosis<br>TwinGene: ICD-10: I50; ICD-8 and ICD-9 428<br>UK Biobank: ICD-10: I11.0, I13.0, I13.2, I25.5, I42.0, I42.5, I42.8, I42.9, I50.0, I50.1, I50.9; ICD-9: 4254, 4280, 4281, 4289<br>ULSAM: ICD9 427.00, 427.10, 428; ICD10 I50 or I11.0<br>WGHS: Physician/clinical diagnosis
**Stroke**	World Health Organization (WHO) definition: rapidly developing signs of focal/global disturbance of cerebral function, lasting more than 24 hours with no cause other than vascular.
**Ischemic stroke**	Stroke defined as above; ischaemic origin based on clinical and imaging criteria

Table S2 – Mendelian randomization (MR) sensitivity analyses for effects reproductive factors on cardiovascular outcomes, using weighted median MR, MR-PRESSO and MR-Egger method. CI = confidence interval, SNP= single nucleotide polymorphism, #SNP = number of SNPs used in analysis.

**Exposure**	**Outcome**	**Method**	**Odds ratio**	**Lower 95% CI**	**Upper 95% CI**	**P-value**
**Age at first birth** (per 1-year reduction)	Atrial fibrillation #SNP =18	Weighted median	1.00	0.83	1.21	0.999
		MR-PRESSO	1.10	0.95	1.26	0.216
		Mr-Egger	0.68	0.22	2.09	0.512
					intercept	0.471
	Coronary artery disease #SNP =18	Weighted median	1.58	1.33	1.88	2.02×10^{-7}
		MR-PRESSO	1.56	1.37	1.78	6.27×10^{-6}
		Mr-Egger	2.37	0.90	6.29	0.101
					intercept	0.356
	Heart failure #SNP =18	Weighted median	1.35	1.08	1.68	0.007
		MR-PRESSO	1.27	1.06	1.53	0.018
		Mr-Egger	1.18	0.38	3.72	0.776
					intercept	0.900
	Ischaemic stroke #SNP =18	Weighted median	1.30	0.98	1.72	0.068
		MR-PRESSO	1.16	0.92	1.47	0.219
		Mr-Egger	3.34	0.79	14.08	0.120
					intercept	0.166
	Stroke #SNP =18	Weighted median	1.35	1.03	1.78	0.032
		MR-PRESSO	1.25	1.00	1.56	0.066
		Mr-Egger	4.21	1.13	15.62	0.047
					intercept	0.085
**Number of live births** (per increase in category across <2, vs 2, vs >2 live births)	Atrial fibrillation #SNP =9	Weighted median	1.83	0.94	3.55	0.075
		MR-PRESSO	3.08	1.87	5.07	0.011
		Mr-Egger	0.28	0.00	11601	0.820
					intercept	0.677
	Coronary artery disease #SNP =9	Weighted median	1.33	0.93	1.92	0.120
		MR-PRESSO	1.41	1.00	2.00	0.087
		Mr-Egger	0.95	0.02	55.38	0.980
					intercept	0.853
	Heart failure #SNP =9	Weighted median	1.87	1.09	3.20	0.022
		MR-PRESSO	1.90	1.30	2.79	0.011

		Mr-Egger	0.59	0.01	69.57	0.835
					intercept	0.645
	Ischaemic stroke #SNP =9	Weighted median	1.65	0.81	3.35	0.165
		MR-PRESSO	1.86	1.03	3.37	0.077
		Mr-Egger	559	1.41	221492	0.077
					intercept	0.103
	Stroke #SNP =9	Weighted median	1.61	0.80	3.22	0.178
		MR-PRESSO	2.07	1.22	3.52	0.027
		Mr-Egger	1083	5.90	198842	0.034
					intercept	0.050
**Age at menarche** (per 1-year reduction)	Atrial fibrillation #SNP =208	Weighted median	0.95	0.90	1.00	0.045
		MR-PRESSO	0.99	0.96	1.03	0.638
		Mr-Egger	0.90	0.81	1.00	0.057
					intercept	0.024
	Coronary artery disease #SNP =198	Weighted median	1.07	1.02	1.12	0.003
		MR-PRESSO	1.09	1.06	1.13	2.71×10^{-7}
		Mr-Egger	1.08	0.98	1.19	0.115
					intercept	0.733
	Heart failure #SNP =208	Weighted median	1.11	1.04	1.18	0.001
		MR-PRESSO	1.11	1.06	1.15	2.68×10^{-6}
		Mr-Egger	1.08	0.97	1.21	0.174
					intercept	0.520
	Ischaemic stroke #SNP =208	Weighted median	1.05	0.98	1.13	0.170
		MR-PRESSO	1.04	0.99	1.09	0.114
		Mr-Egger	1.04	0.91	1.19	0.556
					intercept	0.933
	Stroke #SNP =208	Weighted median	1.02	0.96	1.09	0.499
		MR-PRESSO	1.03	0.99	1.08	0.167
		Mr-Egger	1.02	0.90	1.15	0.769
					intercept	0.846
**Age at menopause** (per 1-year increase)	Atrial fibrillation #SNP =154	Weighted median	1.00	0.99	1.01	0.932
		MR-PRESSO	1.00	0.99	1.01	0.548
		Mr-Egger	1.00	0.98	1.02	0.685
					intercept	0.664
		Weighted median	1.00	0.98	1.01	0.589

	Coronary artery disease #SNP =141	MR-PRESSO	1.00	0.99	1.01	0.813
		Mr-Egger	1.00	0.97	1.02	0.779
					intercept	0.804
	Heart failure #SNP =152	Weighted median	1.00	0.98	1.01	0.552
		MR-PRESSO	1.00	0.99	1.01	0.854
		Mr-Egger	1.00	0.98	1.02	0.883
					intercept	0.695
	Ischaemic stroke #SNP =151	Weighted median	0.99	0.96	1.01	0.220
		MR-PRESSO	1.00	0.98	1.01	0.613
		Mr-Egger	1.00	0.97	1.03	0.822
					intercept	0.610
	Stroke #SNP =151	Weighted median	0.98	0.96	1.00	0.685
		MR-PRESSO	1.00	0.98	1.01	0.545
		Mr-Egger	1.00	0.97	1.02	0.883
					intercept	0.881

Table S3 – Mendelian randomization (MR) sensitivity analysis to assess for bidirectional association, exploring the effects of cardiovascular disease on reproductive factors using inverse-variance weighted model with multiplicative random effects. Std. Error = standard error.

Exposure	Outcome	#SNP	Beta coefficient	Std. Error	P-value
**Atrial fibrillation** (per log(OR) increase)	Age at first birth	110	-0.009	0.009	0.317
	Age at menarche	110	-0.002	0.006	0.762
	Number of live births	110	0.003	0.004	0.521
**Coronary artery disease** (per log(OR) increase)	Age at first birth	143	-0.029	0.010	0.005
	Age at menarche	143	0.001	0.008	0.941
	Number of live births	143	0.009	0.005	0.061
**Heart failure** (per log(OR) increase)	Age at first birth	10	0.010	0.025	0.685
	Age at menarche	10	-0.082	0.066	0.214
	Number of live births	10	0.006	0.021	0.784
**Stroke** (per log(OR) increase)	Age at first birth	8	-0.005	0.034	0.888
	Age at menarche	8	0.007	0.017	0.694
	Number of live births	8	0.005	0.020	0.804
**Ischaemic stroke** (per log(OR) increase)	Age at first birth	10	-0.009	0.028	0.746
	Age at menarche	10	0.009	0.021	0.676
	Number of live births	10	-0.006	0.015	0.691

Table S4 – Sensitivity analysis using Mendelian randomization to estimate the effects of reproductive factors on cardiovascular outcomes, where SNP-outcome associations were calculated in the UK Biobank cohort restricted to female participants. CI = confidence interval.

**Exposure**	**Outcome**	**Odds ratio**	**Lower 95% CI**	**Upper 95% CI**	**P-value**
**Age at first birth** (per 1-year reduction)	Atrial fibrillation	1.77	1.31	2.39	2.11×10^{-4}
	Coronary artery disease	2.32	1.78	3.03	5.69×10^{-10}
	Heart failure	2.64	1.71	4.09	1.37×10^{-5}
	Ischaemic stroke	2.25	1.29	3.93	0.004
	Stroke	1.75	1.19	2.57	0.004
**Number of live births** (per increase in category across <2, vs 2, vs >2 live births)	Atrial fibrillation	3.19	1.42	7.19	0.005
	Coronary artery disease	2.29	1.10	4.74	0.026
	Heart failure	1.52	0.46	5.06	0.491
	Ischaemic stroke	2.54	0.43	14.87	0.302
	Stroke	3.22	0.82	12.57	0.093
**Age at menarche** (per 1-year reduction)	Atrial fibrillation	0.99	0.92	1.06	0.706
	Coronary artery disease	1.11	1.04	1.18	0.003
	Heart failure	1.15	1.05	1.27	0.005
	Ischaemic stroke	1.03	0.91	1.15	0.672
	Stroke	1.05	0.97	1.14	0.229
**Age at menopause** (per 1-year increase)	Atrial fibrillation	1.01	0.99	1.03	0.337
	Coronary artery disease	1.01	0.99	1.03	0.599
	Heart failure	1.01	0.98	1.04	0.651
	Ischaemic stroke	1.01	0.97	1.04	0.663
	Stroke	1.00	0.98	1.03	0.892

Table S5 – Sensitivity analysis using multivariable Mendelian randomization (MR) to estimate the effect of reproductive factors on cardiovascular outcomes after accounting for educational attainment. EA = Educational attainment, CI = confidence interval, SNP= single nucleotide polymorphism, #SNP = number of SNPs used in analysis.

Exposure	Outcome	Adjusted for	#SNP	Odds ratio	Lower 95% CI	Upper 95% CI	P-value
**Age at first birth** (per 1-year reduction)	Coronary artery disease	None	18	1.49	1.28	1.74	3.72×10^{-7}
		EA	11	1.30	0.85	1.98	0.231
	Heart failure	None	18	1.27	1.06	1.53	0.009
		EA	11	0.93	0.68	1.27	0.664
	Stroke	None	18	1.25	1.00	1.56	0.048
		EA	11	1.01	0.75	1.36	0.961
**Number of live births** (per 1-year reduction)	Atrial fibrillation	None	9	2.91	1.16	7.29	0.023
		EA	4	1.71	0.43	6.85	0.451
	Heart failure	None	9	1.90	1.28	2.82	0.001
		EA	4	1.77	0.97	3.21	0.062
	Ischaemic stroke	None	9	1.86	1.03	3.37	0.039
		EA	4	2.36	1.22	4.58	0.011
	Stroke	None	9	2.07	1.22	3.52	0.007
		EA	4	2.38	1.03	5.53	0.043
**Age at menarche** (per 1-year reduction)	Coronary artery disease	None	198	1.10	1.06	1.14	1.68×10^{-6}
		EA	81	1.23	1.11	1.36	5.00×10^{-5}
	Heart failure	None	208	1.12	1.07	1.17	5.06×10^{-7}
		EA	81	1.21	1.07	1.37	0.003

Table S6 – Mendelian randomization analyses to the association between reproductive factors and putative mediators using inverse-variance weighted model with multiplicative random effects. Only carried out for reproductive factors that displayed at least one nominally significant association with a cardiovascular outcome on primary analysis. Std. Error = standard error, SNP= single nucleotide polymorphism, #SNP = number of SNPs used in analysis.

Exposure	Outcome	#SNP	Beta coefficient	Std. Error	P-value	Used in mediation analysis?
**Age at first birth** (per 1-year reduction)	Body mass index	14	0.357	0.054	4.00×10^{-11}	Yes
	High-density lipoprotein cholesterol	12	-0.216	0.065	0.001	Yes
	Low-density lipoprotein cholesterol	12	-0.032	0.130	0.805	No
	Type 2 diabetes	16	0.682	0.138	8.24×10^{-7}	Yes
	Systolic blood pressure	17	1.681	0.506	0.001	Yes
**Number of live births** (per increase in category across <2, vs 2, vs >2 live births)	Body mass index	8	0.069	0.135	0.609	No
	High-density lipoprotein cholesterol	5	0.264	0.418	0.527	No
	Low-density lipoprotein cholesterol	5	0.867	0.645	0.179	No
	Type 2 diabetes	6	0.227	0.322	0.481	No
	Systolic blood pressure	8	-2.946	3.903	0.450	No
**Age at menarche** (per 1-year reduction)	Body mass index	109	0.145	0.024	1.43×10^{-9}	Yes
	High-density lipoprotein cholesterol	88	-0.035	0.020	0.078	No
	Low-density lipoprotein cholesterol	88	-0.003	0.017	0.864	No
	Type 2 diabetes	166	0.173	0.035	9.02×10^{-7}	Yes
	Systolic blood pressure	158	0.272	0.178	0.127	No

Table S7 – F-statistics for instrument strength in univariable and multivariable analyses.

Univariable analyses

**Exposure**	**Outcome**	**F-Statistic**
Age at first birth	Atrial fibrillation	38.89
	Coronary artery disease	38.89
	Heart failure	38.89
	Ischemic stroke	38.89
	Stroke	38.89
Number of live births	Atrial fibrillation	34.41
	Coronary artery disease	34.41
	Heart failure	34.41
	Ischemic stroke	34.41
	Stroke	34.41
Age at menarche	Atrial fibrillation	80.06
	Coronary artery disease	79.90
	Heart failure	80.06
	Ischemic stroke	80.06
	Stroke	80.06
Age at menopause	Atrial fibrillation	139.64
	Coronary artery disease	141.33
	Heart failure	140.06
	Ischemic stroke	140.57
	Stroke	140.57

Multivariable analyses

**Exposure**	**Outcome**	**Mediator**	**F-statistic**
Age at first birth	Coronary artery disease	Body mass index	7.50
		Systolic blood pressure	1.97
		High-density lipoprotein cholesterol	4.62
		Type 2 diabetes	4.82
Age at menarche	Coronary artery disease	Body mass index	19.14
		Type 2 diabetes	44.47
		Low-density lipoprotein cholesterol	26.87
Age at first birth	Heart failure	Body mass index	7.50
		Systolic blood pressure	36.70

		High-density lipoprotein cholesterol	75.73
		Type 2 diabetes	31.54
Age at menarche	Heart failure	Body mass index	19.14
		Type 2 diabetes	30.82
		Low-density lipoprotein cholesterol	27.17
Age at first birth	Stroke	Body mass index	7.49
		Systolic blood pressure	36.70
		High-density lipoprotein cholesterol	4.62
		Type 2 diabetes	31.92

Table S8 – Phenotype associations at genome-wide significance level ($p<5x10^{-8}$) of instrumental variants for age at first birth on PhenoScanner. SNP= single-nucleotide polymorphism, PMID = PubMed ID.

SNP	Alllele1	Allele2	Trait	Study	PMID/Source	Year
rs113905912	C	T	Alcohol usually taken with meals	Neale B	UKBB	2017
rs11669516	A	G	Comparative height size at age 10	Neale B	UKBB	2017
rs11669516	A	G	Height	GIANT	25282103	2014
rs11669516	A	G	Height	Neale B	UKBB	2017
rs11669516	A	G	Impedance of arm right	Neale B	UKBB	2017
rs11669516	A	G	Leg fat percentage left	Neale B	UKBB	2017
rs11669516	A	G	Leg fat percentage right	Neale B	UKBB	2017
rs11669516	A	G	Sitting height	Neale B	UKBB	2017
rs11669516	A	G	Total cholesterol	GLGC	24097068	2013
rs11669516	A	G	Total cholesterol	GLGC	20686565	2010
rs11669516	A	G	Total cholesterol	GLGC	20686565	2010
rs11669516	A	G	Triglycerides	GLGC	24097068	2013
rs11669516	A	G	Triglycerides	GLGC	20686565	2010
rs11669516	A	G	Triglycerides	GLGC	20686565	2010
rs12089815	A	G	Alcohol usually taken with meals	Neale B	UKBB	2017
rs12089815	A	G	Average weekly beer plus cider intake	Neale B	UKBB	2017
rs12089815	A	G	Body mass index	Neale B	UKBB	2017
rs12089815	A	G	Impedance of arm left	Neale B	UKBB	2017
rs12089815	A	G	Qualifications: A levels or as levels or equivalent	Neale B	UKBB	2017
rs12089815	A	G	Qualifications: college or university degree	Neale B	UKBB	2017
rs12089815	A	G	Qualifications: none	Neale B	UKBB	2017
rs12089815	A	G	Townsend deprivation index at recruitment	Neale B	UKBB	2017
rs12089815	A	G	Years of educational attainment	SSGAC	27225129	2016
rs2230590	T	C	Age completed full time education	Neale B	UKBB	2017
rs2230590	T	C	Alcohol intake frequency	Neale B	UKBB	2017
rs2230590	T	C	Alcohol intake versus 10 years previously	Neale B	UKBB	2017
rs2230590	T	C	Arm fat mass left	Neale B	UKBB	2017
rs2230590	T	C	Arm fat mass right	Neale B	UKBB	2017
rs2230590	T	C	Arm fat percentage left	Neale B	UKBB	2017
rs2230590	T	C	Arm fat percentage right	Neale B	UKBB	2017
rs2230590	T	C	Arm fat-free mass left	Neale B	UKBB	2017
rs2230590	T	C	Arm fat-free mass right	Neale B	UKBB	2017
rs2230590	T	C	Arm predicted mass left	Neale B	UKBB	2017
rs2230590	T	C	Arm predicted mass right	Neale B	UKBB	2017
rs2230590	T	C	Basal metabolic rate	Neale B	UKBB	2017
rs2230590	T	C	Body fat percentage	Neale B	UKBB	2017
rs2230590	T	C	Body mass index	GIANT	29273807	2018
rs2230590	T	C	Body mass index	GIANT	29273807	2018

rs2230590	T	C	Body mass index	Neale B	UKBB	2017
rs2230590	T	C	Crohns disease	IBDGC	23128233	2012
rs2230590	T	C	Diastolic blood pressure	Neale B	UKBB	2017
rs2230590	T	C	Fluid intelligence score	Neale B	UKBB	2017
rs2230590	T	C	Heel bone mineral density	Neale B	UKBB	2017
rs2230590	T	C	Hip circumference	Neale B	UKBB	2017
rs2230590	T	C	Impedance of arm left	Neale B	UKBB	2017
rs2230590	T	C	Impedance of arm right	Neale B	UKBB	2017
rs2230590	T	C	Impedance of leg left	Neale B	UKBB	2017
rs2230590	T	C	Impedance of leg right	Neale B	UKBB	2017
rs2230590	T	C	Impedance of whole body	Neale B	UKBB	2017
rs2230590	T	C	Inflammatory bowel disease	IBDGC	26192919	2015
rs2230590	T	C	Job involves heavy manual or physical work	Neale B	UKBB	2017
rs2230590	T	C	Job involves mainly walking or standing	Neale B	UKBB	2017
rs2230590	T	C	Leg fat mass left	Neale B	UKBB	2017
rs2230590	T	C	Leg fat mass right	Neale B	UKBB	2017
rs2230590	T	C	Leg fat percentage left	Neale B	UKBB	2017
rs2230590	T	C	Leg fat percentage right	Neale B	UKBB	2017
rs2230590	T	C	Leg fat-free mass left	Neale B	UKBB	2017
rs2230590	T	C	Leg fat-free mass right	Neale B	UKBB	2017
rs2230590	T	C	Leg predicted mass left	Neale B	UKBB	2017
rs2230590	T	C	Leg predicted mass right	Neale B	UKBB	2017
rs2230590	T	C	Miserableness	Neale B	UKBB	2017
rs2230590	T	C	Number of treatments or medications taken	Neale B	UKBB	2017
rs2230590	T	C	Overall health rating	Neale B	UKBB	2017
rs2230590	T	C	Qualifications: A levels or as levels or equivalent	Neale B	UKBB	2017
rs2230590	T	C	Qualifications: college or university degree	Neale B	UKBB	2017
rs2230590	T	C	Qualifications: none	Neale B	UKBB	2017
rs2230590	T	C	Taking other prescription medications	Neale B	UKBB	2017
rs2230590	T	C	Time spent watching television	Neale B	UKBB	2017
rs2230590	T	C	Trunk fat mass	Neale B	UKBB	2017
rs2230590	T	C	Trunk fat percentage	Neale B	UKBB	2017
rs2230590	T	C	Trunk fat-free mass	Neale B	UKBB	2017
rs2230590	T	C	Trunk predicted mass	Neale B	UKBB	2017
rs2230590	T	C	Usual walking pace	Neale B	UKBB	2017
rs2230590	T	C	Waist circumference	Neale B	UKBB	2017
rs2230590	T	C	Weight	Neale B	UKBB	2017
rs2230590	T	C	Wheeze or whistling in the chest in last year	Neale B	UKBB	2017
rs2230590	T	C	Whole body fat mass	Neale B	UKBB	2017
rs2230590	T	C	Whole body fat-free mass	Neale B	UKBB	2017
rs2230590	T	C	Whole body water mass	Neale B	UKBB	2017
rs2230590	T	C	Years of educational attainment	SSGAC	27225129	2016

rs2230590	T	C	Years of educational attainment in females	SSGAC	27225129	2016
rs2230590	T	C	Years of educational attainment in males	SSGAC	27225129	2016
rs2645977	A	G	Qualifications: college or university degree	Neale B	UKBB	2017
rs2667360	T	A	Body mass index	Neale B	UKBB	2017
rs2667360	T	A	Leg fat mass left	Neale B	UKBB	2017
rs2667360	T	A	Leg fat mass right	Neale B	UKBB	2017
rs2667360	T	A	Weight	Neale B	UKBB	2017
rs2667360	T	A	Whole body fat mass	Neale B	UKBB	2017
rs2667360	T	A	Years of educational attainment	SSGAC	27225129	2016
rs2667360	T	A	Years of educational attainment in females	SSGAC	27225129	2016
rs359240	A	G	Illness, injury, bereavement, stress in last 2 years: financial difficulties	Neale B	UKBB	2017
rs362307	T	C	Alcohol intake frequency	Neale B	UKBB	2017
rs362307	T	C	Arm fat mass left	Neale B	UKBB	2017
rs362307	T	C	Arm fat mass right	Neale B	UKBB	2017
rs362307	T	C	Arm fat percentage left	Neale B	UKBB	2017
rs362307	T	C	Arm fat percentage right	Neale B	UKBB	2017
rs362307	T	C	Body mass index	Neale B	UKBB	2017
rs362307	T	C	Drive faster than motorway speed limit	Neale B	UKBB	2017
rs362307	T	C	Leg fat mass left	Neale B	UKBB	2017
rs362307	T	C	Leg fat mass right	Neale B	UKBB	2017
rs362307	T	C	Leg fat percentage left	Neale B	UKBB	2017
rs362307	T	C	Leg fat percentage right	Neale B	UKBB	2017
rs362307	T	C	Overall health rating	Neale B	UKBB	2017
rs362307	T	C	Qualifications: college or university degree	Neale B	UKBB	2017
rs3757323	C	T	Height	Neale B	UKBB	2017
rs3757323	C	T	Qualifications: college or university degree	Neale B	UKBB	2017
rs4799936	A	G	Depressive symptoms	SSGAC	29292387	2018
rs4799936	A	G	Depressive symptoms	SSGAC	29292387	2018
rs4799936	A	G	Depressive symptoms multi trait analysis	SSGAC	29292387	2018
rs4799936	A	G	Overall health rating	Neale B	UKBB	2017
rs4799936	A	G	Qualifications: college or university degree	Neale B	UKBB	2017
rs4799936	A	G	Qualifications: none	Neale B	UKBB	2017
rs4799936	A	G	Sensitivity or hurt feelings	Neale B	UKBB	2017
rs72829857	A	G	Qualifications: A levels or as levels or equivalent	Neale B	UKBB	2017
rs72829857	A	G	Qualifications: college or university degree	Neale B	UKBB	2017
rs9372625	A	G	Alcohol intake frequency	Neale B	UKBB	2017
rs9372625	A	G	Arm fat mass left	Neale B	UKBB	2017
rs9372625	A	G	Arm fat mass right	Neale B	UKBB	2017
rs9372625	A	G	Arm fat percentage left	Neale B	UKBB	2017
rs9372625	A	G	Arm fat percentage right	Neale B	UKBB	2017
rs9372625	A	G	Average weekly red wine intake	Neale B	UKBB	2017
rs9372625	A	G	Body fat percentage	Neale B	UKBB	2017

rs9372625	A	G	Body mass index	Neale B	UKBB	2017
rs9372625	A	G	Fluid intelligence score	Neale B	UKBB	2017
rs9372625	A	G	Job involves heavy manual or physical work	Neale B	UKBB	2017
rs9372625	A	G	Job involves mainly walking or standing	Neale B	UKBB	2017
rs9372625	A	G	Leg fat mass left	Neale B	UKBB	2017
rs9372625	A	G	Leg fat mass right	Neale B	UKBB	2017
rs9372625	A	G	Leg fat percentage left	Neale B	UKBB	2017
rs9372625	A	G	Leg fat percentage right	Neale B	UKBB	2017
rs9372625	A	G	Overall health rating	Neale B	UKBB	2017
rs9372625	A	G	Qualifications: A levels or as levels or equivalent	Neale B	UKBB	2017
rs9372625	A	G	Qualifications: college or university degree	Neale B	UKBB	2017
rs9372625	A	G	Qualifications: CSEs or equivalent	Neale B	UKBB	2017
rs9372625	A	G	Qualifications: none	Neale B	UKBB	2017
rs9372625	A	G	Qualifications: O levels or GCSEs or equivalent	Neale B	UKBB	2017
rs9372625	A	G	Time spent using computer	Neale B	UKBB	2017
rs9372625	A	G	Time spent watching television	Neale B	UKBB	2017
rs9372625	A	G	Trunk fat mass	Neale B	UKBB	2017
rs9372625	A	G	Trunk fat percentage	Neale B	UKBB	2017
rs9372625	A	G	Whole body fat mass	Neale B	UKBB	2017
rs9372625	A	G	Years of educational attainment	SSGAC	27225129	2016
rs9372625	A	G	Years of educational attainment in females	SSGAC	27225129	2016
rs9372625	A	G	Years of educational attainment in males	SSGAC	27225129	2016

Table S9 – Phenotype associations at genome-wide significance level ($p<5x10^{-8}$) of instrumental variants for number of live births on PhenoScanner. SNP= single-nucleotide polymorphism, PMID = PubMed ID.

SNP	Alllele1	Allele2	Trait	Study	PMID/Source	Year
rs116956554	A	G	Eosinophil count	Astle W	27863252	2016
rs116956554	A	G	Eosinophil percentage of granulocytes	Astle W	27863252	2016
rs116956554	A	G	Eosinophil percentage of white cells	Astle W	27863252	2016
rs116956554	A	G	Hematocrit	Astle W	27863252	2016
rs116956554	A	G	Hemoglobin concentration	Astle W	27863252	2016
rs116956554	A	G	High grade serous ovarian cancer	Phelan M	28346442	2017
rs116956554	A	G	High light scatter reticulocyte count	Astle W	27863252	2016
rs116956554	A	G	Invasive ovarian cancer	Phelan M	28346442	2017
rs116956554	A	G	Lymphocyte percentage of white cells	Astle W	27863252	2016
rs116956554	A	G	Neutrophil percentage of granulocytes	Astle W	27863252	2016
rs116956554	A	G	Neutrophil percentage of white cells	Astle W	27863252	2016
rs116956554	A	G	Red blood cell count	Astle W	27863252	2016
rs116956554	A	G	Red cell distribution width	Astle W	27863252	2016
rs116956554	A	G	Reticulocyte count	Astle W	27863252	2016
rs116956554	A	G	Serous invasive ovarian cancer	Phelan M	28346442	2017
rs116956554	A	G	Sum eosinophil basophil counts	Astle W	27863252	2016
rs1496108	G	A	Heel bone mineral density	Neale B	UKBB	2017
rs1496108	G	A	Heel bone mineral density left	Neale B	UKBB	2017
rs1496108	G	A	Heel bone mineral density right	Neale B	UKBB	2017
rs174557	A	G	Eosinophil count	Astle W	27863252	2016
rs174557	A	G	Granulocyte count	Astle W	27863252	2016
rs174557	A	G	Granulocyte percentage of myeloid white cells	Astle W	27863252	2016
rs174557	A	G	Hematocrit	Astle W	27863252	2016
rs174557	A	G	Hemoglobin concentration	Astle W	27863252	2016
rs174557	A	G	Mean corpuscular volume	Astle W	27863252	2016
rs174557	A	G	Mean platelet volume	Astle W	27863252	2016
rs174557	A	G	Monocyte percentage of white cells	Astle W	27863252	2016
rs174557	A	G	Myeloid white cell count	Astle W	27863252	2016
rs174557	A	G	Neutrophil count	Astle W	27863252	2016
rs174557	A	G	Platelet count	Astle W	27863252	2016
rs174557	A	G	Red blood cell count	Astle W	27863252	2016
rs174557	A	G	Red cell distribution width	Astle W	27863252	2016
rs174557	A	G	Sum basophil neutrophil counts	Astle W	27863252	2016
rs174557	A	G	Sum eosinophil basophil counts	Astle W	27863252	2016
rs174557	A	G	Sum neutrophil eosinophil counts	Astle W	27863252	2016
rs174557	A	G	White blood cell count	Astle W	27863252	2016
rs2044725	T	C	Arm fat mass left	Neale B	UKBB	2017
rs2044725	T	C	Arm fat-free mass left	Neale B	UKBB	2017
rs2044725	T	C	Arm fat-free mass right	Neale B	UKBB	2017
rs2044725	T	C	Arm predicted mass left	Neale B	UKBB	2017
rs2044725	T	C	Arm predicted mass right	Neale B	UKBB	2017
rs2044725	T	C	Basal metabolic rate	Neale B	UKBB	2017
rs2044725	T	C	Body mass index	Neale B	UKBB	2017
rs2044725	T	C	Comparative body size at age 10	Neale B	UKBB	2017

rs2044725	T	C	Drive faster than motorway speed limit	Neale B	UKBB	2017
rs2044725	T	C	Ever smoked	Neale B	UKBB	2017
rs2044725	T	C	Hand grip strength left	Neale B	UKBB	2017
rs2044725	T	C	Impedance of arm left	Neale B	UKBB	2017
rs2044725	T	C	Impedance of arm right	Neale B	UKBB	2017
rs2044725	T	C	Impedance of leg left	Neale B	UKBB	2017
rs2044725	T	C	Impedance of leg right	Neale B	UKBB	2017
rs2044725	T	C	Impedance of whole body	Neale B	UKBB	2017
rs2044725	T	C	Leg fat mass left	Neale B	UKBB	2017
rs2044725	T	C	Leg fat mass right	Neale B	UKBB	2017
rs2044725	T	C	Leg fat-free mass left	Neale B	UKBB	2017
rs2044725	T	C	Leg fat-free mass right	Neale B	UKBB	2017
rs2044725	T	C	Leg predicted mass left	Neale B	UKBB	2017
rs2044725	T	C	Leg predicted mass right	Neale B	UKBB	2017
rs2044725	T	C	Nervous feelings	Neale B	UKBB	2017
rs2044725	T	C	Past tobacco smoking	Neale B	UKBB	2017
rs2044725	T	C	Risk taking	Neale B	UKBB	2017
rs2044725	T	C	Smoking status: previous	Neale B	UKBB	2017
rs2044725	T	C	Suffer from nerves	Neale B	UKBB	2017
rs2044725	T	C	Trunk fat-free mass	Neale B	UKBB	2017
rs2044725	T	C	Trunk predicted mass	Neale B	UKBB	2017
rs2044725	T	C	Weight	Neale B	UKBB	2017
rs2044725	T	C	Whole body fat-free mass	Neale B	UKBB	2017
rs2044725	T	C	Whole body water mass	Neale B	UKBB	2017
rs2044725	T	C	Worrier or anxious feelings	Neale B	UKBB	2017
rs4838926	C	G	Self-reported atrial fibrillation	Neale B	UKBB	2017
rs7515106	C	T	Female genital prolapse	Neale B	UKBB	2017
rs7515106	C	T	Forced vital capacity	Neale B	UKBB	2017
rs7515106	C	T	Forced vital capacity, best measure	Neale B	UKBB	2017
rs7515106	C	T	Heel bone mineral density	Neale B	UKBB	2017
rs7515106	C	T	Heel bone mineral density left	Neale B	UKBB	2017
rs7515106	C	T	Heel bone mineral density right	Neale B	UKBB	2017
rs7515106	C	T	Hip circumference	Neale B	UKBB	2017
rs7515106	C	T	Impedance of leg left	Neale B	UKBB	2017
rs7515106	C	T	Impedance of leg right	Neale B	UKBB	2017
rs7515106	C	T	Leg fat-free mass left	Neale B	UKBB	2017
rs7515106	C	T	Leg fat-free mass right	Neale B	UKBB	2017
rs7515106	C	T	Leg predicted mass left	Neale B	UKBB	2017
rs7515106	C	T	Leg predicted mass right	Neale B	UKBB	2017
rs7515106	C	T	Sitting height	Neale B	UKBB	2017

Table S10 – Phenotype associations at genome-wide significance level ($p<5x10^{-8}$) of instrumental variants for age at menarche on PhenoScanner. SNP= single-nucleotide polymorphism, PMID = PubMed ID.

SNP	Alllele 1	Allele 2	Trait	Study	PMID/Source	Year
rs10136330	C	T	Comparative body size at age 10	Neale B	UKBB	2017
rs10138913	C	T	Age at menarche	Neale B	UKBB	2017
rs10138913	C	T	Arm fat-free mass left	Neale B	UKBB	2017
rs10138913	C	T	Arm fat-free mass right	Neale B	UKBB	2017
rs10138913	C	T	Arm predicted mass left	Neale B	UKBB	2017
rs10138913	C	T	Arm predicted mass right	Neale B	UKBB	2017
rs10138913	C	T	Basal metabolic rate	Neale B	UKBB	2017
rs10138913	C	T	Comparative height size at age 10	Neale B	UKBB	2017
rs10138913	C	T	Height	Neale B	UKBB	2017
rs10138913	C	T	Hip circumference	Neale B	UKBB	2017
rs10138913	C	T	Impedance of leg right	Neale B	UKBB	2017
rs10138913	C	T	Leg fat-free mass left	Neale B	UKBB	2017
rs10138913	C	T	Leg fat-free mass right	Neale B	UKBB	2017
rs10138913	C	T	Leg predicted mass left	Neale B	UKBB	2017
rs10138913	C	T	Leg predicted mass right	Neale B	UKBB	2017
rs10138913	C	T	Sitting height	Neale B	UKBB	2017
rs10138913	C	T	Trunk fat-free mass	Neale B	UKBB	2017
rs10138913	C	T	Trunk predicted mass	Neale B	UKBB	2017
rs10138913	C	T	Weight	Neale B	UKBB	2017
rs10138913	C	T	Whole body fat-free mass	Neale B	UKBB	2017
rs10138913	C	T	Whole body water mass	Neale B	UKBB	2017
rs10143972	C	T	Body mass index	Neale B	UKBB	2017
rs10156597	A	T	Height	GIANT	25282103	2014
rs10156597	A	T	Age at menarche	Neale B	UKBB	2017
rs10156597	A	T	Forced expiratory volume in 1-second	Neale B	UKBB	2017
rs10156597	A	T	Forced vital capacity	Neale B	UKBB	2017
rs10156597	A	T	Forced vital capacity, best measure	Neale B	UKBB	2017
rs10156597	A	T	Height	Neale B	UKBB	2017
rs10156597	A	T	Relative age of first facial hair	Neale B	UKBB	2017
rs10156597	A	T	Relative age voice broke	Neale B	UKBB	2017
rs10156597	A	T	Age at menarche	ReproGen	25231870	2014
rs10237306	G	T	Arm fat mass left	Neale B	UKBB	2017
rs10237306	G	T	Arm fat mass right	Neale B	UKBB	2017
rs10237306	G	T	Arm fat-free mass left	Neale B	UKBB	2017
rs10237306	G	T	Arm fat-free mass right	Neale B	UKBB	2017
rs10237306	G	T	Arm predicted mass left	Neale B	UKBB	2017
rs10237306	G	T	Arm predicted mass right	Neale B	UKBB	2017
rs10237306	G	T	Basal metabolic rate	Neale B	UKBB	2017
rs10237306	G	T	Height	Neale B	UKBB	2017
rs10237306	G	T	Hip circumference	Neale B	UKBB	2017
rs10237306	G	T	Leg fat mass left	Neale B	UKBB	2017
rs10237306	G	T	Leg fat mass right	Neale B	UKBB	2017

rs10237306	G	T	Leg fat-free mass left	Neale B	UKBB	2017
rs10237306	G	T	Leg fat-free mass right	Neale B	UKBB	2017
rs10237306	G	T	Leg predicted mass left	Neale B	UKBB	2017
rs10237306	G	T	Leg predicted mass right	Neale B	UKBB	2017
rs10237306	G	T	Trunk fat mass	Neale B	UKBB	2017
rs10237306	G	T	Trunk fat-free mass	Neale B	UKBB	2017
rs10237306	G	T	Trunk predicted mass	Neale B	UKBB	2017
rs10237306	G	T	Waist circumference	Neale B	UKBB	2017
rs10237306	G	T	Weight	Neale B	UKBB	2017
rs10237306	G	T	Whole body fat mass	Neale B	UKBB	2017
rs10237306	G	T	Whole body fat-free mass	Neale B	UKBB	2017
rs10237306	G	T	Whole body water mass	Neale B	UKBB	2017
rs1023955	G	T	Age at menarche	Neale B	UKBB	2017
rs1023955	G	T	Arm fat mass left	Neale B	UKBB	2017
rs1023955	G	T	Arm fat mass right	Neale B	UKBB	2017
rs1023955	G	T	Arm fat percentage left	Neale B	UKBB	2017
rs1023955	G	T	Arm fat percentage right	Neale B	UKBB	2017
rs1023955	G	T	Body fat percentage	Neale B	UKBB	2017
rs1023955	G	T	Body mass index	Neale B	UKBB	2017
rs1023955	G	T	Hip circumference	Neale B	UKBB	2017
rs1023955	G	T	Leg fat mass left	Neale B	UKBB	2017
rs1023955	G	T	Leg fat mass right	Neale B	UKBB	2017
rs1023955	G	T	Leg fat percentage left	Neale B	UKBB	2017
rs1023955	G	T	Leg fat percentage right	Neale B	UKBB	2017
rs1023955	G	T	Trunk fat mass	Neale B	UKBB	2017
rs1023955	G	T	Trunk fat percentage	Neale B	UKBB	2017
rs1023955	G	T	Types of physical activity in last 4 weeks: walking for pleasure	Neale B	UKBB	2017
rs1023955	G	T	Waist circumference	Neale B	UKBB	2017
rs1023955	G	T	Weight	Neale B	UKBB	2017
rs1023955	G	T	Whole body fat mass	Neale B	UKBB	2017
rs1025128	G	C	Height	Neale B	UKBB	2017
rs1025128	G	C	Qualifications: college or university degree	Neale B	UKBB	2017
rs1040070	G	C	Childhood BMI	EGGC	26604143	2016
rs1040070	G	C	Childhood obesity	EGGC	22484627	2012
rs1040070	G	C	Body mass index in females less than or equal to 50 years of age	GIANT	26426971	2015
rs1040070	G	C	Body mass index in females	GIANT	23754948	2013
rs1040070	G	C	Body mass index in females	GIANT	25673413	2015
rs1040070	G	C	Body mass index adjusted for physical activity	GIANT	28448500	2017
rs1040070	G	C	Body mass index adjusted for physical activity	GIANT	28448500	2017
rs1040070	G	C	Body mass index adjusted for smoking	GIANT	28443625	2017
rs1040070	G	C	Body mass index	Speliotes EK	20935630	2010
rs1040070	G	C	Body mass index	GIANT	23754948	2013
rs1040070	G	C	Body mass index	GIANT	25673413	2015
rs1040070	G	C	Body mass index	GIANT	25673413	2015
rs1040070	G	C	Hip circumference	GIANT	25673412	2015
rs1040070	G	C	Hip circumference	GIANT	25673412	2015
rs1040070	G	C	Waist circumference	GIANT	25673412	2015

rs1040070	G	C	Body mass index	Speliotes EK	20935630	2010
rs1040070	G	C	Obesity with early age of onset	EGGC	22484627	2012
rs1040070	G	C	Obesity with early age of onset age 2	EGGC	22484627	2012
rs1040070	G	C	Menarche age at onset	Pickrell JK	27182965	2016
rs1040070	G	C	Age at menarche	Neale B	UKBB	2017
rs1040070	G	C	Arm fat mass left	Neale B	UKBB	2017
rs1040070	G	C	Arm fat mass right	Neale B	UKBB	2017
rs1040070	G	C	Arm fat percentage left	Neale B	UKBB	2017
rs1040070	G	C	Arm fat percentage right	Neale B	UKBB	2017
rs1040070	G	C	Basal metabolic rate	Neale B	UKBB	2017
rs1040070	G	C	Body mass index	Neale B	UKBB	2017
rs1040070	G	C	Comparative body size at age 10	Neale B	UKBB	2017
rs1040070	G	C	Comparative height size at age 10	Neale B	UKBB	2017
rs1040070	G	C	Hip circumference	Neale B	UKBB	2017
rs1040070	G	C	Impedance of arm left	Neale B	UKBB	2017
rs1040070	G	C	Impedance of leg left	Neale B	UKBB	2017
rs1040070	G	C	Impedance of leg right	Neale B	UKBB	2017
rs1040070	G	C	Impedance of whole body	Neale B	UKBB	2017
rs1040070	G	C	Leg fat-free mass left	Neale B	UKBB	2017
rs1040070	G	C	Leg fat-free mass right	Neale B	UKBB	2017
rs1040070	G	C	Leg predicted mass left	Neale B	UKBB	2017
rs1040070	G	C	Leg predicted mass right	Neale B	UKBB	2017
rs1040070	G	C	Relative age voice broke	Neale B	UKBB	2017
rs1040070	G	C	Weight	Neale B	UKBB	2017
rs1040070	G	C	Age at menarche	ReproGen	25231870	2014
rs10750766	A	C	High light scatter percentage of red cells	Astle W	27863252	2016
rs10750766	A	C	High light scatter reticulocyte count	Astle W	27863252	2016
rs10750766	A	C	Immature fraction of reticulocytes	Astle W	27863252	2016
rs10750766	A	C	High light scatter reticulocyte count	Astle W	27863252	2016
rs10750766	A	C	High light scatter reticulocyte percentage of red cells	Astle W	27863252	2016
rs10750766	A	C	Immature fraction of reticulocytes	Astle W	27863252	2016
rs10750766	A	C	Diastolic blood pressure	Neale B	UKBB	2017
rs10750766	A	C	Heel bone mineral density	Neale B	UKBB	2017
rs10750766	A	C	Heel bone mineral density right	Neale B	UKBB	2017
rs10750766	A	C	Self-reported hypertension	Neale B	UKBB	2017
rs10750766	A	C	Treatment with blood pressure medication	Neale B	UKBB	2017
rs10750766	A	C	Vascular or heart problems diagnosed by doctor: high blood pressure	Neale B	UKBB	2017
rs10750766	A	C	Vascular or heart problems diagnosed by doctor: none of the above	Neale B	UKBB	2017
rs1079866	G	C	Age at menarche	Elks CE	21102462	2010
rs1079866	G	C	Menarche age at onset	Elks CE	21102462	2010
rs1079866	G	C	Menarche age at onset	ReproGen	25231870	2014
rs1079866	G	C	Age at menarche	Neale B	UKBB	2017
rs1079866	G	C	Age at menarche	ReproGen	25231870	2014
rs1079866	G	C	Menarche	Elks CE	21102462	2010
rs10832021	A	G	Age at menarche	Neale B	UKBB	2017
rs10832021	A	G	Body mass index	Neale B	UKBB	2017

rs10832021	A	G	Height	Neale B	UKBB	2017
rs10832021	A	G	Worry too long after embarrassment	Neale B	UKBB	2017
rs10832021	A	G	Age at menarche	ReproGen	25231870	2014
rs10931831	C	T	Age at menarche	Neale B	UKBB	2017
rs10931831	C	T	Impedance of leg left	Neale B	UKBB	2017
rs10931831	C	T	Impedance of leg right	Neale B	UKBB	2017
rs10931831	C	T	Impedance of whole body	Neale B	UKBB	2017
rs10934420	C	T	Age at menarche	Neale B	UKBB	2017
rs10934420	C	T	Height	Neale B	UKBB	2017
rs10934420	C	T	Age at menarche	ReproGen	25231870	2014
rs11031040	G	T	Bilateral oophorectomy	Neale B	UKBB	2017
rs11031040	G	T	Excessive, frequent and irregular menstruation	Neale B	UKBB	2017
rs11031040	G	T	Length of menstrual cycle	Neale B	UKBB	2017
rs11065822	G	T	Eosinophil count	Astle W	27863252	2016
rs11065822	G	T	Eosinophil percentage of granulocytes	Astle W	27863252	2016
rs11065822	G	T	Eosinophil percentage of white cells	Astle W	27863252	2016
rs11065822	G	T	Hematocrit	Astle W	27863252	2016
rs11065822	G	T	Hemoglobin concentration	Astle W	27863252	2016
rs11065822	G	T	High light scatter percentage of red cells	Astle W	27863252	2016
rs11065822	G	T	High light scatter reticulocyte count	Astle W	27863252	2016
rs11065822	G	T	Immature fraction of reticulocytes	Astle W	27863252	2016
rs11065822	G	T	Lymphocyte count	Astle W	27863252	2016
rs11065822	G	T	Lymphocyte percentage of white cells	Astle W	27863252	2016
rs11065822	G	T	Monocyte count	Astle W	27863252	2016
rs11065822	G	T	Neutrophil percentage of granulocytes	Astle W	27863252	2016
rs11065822	G	T	Neutrophil percentage of white cells	Astle W	27863252	2016
rs11065822	G	T	Platelet count	Astle W	27863252	2016
rs11065822	G	T	Plateletcrit	Astle W	27863252	2016
rs11065822	G	T	Red blood cell count	Astle W	27863252	2016
rs11065822	G	T	Reticulocyte count	Astle W	27863252	2016
rs11065822	G	T	Reticulocyte fraction of red cells	Astle W	27863252	2016
rs11065822	G	T	Sum eosinophil basophil counts	Astle W	27863252	2016
rs11065822	G	T	White blood cell count	Astle W	27863252	2016
rs11065822	G	T	Arm fat-free mass left	Neale B	UKBB	2017
rs11065822	G	T	Arm fat-free mass right	Neale B	UKBB	2017
rs11065822	G	T	Arm predicted mass left	Neale B	UKBB	2017
rs11065822	G	T	Arm predicted mass right	Neale B	UKBB	2017
rs11065822	G	T	Basal metabolic rate	Neale B	UKBB	2017
rs11065822	G	T	Birth weight	Neale B	UKBB	2017
rs11065822	G	T	Diastolic blood pressure	Neale B	UKBB	2017
rs11065822	G	T	Impedance of arm left	Neale B	UKBB	2017
rs11065822	G	T	Impedance of arm right	Neale B	UKBB	2017
rs11065822	G	T	Impedance of whole body	Neale B	UKBB	2017
rs11065822	G	T	Self-reported hypertension	Neale B	UKBB	2017
rs11065822	G	T	Self-reported hypothyroidism or myxoedema	Neale B	UKBB	2017
rs11065822	G	T	Treatment with levothyroxine sodium	Neale B	UKBB	2017
rs11065822	G	T	Trunk fat-free mass	Neale B	UKBB	2017
rs11065822	G	T	Trunk predicted mass	Neale B	UKBB	2017

rs11065822	G	T	Vascular or heart problems diagnosed by doctor: high blood pressure	Neale B	UKBB	2017
rs11065822	G	T	Vascular or heart problems diagnosed by doctor: none of the above	Neale B	UKBB	2017
rs11065822	G	T	Whole body fat-free mass	Neale B	UKBB	2017
rs11065822	G	T	Whole body water mass	Neale B	UKBB	2017
rs11065822	G	T	Coronary artery disease	van der Harst P	29212778	2018
rs11065822	G	T	Coronary artery disease	van der Harst P	29212778	2018
rs11165924	A	G	Menarche age at onset	ReproGen	25231870	2014
rs11165924	A	G	Age at menarche	ReproGen	25231870	2014
rs11209331	C	T	Height	GIANT	20881960	2010
rs11209331	C	T	Height	GIANT	23754948	2013
rs11209331	C	T	Height	GIANT	25282103	2014
rs11209331	C	T	Height	GIANT	20881960	2010
rs11209331	C	T	Arm fat-free mass left	Neale B	UKBB	2017
rs11209331	C	T	Arm fat-free mass right	Neale B	UKBB	2017
rs11209331	C	T	Arm predicted mass left	Neale B	UKBB	2017
rs11209331	C	T	Arm predicted mass right	Neale B	UKBB	2017
rs11209331	C	T	Basal metabolic rate	Neale B	UKBB	2017
rs11209331	C	T	Comparative height size at age 10	Neale B	UKBB	2017
rs11209331	C	T	Forced expiratory volume in 1-second	Neale B	UKBB	2017
rs11209331	C	T	Forced expiratory volume in 1-second, best measure	Neale B	UKBB	2017
rs11209331	C	T	Forced expiratory volume in 1-second, predicted	Neale B	UKBB	2017
rs11209331	C	T	Forced vital capacity	Neale B	UKBB	2017
rs11209331	C	T	Forced vital capacity, best measure	Neale B	UKBB	2017
rs11209331	C	T	Height	Neale B	UKBB	2017
rs11209331	C	T	Leg fat-free mass left	Neale B	UKBB	2017
rs11209331	C	T	Leg fat-free mass right	Neale B	UKBB	2017
rs11209331	C	T	Leg predicted mass left	Neale B	UKBB	2017
rs11209331	C	T	Leg predicted mass right	Neale B	UKBB	2017
rs11209331	C	T	Sitting height	Neale B	UKBB	2017
rs11209331	C	T	Trunk fat-free mass	Neale B	UKBB	2017
rs11209331	C	T	Trunk predicted mass	Neale B	UKBB	2017
rs11209331	C	T	Weight	Neale B	UKBB	2017
rs11209331	C	T	Whole body fat-free mass	Neale B	UKBB	2017
rs11209331	C	T	Whole body water mass	Neale B	UKBB	2017
rs11210871	C	G	Intelligence multi trait analysis	Hill WD	29326435	2018
rs11210871	C	G	Age at menarche	Neale B	UKBB	2017
rs11210871	C	G	Current tobacco smoking	Neale B	UKBB	2017
rs11210871	C	G	Qualifications: A levels or as levels or equivalent	Neale B	UKBB	2017
rs11210871	C	G	Qualifications: college or university degree	Neale B	UKBB	2017
rs11210871	C	G	Qualifications: none	Neale B	UKBB	2017
rs11210871	C	G	Smoking status: current	Neale B	UKBB	2017
rs11210871	C	G	Age at menarche	ReproGen	25231870	2014
rs11210871	C	G	Years of educational attainment	SSGAC	27225129	2016
rs11240695	A	C	Age at menarche	Neale B	UKBB	2017
rs1131017	C	G	Eosinophil count	Astle W	27863252	2016
rs1131017	C	G	Eosinophil percentage of granulocytes	Astle W	27863252	2016

rs1131017	C	G	Eosinophil percentage of white cells	Astle W	27863252	2016
rs1131017	C	G	Neutrophil percentage of granulocytes	Astle W	27863252	2016
rs1131017	C	G	Sum eosinophil basophil counts	Astle W	27863252	2016
rs1131017	C	G	Allergic disease	Ferreira M	29083406	2017
rs1131017	C	G	Inflammatory skin disease	Baurecht H	25574825	2015
rs1131017	C	G	Arm fat-free mass left	Neale B	UKBB	2017
rs1131017	C	G	Arm fat-free mass right	Neale B	UKBB	2017
rs1131017	C	G	Arm predicted mass left	Neale B	UKBB	2017
rs1131017	C	G	Arm predicted mass right	Neale B	UKBB	2017
rs1131017	C	G	Asthma	Neale B	UKBB	2017
rs1131017	C	G	Basal metabolic rate	Neale B	UKBB	2017
rs1131017	C	G	Body mass index	Neale B	UKBB	2017
rs1131017	C	G	Forced expiratory volume in 1-second, best measure	Neale B	UKBB	2017
rs1131017	C	G	Hayfever, allergic rhinitis or eczema	Neale B	UKBB	2017
rs1131017	C	G	Impedance of arm left	Neale B	UKBB	2017
rs1131017	C	G	Impedance of arm right	Neale B	UKBB	2017
rs1131017	C	G	Impedance of leg left	Neale B	UKBB	2017
rs1131017	C	G	Impedance of leg right	Neale B	UKBB	2017
rs1131017	C	G	Impedance of whole body	Neale B	UKBB	2017
rs1131017	C	G	Leg fat-free mass left	Neale B	UKBB	2017
rs1131017	C	G	Leg fat-free mass right	Neale B	UKBB	2017
rs1131017	C	G	Leg predicted mass left	Neale B	UKBB	2017
rs1131017	C	G	Leg predicted mass right	Neale B	UKBB	2017
rs1131017	C	G	No blood clot, bronchitis, emphysema, asthma, rhinitis, eczema or allergy diagnosed by doctor	Neale B	UKBB	2017
rs1131017	C	G	Qualifications: college or university degree	Neale B	UKBB	2017
rs1131017	C	G	Qualifications: none	Neale B	UKBB	2017
rs1131017	C	G	Self-reported asthma	Neale B	UKBB	2017
rs1131017	C	G	Self-reported hypothyroidism or myxoedema	Neale B	UKBB	2017
rs1131017	C	G	Treatment with levothyroxine sodium	Neale B	UKBB	2017
rs1131017	C	G	Trunk fat-free mass	Neale B	UKBB	2017
rs1131017	C	G	Trunk predicted mass	Neale B	UKBB	2017
rs1131017	C	G	Whole body fat-free mass	Neale B	UKBB	2017
rs1131017	C	G	Whole body water mass	Neale B	UKBB	2017
rs1131017	C	G	Rheumatoid arthritis	Okada Y	24390342	2014
rs1131017	C	G	Years of educational attainment	SSGAC	27225129	2016
rs113388806	A	T	Height	GIANT	28146470	2017
rs113388806	A	T	Height	GIANT	28146470	2017
rs115435316	A	G	Age at menarche	Neale B	UKBB	2017
rs115435316	A	G	Arm fat-free mass right	Neale B	UKBB	2017
rs115435316	A	G	Arm predicted mass right	Neale B	UKBB	2017
rs115435316	A	G	Basal metabolic rate	Neale B	UKBB	2017
rs115435316	A	G	Height	Neale B	UKBB	2017
rs115435316	A	G	Sitting height	Neale B	UKBB	2017
rs115435316	A	G	Trunk fat-free mass	Neale B	UKBB	2017

rs115435316	A	G	Trunk predicted mass	Neale B	UKBB	2017
rs115435316	A	G	Whole body fat-free mass	Neale B	UKBB	2017
rs115435316	A	G	Whole body water mass	Neale B	UKBB	2017
rs117143374	C	T	Platelet count	Astle W	27863252	2016
rs117143374	C	T	Plateletcrit	Astle W	27863252	2016
rs117143374	C	T	Age at menarche	Neale B	UKBB	2017
rs117143374	C	T	Morning or evening person	Neale B	UKBB	2017
rs1172955	T	A	Age at menarche	Neale B	UKBB	2017
rs11767400	A	C	Menarche age at onset	ReproGen	25231870	2014
rs11767400	A	C	Age at menarche	ReproGen	25231870	2014
rs11786868	C	G	Birth weight of first child	Neale B	UKBB	2017
rs11786868	C	G	Hair or balding pattern: pattern 4	Neale B	UKBB	2017
rs11786868	C	G	Systolic blood pressure	Neale B	UKBB	2017
rs11873906	A	G	Age at menarche	Neale B	UKBB	2017
rs11873906	A	G	Impedance of arm left	Neale B	UKBB	2017
rs11873906	A	G	Impedance of arm right	Neale B	UKBB	2017
rs11873906	A	G	Impedance of whole body	Neale B	UKBB	2017
rs12460047	A	G	Arm fat mass left	Neale B	UKBB	2017
rs12460047	A	G	Arm fat mass right	Neale B	UKBB	2017
rs12460047	A	G	Arm fat percentage left	Neale B	UKBB	2017
rs12460047	A	G	Arm fat percentage right	Neale B	UKBB	2017
rs12460047	A	G	Body fat percentage	Neale B	UKBB	2017
rs12460047	A	G	Leg fat mass left	Neale B	UKBB	2017
rs12460047	A	G	Leg fat mass right	Neale B	UKBB	2017
rs12460047	A	G	Leg fat percentage left	Neale B	UKBB	2017
rs12460047	A	G	Leg fat percentage right	Neale B	UKBB	2017
rs12460047	A	G	Qualifications: college or university degree	Neale B	UKBB	2017
rs12460047	A	G	Trunk fat mass	Neale B	UKBB	2017
rs12460047	A	G	Trunk fat percentage	Neale B	UKBB	2017
rs12460047	A	G	Waist circumference	Neale B	UKBB	2017
rs12460047	A	G	Whole body fat mass	Neale B	UKBB	2017
rs12571664	C	T	Menarche age at onset	ReproGen	25231870	2014
rs12571664	C	T	Age at menarche	ReproGen	25231870	2014
rs12603280	A	G	Age at menarche	ReproGen	25231870	2014
rs12663002	C	T	Mean corpuscular hemoglobin	Astle W	27863252	2016
rs12663002	C	T	Mean corpuscular volume	Astle W	27863252	2016
rs12663002	C	T	Sitting height	Neale B	UKBB	2017
rs12894936	C	T	Menarche age at onset	Pickrell JK	27182965	2016
rs12894936	C	T	Age at menarche	Neale B	UKBB	2017
rs12915845	C	T	Menarche age at onset	ReproGen	25231870	2014
rs12915845	C	T	Age at menarche	Neale B	UKBB	2017
rs12915845	C	T	Height	Neale B	UKBB	2017
rs12915845	C	T	Relative age of first facial hair	Neale B	UKBB	2017
rs12915845	C	T	Age at menarche	ReproGen	25231870	2014
rs13043968	A	C	Comparative height size at age 10	Neale B	UKBB	2017

rs13173441	C	T	Mean platelet volume	Astle W	27863252	2016
rs13173441	C	T	Platelet distribution width	Astle W	27863252	2016
rs13322435	A	G	Birthweight	EGGC	23202124	2013
rs13322435	A	G	Waist circumference adjusted for BMI	GIANT	25673412	2015
rs13322435	A	G	Birth weight	EGGC	23202124	2013
rs13322435	A	G	Birth weight	Horikoshi M	27680694	2016
rs13322435	A	G	Birth weight	Neale B	UKBB	2017
rs13322435	A	G	Heel bone mineral density	Neale B	UKBB	2017
rs13322435	A	G	Heel bone mineral density left	Neale B	UKBB	2017
rs13322435	A	G	Sitting height	Neale B	UKBB	2017
rs13322435	A	G	Waist circumference	Neale B	UKBB	2017
rs13322435	A	G	Age at menarche	ReproGen	25231870	2014
rs1414186	T	G	Age at menarche	Neale B	UKBB	2017
rs141847393	C	T	Granulocyte percentage of myeloid white cells	Astle W	27863252	2016
rs141847393	C	T	Forced vital capacity	Neale B	UKBB	2017
rs142058842	C	G	Age at voice drop	Pickrell JK	27182965	2016
rs142058842	C	G	Age at menarche	Neale B	UKBB	2017
rs142058842	C	G	Height	Neale B	UKBB	2017
rs142058842	C	G	Relative age of first facial hair	Neale B	UKBB	2017
rs142058842	C	G	Relative age voice broke	Neale B	UKBB	2017
rs1428120	T	G	Arm fat mass left	Neale B	UKBB	2017
rs1428120	T	G	Arm fat mass right	Neale B	UKBB	2017
rs1428120	T	G	Arm fat percentage left	Neale B	UKBB	2017
rs1428120	T	G	Arm fat percentage right	Neale B	UKBB	2017
rs1428120	T	G	Body fat percentage	Neale B	UKBB	2017
rs1428120	T	G	Body mass index	Neale B	UKBB	2017
rs1428120	T	G	Comparative body size at age 10	Neale B	UKBB	2017
rs1428120	T	G	Hip circumference	Neale B	UKBB	2017
rs1428120	T	G	Leg fat mass left	Neale B	UKBB	2017
rs1428120	T	G	Leg fat mass right	Neale B	UKBB	2017
rs1428120	T	G	Leg fat percentage left	Neale B	UKBB	2017
rs1428120	T	G	Leg fat percentage right	Neale B	UKBB	2017
rs1428120	T	G	Trunk fat mass	Neale B	UKBB	2017
rs1428120	T	G	Trunk fat percentage	Neale B	UKBB	2017
rs1428120	T	G	Waist circumference	Neale B	UKBB	2017
rs1428120	T	G	Weight	Neale B	UKBB	2017
rs1428120	T	G	Whole body fat mass	Neale B	UKBB	2017
rs1435753	C	T	Arm fat percentage left	Neale B	UKBB	2017
rs1435753	C	T	Arm fat percentage right	Neale B	UKBB	2017
rs1470750	G	C	Arm fat-free mass left	Neale B	UKBB	2017
rs1470750	G	C	Arm fat-free mass right	Neale B	UKBB	2017
rs1470750	G	C	Arm predicted mass left	Neale B	UKBB	2017
rs1470750	G	C	Arm predicted mass right	Neale B	UKBB	2017
rs1470750	G	C	Basal metabolic rate	Neale B	UKBB	2017
rs1470750	G	C	Hip circumference	Neale B	UKBB	2017

rs1470750	G	C	Leg fat-free mass left	Neale B	UKBB	2017
rs1470750	G	C	Leg fat-free mass right	Neale B	UKBB	2017
rs1470750	G	C	Leg predicted mass left	Neale B	UKBB	2017
rs1470750	G	C	Leg predicted mass right	Neale B	UKBB	2017
rs1470750	G	C	Morning or evening person	Neale B	UKBB	2017
rs1470750	G	C	Trunk fat mass	Neale B	UKBB	2017
rs1470750	G	C	Trunk fat-free mass	Neale B	UKBB	2017
rs1470750	G	C	Trunk predicted mass	Neale B	UKBB	2017
rs1470750	G	C	Waist circumference	Neale B	UKBB	2017
rs1470750	G	C	Weight	Neale B	UKBB	2017
rs1470750	G	C	Whole body fat-free mass	Neale B	UKBB	2017
rs1470750	G	C	Whole body water mass	Neale B	UKBB	2017
rs150821390	C	T	Comparative height size at age 10	Neale B	UKBB	2017
rs150821390	C	T	Height	Neale B	UKBB	2017
rs150821390	C	T	Sitting height	Neale B	UKBB	2017
rs1512238	G	A	Age at menarche	Neale B	UKBB	2017
rs1512238	G	A	Nap during day	Neale B	UKBB	2017
rs153793	G	A	Dihomo-gamma-linolenic acid	Guan W	24823311	2014
rs157877	A	G	Age at menarche	Neale B	UKBB	2017
rs16841867	C	G	Height	GIANT	25282103	2014
rs16841867	C	G	Height	Neale B	UKBB	2017
rs16841867	C	G	Self-reported high cholesterol	Neale B	UKBB	2017
rs16917237	G	T	Body mass index females	Akiyama M	28892062	2017
rs16917237	G	T	Body mass index males	Akiyama M	28892062	2017
rs16917237	G	T	Body mass index	Akiyama M	28892062	2017
rs16917237	G	T	Body mass index in physically active females	GIANT	28448500	2017
rs16917237	G	T	Body mass index in physically active indivdiuals	GIANT	28448500	2017
rs16917237	G	T	Body mass index in physically active indivdiuals	GIANT	28448500	2017
rs16917237	G	T	Body mass index in females greater than 50 years of age	GIANT	26426971	2015
rs16917237	G	T	Body mass index in females	GIANT	25673413	2015
rs16917237	G	T	Body mass index in physically inactive indivdiuals	GIANT	28448500	2017
rs16917237	G	T	Body mass index in males greater than 50 years of age	GIANT	26426971	2015
rs16917237	G	T	Body mass index in males	GIANT	25673413	2015
rs16917237	G	T	Body mass index in female non-smokers	GIANT	28443625	2017
rs16917237	G	T	Body mass index in non-smokers	GIANT	28443625	2017
rs16917237	G	T	Body mass index in non-smokers	GIANT	28443625	2017
rs16917237	G	T	Body mass index in smokers	GIANT	28443625	2017
rs16917237	G	T	Body mass index in smokers	GIANT	28443625	2017
rs16917237	G	T	Body mass index ajusted for physical activity in females	GIANT	28448500	2017
rs16917237	G	T	Body mass index adjsuted for physical activity in males	GIANT	28448500	2017
rs16917237	G	T	Body mass index adjusted for physical activity	GIANT	28448500	2017
rs16917237	G	T	Body mass index adjusted for physical activity	GIANT	28448500	2017
rs16917237	G	T	Body mass index adjusted for smoking in females	GIANT	28443625	2017
rs16917237	G	T	Body mass index adjusted for smoking in males	GIANT	28443625	2017
rs16917237	G	T	Body mass index adjusted for smoking	GIANT	28443625	2017

rs16917237	G	T	Body mass index adjusted for smoking	GIANT	28443625	2017
rs16917237	G	T	Body mass index	Speliotes EK	20935630	2010
rs16917237	G	T	Body mass index	GIANT	23754948	2013
rs16917237	G	T	Body mass index	GIANT	25673413	2015
rs16917237	G	T	Body mass index	GIANT	25673413	2015
rs16917237	G	T	Hip circumference in females	GIANT	25673412	2015
rs16917237	G	T	Hip circumference	GIANT	25673412	2015
rs16917237	G	T	Hip circumference	GIANT	25673412	2015
rs16917237	G	T	Obesity class 1	GIANT	23563607	2013
rs16917237	G	T	Overweight	GIANT	23563607	2013
rs16917237	G	T	Waist circumference in females	GIANT	25673412	2015
rs16917237	G	T	Waist circumference in males	GIANT	25673412	2015
rs16917237	G	T	Waist circumference	GIANT	25673412	2015
rs16917237	G	T	Waist circumference	GIANT	25673412	2015
rs16917237	G	T	Weight	GIANT	23754948	2013
rs16917237	G	T	Body mass index	Speliotes EK	20935630	2010
rs16917237	G	T	Age at menarche	Neale B	UKBB	2017
rs16917237	G	T	Arm fat mass left	Neale B	UKBB	2017
rs16917237	G	T	Arm fat mass right	Neale B	UKBB	2017
rs16917237	G	T	Arm fat percentage left	Neale B	UKBB	2017
rs16917237	G	T	Arm fat percentage right	Neale B	UKBB	2017
rs16917237	G	T	Arm fat-free mass left	Neale B	UKBB	2017
rs16917237	G	T	Arm fat-free mass right	Neale B	UKBB	2017
rs16917237	G	T	Arm predicted mass left	Neale B	UKBB	2017
rs16917237	G	T	Arm predicted mass right	Neale B	UKBB	2017
rs16917237	G	T	Basal metabolic rate	Neale B	UKBB	2017
rs16917237	G	T	Body fat percentage	Neale B	UKBB	2017
rs16917237	G	T	Body mass index	Neale B	UKBB	2017
rs16917237	G	T	Hip circumference	Neale B	UKBB	2017
rs16917237	G	T	Impedance of arm left	Neale B	UKBB	2017
rs16917237	G	T	Impedance of arm right	Neale B	UKBB	2017
rs16917237	G	T	Impedance of leg left	Neale B	UKBB	2017
rs16917237	G	T	Impedance of leg right	Neale B	UKBB	2017
rs16917237	G	T	Impedance of whole body	Neale B	UKBB	2017
rs16917237	G	T	Leg fat mass left	Neale B	UKBB	2017
rs16917237	G	T	Leg fat mass right	Neale B	UKBB	2017
rs16917237	G	T	Leg fat percentage left	Neale B	UKBB	2017
rs16917237	G	T	Leg fat percentage right	Neale B	UKBB	2017
rs16917237	G	T	Leg fat-free mass left	Neale B	UKBB	2017
rs16917237	G	T	Leg fat-free mass right	Neale B	UKBB	2017
rs16917237	G	T	Leg predicted mass left	Neale B	UKBB	2017
rs16917237	G	T	Leg predicted mass right	Neale B	UKBB	2017
rs16917237	G	T	Past tobacco smoking	Neale B	UKBB	2017
rs16917237	G	T	Trunk fat mass	Neale B	UKBB	2017
rs16917237	G	T	Trunk fat percentage	Neale B	UKBB	2017
rs16917237	G	T	Trunk fat-free mass	Neale B	UKBB	2017
rs16917237	G	T	Trunk predicted mass	Neale B	UKBB	2017

rs16917237	G	T	Waist circumference	Neale B	UKBB	2017
rs16917237	G	T	Weight	Neale B	UKBB	2017
rs16917237	G	T	Whole body fat mass	Neale B	UKBB	2017
rs16917237	G	T	Whole body fat-free mass	Neale B	UKBB	2017
rs16917237	G	T	Whole body water mass	Neale B	UKBB	2017
rs16917237	G	T	Age at menarche	ReproGen	25231870	2014
rs16918378	C	T	Age at menarche	ReproGen	25231870	2014
rs16937956	A	G	Body mass index	Akiyama M	28892062	2017
rs16937956	A	G	Body mass index	Akiyama M	28892062	2017
rs16937956	A	G	Age at menarche	Neale B	UKBB	2017
rs16937956	A	G	Leg fat percentage left	Neale B	UKBB	2017
rs16937956	A	G	Leg fat percentage right	Neale B	UKBB	2017
rs16937956	A	G	Age at menarche	ReproGen	25231870	2014
rs17035311	A	C	Age at menarche	Neale B	UKBB	2017
rs17035311	A	C	Impedance of leg right	Neale B	UKBB	2017
rs1704528	T	C	Menarche age at onset	Pickrell JK	27182965	2016
rs1704528	T	C	Age at menarche	Neale B	UKBB	2017
rs1704528	T	C	Arm fat-free mass left	Neale B	UKBB	2017
rs1704528	T	C	Arm fat-free mass right	Neale B	UKBB	2017
rs1704528	T	C	Arm predicted mass left	Neale B	UKBB	2017
rs1704528	T	C	Arm predicted mass right	Neale B	UKBB	2017
rs1704528	T	C	Hair or balding pattern: pattern 4	Neale B	UKBB	2017
rs1704528	T	C	Height	Neale B	UKBB	2017
rs1704528	T	C	Relative age of first facial hair	Neale B	UKBB	2017
rs1704528	T	C	Relative age voice broke	Neale B	UKBB	2017
rs1704528	T	C	Trunk fat-free mass	Neale B	UKBB	2017
rs1704528	T	C	Trunk predicted mass	Neale B	UKBB	2017
rs1704528	T	C	Whole body fat-free mass	Neale B	UKBB	2017
rs1704528	T	C	Whole body water mass	Neale B	UKBB	2017
rs17085593	C	G	Height	GIANT	25282103	2014
rs17085593	C	G	log Proinsulin	MAGIC	21873549	2011
rs17085593	C	G	Arm fat-free mass left	Neale B	UKBB	2017
rs17085593	C	G	Arm fat-free mass right	Neale B	UKBB	2017
rs17085593	C	G	Arm predicted mass left	Neale B	UKBB	2017
rs17085593	C	G	Arm predicted mass right	Neale B	UKBB	2017
rs17085593	C	G	Basal metabolic rate	Neale B	UKBB	2017
rs17085593	C	G	Comparative height size at age 10	Neale B	UKBB	2017
rs17085593	C	G	Height	Neale B	UKBB	2017
rs17085593	C	G	Leg fat-free mass left	Neale B	UKBB	2017
rs17085593	C	G	Leg fat-free mass right	Neale B	UKBB	2017
rs17085593	C	G	Leg predicted mass left	Neale B	UKBB	2017
rs17085593	C	G	Leg predicted mass right	Neale B	UKBB	2017
rs17085593	C	G	Trunk fat-free mass	Neale B	UKBB	2017
rs17085593	C	G	Trunk predicted mass	Neale B	UKBB	2017
rs17085593	C	G	Weight	Neale B	UKBB	2017
rs17085593	C	G	Whole body fat-free mass	Neale B	UKBB	2017
rs17085593	C	G	Whole body water mass	Neale B	UKBB	2017
rs17390720	C	G	Height	Neale B	UKBB	2017

rs1984870	T	G	Age at menarche	Neale B	UKBB	2017
rs1984870	T	G	Age at menarche	ReproGen	25231870	2014
rs2066323	G	A	High light scatter percentage of red cells	Astle W	27863252	2016
rs2066323	G	A	High light scatter reticulocyte count	Astle W	27863252	2016
rs2066323	G	A	Immature fraction of reticulocytes	Astle W	27863252	2016
rs2066323	G	A	Mean corpuscular hemoglobin	Astle W	27863252	2016
rs2066323	G	A	Mean corpuscular volume	Astle W	27863252	2016
rs2066323	G	A	Reticulocyte count	Astle W	27863252	2016
rs2066323	G	A	Reticulocyte fraction of red cells	Astle W	27863252	2016
rs2066323	G	A	Arm fat-free mass left	Neale B	UKBB	2017
rs2066323	G	A	Arm fat-free mass right	Neale B	UKBB	2017
rs2066323	G	A	Arm predicted mass left	Neale B	UKBB	2017
rs2066323	G	A	Arm predicted mass right	Neale B	UKBB	2017
rs2066323	G	A	Basal metabolic rate	Neale B	UKBB	2017
rs2066323	G	A	Birth weight	Neale B	UKBB	2017
rs2066323	G	A	Ever smoked	Neale B	UKBB	2017
rs2066323	G	A	Height	Neale B	UKBB	2017
rs2066323	G	A	Leg fat-free mass left	Neale B	UKBB	2017
rs2066323	G	A	Leg fat-free mass right	Neale B	UKBB	2017
rs2066323	G	A	Leg predicted mass left	Neale B	UKBB	2017
rs2066323	G	A	Leg predicted mass right	Neale B	UKBB	2017
rs2066323	G	A	Nervous feelings	Neale B	UKBB	2017
rs2066323	G	A	Past tobacco smoking	Neale B	UKBB	2017
rs2066323	G	A	Trunk fat-free mass	Neale B	UKBB	2017
rs2066323	G	A	Trunk predicted mass	Neale B	UKBB	2017
rs2066323	G	A	Weight	Neale B	UKBB	2017
rs2066323	G	A	Whole body fat-free mass	Neale B	UKBB	2017
rs2066323	G	A	Whole body water mass	Neale B	UKBB	2017
rs2066323	G	A	Worrier or anxious feelings	Neale B	UKBB	2017
rs2066323	G	A	Schizophrenia	PGC	25056061	2014
rs2108753	T	C	Mean platelet volume	Astle W	27863252	2016
rs2108753	T	C	Age at menarche	Neale B	UKBB	2017
rs2108753	T	C	Qualifications: college or university degree	Neale B	UKBB	2017
rs2267812	C	A	Red cell distribution width	Astle W	27863252	2016
rs2267812	C	A	Arm fat percentage left	Neale B	UKBB	2017
rs2267812	C	A	Arm fat percentage right	Neale B	UKBB	2017
rs2267812	C	A	Body mass index	Neale B	UKBB	2017
rs2267812	C	A	Self-reported hypertension	Neale B	UKBB	2017
rs2267812	C	A	Vascular or heart problems diagnosed by doctor: high blood pressure	Neale B	UKBB	2017
rs2267812	C	A	Vascular or heart problems diagnosed by doctor: none of the above	Neale B	UKBB	2017
rs2267812	C	A	Age at menarche	ReproGen	25231870	2014
rs2271758	G	T	Basal metabolic rate	Neale B	UKBB	2017
rs2271758	G	T	Hip circumference	Neale B	UKBB	2017
rs2271758	G	T	Leg fat-free mass left	Neale B	UKBB	2017
rs2271758	G	T	Leg predicted mass left	Neale B	UKBB	2017
rs2271758	G	T	Weight	Neale B	UKBB	2017
rs2271758	G	T	Whole body fat mass	Neale B	UKBB	2017

rs2295094	A	G	Arm fat-free mass left	Neale B	UKBB	2017
rs2295094	A	G	Arm fat-free mass right	Neale B	UKBB	2017
rs2295094	A	G	Arm predicted mass left	Neale B	UKBB	2017
rs2295094	A	G	Arm predicted mass right	Neale B	UKBB	2017
rs2295094	A	G	Basal metabolic rate	Neale B	UKBB	2017
rs2295094	A	G	Comparative height size at age 10	Neale B	UKBB	2017
rs2295094	A	G	Height	Neale B	UKBB	2017
rs2295094	A	G	Impedance of arm right	Neale B	UKBB	2017
rs2295094	A	G	Impedance of whole body	Neale B	UKBB	2017
rs2295094	A	G	Irritability	Neale B	UKBB	2017
rs2295094	A	G	Leg fat-free mass left	Neale B	UKBB	2017
rs2295094	A	G	Leg fat-free mass right	Neale B	UKBB	2017
rs2295094	A	G	Leg predicted mass left	Neale B	UKBB	2017
rs2295094	A	G	Leg predicted mass right	Neale B	UKBB	2017
rs2295094	A	G	Other malignant neoplasms of skin	Neale B	UKBB	2017
rs2295094	A	G	Sitting height	Neale B	UKBB	2017
rs2295094	A	G	Trunk fat-free mass	Neale B	UKBB	2017
rs2295094	A	G	Trunk predicted mass	Neale B	UKBB	2017
rs2295094	A	G	Weight	Neale B	UKBB	2017
rs2295094	A	G	Whole body fat-free mass	Neale B	UKBB	2017
rs2295094	A	G	Whole body water mass	Neale B	UKBB	2017
rs2300922	C	T	Height	GIANT	25282103	2014
rs2300922	C	T	Age at menarche	Neale B	UKBB	2017
rs2300922	C	T	Height	Neale B	UKBB	2017
rs2300922	C	T	Age at menarche	ReproGen	25231870	2014
rs2312205	A	G	Comparative height size at age 10	Neale B	UKBB	2017
rs2312205	A	G	Height	Neale B	UKBB	2017
rs2312205	A	G	Weight	Neale B	UKBB	2017
rs2461794	G	A	Age at menarche	Neale B	UKBB	2017
rs2542420	G	C	Age at menarche	Neale B	UKBB	2017
rs2548458	C	T	Mean platelet volume	Astle W	27863252	2016
rs2548458	C	T	Crohns disease	IBDGC	26192919	2015
rs2548458	C	T	Alkaline phosphatase	Prins B	28887542	2017
rs2659007	G	A	IgG fucosylation	Shen X	28878392	2017
rs2659007	G	A	IgG galactosylation	Shen X	28878392	2017
rs2659007	G	A	IgG monogalactosylation	Shen X	28878392	2017
rs2679894	G	A	Menarche age at onset	Pickrell JK	27182965	2016
rs2679894	G	A	Age at menarche	Neale B	UKBB	2017
rs2724961	C	T	Age at menarche	Neale B	UKBB	2017
rs2724961	C	T	Age at menarche	ReproGen	25231870	2014
rs2787487	G	C	Age at menarche	Neale B	UKBB	2017
rs2787487	G	C	Age at menarche	ReproGen	25231870	2014
rs29941	G	A	Body mass index in physically active indivdiuals	GIANT	28448500	2017
rs29941	G	A	Body mass index in physically active indivdiuals	GIANT	28448500	2017
rs29941	G	A	Body mass index adjusted for physical activity	GIANT	28448500	2017
rs29941	G	A	Body mass index adjusted for physical activity	GIANT	28448500	2017
rs29941	G	A	Body mass index adjusted for smoking	GIANT	28443625	2017

rs29941	G	A	Body mass index	Speliotes EK	20935630	2010
rs29941	G	A	Body mass index	GIANT	23754948	2013
rs29941	G	A	Body mass index	GIANT	25673413	2015
rs29941	G	A	Body mass index	GIANT	29273807	2018
rs29941	G	A	Body mass index	GIANT	25673413	2015
rs29941	G	A	Body mass index	GIANT	29273807	2018
rs29941	G	A	Weight	GIANT	23754948	2013
rs29941	G	A	Body mass index	Thorleifsson G	19079260	2008
rs29941	G	A	Body mass index	Speliotes EK	20935630	2010
rs29941	G	A	Body mass index	Guo	23001569	2012
rs29941	G	A	Extreme obesity with early age of onset	Wheeler E	23563609	2013
rs29941	G	A	Weight	Thorleifsson G	19079260	2008
rs29941	G	A	BMI adjusted for smoking behaviour	GIANT	28443625	2017
rs29941	G	A	Body mass index	Thorleifsson G	19079260	2008
rs29941	G	A	Body mass index	Speliotes EK	20935630	2010
rs29941	G	A	Body mass index	GIANT	25673413	2015
rs29941	G	A	Body mass index	Akiyama M	28892062	2017
rs29941	G	A	Body mass index joint analysis main effects and smoking interaction	GIANT	28443625	2017
rs29941	G	A	Weight	Thorleifsson G	19079260	2008
rs29941	G	A	Arm fat-free mass left	Neale B	UKBB	2017
rs29941	G	A	Arm fat-free mass right	Neale B	UKBB	2017
rs29941	G	A	Arm predicted mass left	Neale B	UKBB	2017
rs29941	G	A	Arm predicted mass right	Neale B	UKBB	2017
rs29941	G	A	Basal metabolic rate	Neale B	UKBB	2017
rs29941	G	A	Impedance of arm left	Neale B	UKBB	2017
rs29941	G	A	Impedance of arm right	Neale B	UKBB	2017
rs29941	G	A	Impedance of leg left	Neale B	UKBB	2017
rs29941	G	A	Impedance of leg right	Neale B	UKBB	2017
rs29941	G	A	Impedance of whole body	Neale B	UKBB	2017
rs29941	G	A	Leg fat-free mass left	Neale B	UKBB	2017
rs29941	G	A	Leg fat-free mass right	Neale B	UKBB	2017
rs29941	G	A	Leg predicted mass left	Neale B	UKBB	2017
rs29941	G	A	Leg predicted mass right	Neale B	UKBB	2017
rs29941	G	A	Trunk fat-free mass	Neale B	UKBB	2017
rs29941	G	A	Trunk predicted mass	Neale B	UKBB	2017
rs29941	G	A	Weight	Neale B	UKBB	2017
rs29941	G	A	Whole body fat-free mass	Neale B	UKBB	2017
rs29941	G	A	Whole body water mass	Neale B	UKBB	2017
rs29941	G	A	Body mass index	Thorleifsson G	19079260	2008
rs29941	G	A	Body mass index	Speliotes EK	20935630	2010
rs29941	G	A	Body weight	Thorleifsson G	19079260	2008
rs3113862	A	G	Age at menarche	Neale B	UKBB	2017
rs3113862	A	G	Relative age of first facial hair	Neale B	UKBB	2017
rs35935052	G	T	Arm fat-free mass left	Neale B	UKBB	2017

rs35935052	G	T	Arm predicted mass left	Neale B	UKBB	2017
rs35935052	G	T	Arm predicted mass right	Neale B	UKBB	2017
rs35935052	G	T	Basal metabolic rate	Neale B	UKBB	2017
rs35935052	G	T	Comparative body size at age 10	Neale B	UKBB	2017
rs35935052	G	T	Leg fat-free mass left	Neale B	UKBB	2017
rs35935052	G	T	Leg fat-free mass right	Neale B	UKBB	2017
rs35935052	G	T	Leg predicted mass left	Neale B	UKBB	2017
rs35935052	G	T	Leg predicted mass right	Neale B	UKBB	2017
rs35935052	G	T	Weight	Neale B	UKBB	2017
rs35935052	G	T	Whole body fat-free mass	Neale B	UKBB	2017
rs35935052	G	T	Whole body water mass	Neale B	UKBB	2017
rs36093651	C	T	Age at menarche	Neale B	UKBB	2017
rs3733632	G	A	Age at menarche	Neale B	UKBB	2017
rs3733632	G	A	Age at menarche	ReproGen	25231870	2014
rs3746619	A	C	Relative age of first facial hair	Neale B	UKBB	2017
rs3764002	C	T	Arm fat percentage left	Neale B	UKBB	2017
rs3764002	C	T	Arm fat percentage right	Neale B	UKBB	2017
rs3764002	C	T	Body fat percentage	Neale B	UKBB	2017
rs3764002	C	T	Impedance of leg left	Neale B	UKBB	2017
rs3764002	C	T	Impedance of leg right	Neale B	UKBB	2017
rs3764002	C	T	Impedance of whole body	Neale B	UKBB	2017
rs3764002	C	T	Leg fat mass left	Neale B	UKBB	2017
rs3764002	C	T	Leg fat mass right	Neale B	UKBB	2017
rs3764002	C	T	Leg fat percentage left	Neale B	UKBB	2017
rs3764002	C	T	Leg fat percentage right	Neale B	UKBB	2017
rs3764002	C	T	Sitting height	Neale B	UKBB	2017
rs3764002	C	T	Trunk fat mass	Neale B	UKBB	2017
rs3764002	C	T	Trunk fat percentage	Neale B	UKBB	2017
rs3764002	C	T	Waist circumference	Neale B	UKBB	2017
rs3764002	C	T	Whole body fat mass	Neale B	UKBB	2017
rs395962	G	T	Standardized difference in height between age 14 years and adult	EGGC	23449627	2013
rs395962	G	T	Tanner stage	EGGC	24770850	2014
rs395962	G	T	Height in females	GIANT	23754948	2013
rs395962	G	T	Height in males	GIANT	23754948	2013
rs395962	G	T	Height tails	GIANT	23563607	2013
rs395962	G	T	Height	GIANT	20881960	2010
rs395962	G	T	Height	GIANT	23754948	2013
rs395962	G	T	Height	GIANT	25282103	2014
rs395962	G	T	Hip circumference adjusted for BMI	GIANT	25673412	2015
rs395962	G	T	Waist circumference adjusted for BMI	GIANT	25673412	2015
rs395962	G	T	Waist circumference in physically active females	GIANT	28448500	2017
rs395962	G	T	Waist circumeference in physically active males	GIANT	28448500	2017
rs395962	G	T	Waist circumference in physically active indivdiuals	GIANT	28448500	2017
rs395962	G	T	Waist circumference in physically active indivdiuals	GIANT	28448500	2017
rs395962	G	T	Waist circumference in male non-smokers	GIANT	28443625	2017
rs395962	G	T	Waist circumference in non-smokers	GIANT	28443625	2017
rs395962	G	T	Waist circumference in non-smokers	GIANT	28443625	2017

rs395962	G	T	Waist circumference adjusted for physical activity in females	GIANT	28448500	2017
rs395962	G	T	Waist circumeference adjusted for physical activity in males	GIANT	28448500	2017
rs395962	G	T	Waist circumference adjusted for physical activity	GIANT	28448500	2017
rs395962	G	T	Waist circumference adjusted for physical activity	GIANT	28448500	2017
rs395962	G	T	Waist cirumference adjusted for smoking in males	GIANT	28443625	2017
rs395962	G	T	Waist circumference adjusted for smoking	GIANT	28443625	2017
rs395962	G	T	Waist circumference adjusted for smoking	GIANT	28443625	2017
rs395962	G	T	Age at menarche	Perry JR	19448620	2009
rs395962	G	T	Height	GIANT	20881960	2010
rs395962	G	T	Waist circumference adjusted for BMI adjusted for smoking behaviour	GIANT	28443625	2017
rs395962	G	T	Waist circumference adjusted for BMI adjusted for smoking behaviour	GIANT	28443625	2017
rs395962	G	T	Waist circumference adjusted for BMI in active individuals	GIANT	28448500	2017
rs395962	G	T	Waist circumference adjusted for BMI in non smokers	GIANT	28443625	2017
rs395962	G	T	Waist circumference adjusted for BMI in non smokers	GIANT	28443625	2017
rs395962	G	T	Waist circumference adjusted for BMI joint analysis main effects and smoking interaction	GIANT	28443625	2017
rs395962	G	T	Waist circumference adjusted for BMI joint analysis main effects and smoking interaction	GIANT	28443625	2017
rs395962	G	T	Age at menarche	Neale B	UKBB	2017
rs395962	G	T	Basal metabolic rate	Neale B	UKBB	2017
rs395962	G	T	Comparative body size at age 10	Neale B	UKBB	2017
rs395962	G	T	Forced expiratory volume in 1-second, predicted	Neale B	UKBB	2017
rs395962	G	T	Forced vital capacity	Neale B	UKBB	2017
rs395962	G	T	Forced vital capacity, best measure	Neale B	UKBB	2017
rs395962	G	T	Hand grip strength right	Neale B	UKBB	2017
rs395962	G	T	Height	Neale B	UKBB	2017
rs395962	G	T	Impedance of arm left	Neale B	UKBB	2017
rs395962	G	T	Impedance of arm right	Neale B	UKBB	2017
rs395962	G	T	Impedance of leg left	Neale B	UKBB	2017
rs395962	G	T	Impedance of leg right	Neale B	UKBB	2017
rs395962	G	T	Impedance of whole body	Neale B	UKBB	2017
rs395962	G	T	Leg fat-free mass left	Neale B	UKBB	2017
rs395962	G	T	Leg predicted mass left	Neale B	UKBB	2017
rs395962	G	T	Relative age of first facial hair	Neale B	UKBB	2017
rs395962	G	T	Relative age voice broke	Neale B	UKBB	2017
rs395962	G	T	Sitting height	Neale B	UKBB	2017
rs395962	G	T	Trunk fat mass	Neale B	UKBB	2017
rs395962	G	T	Trunk fat-free mass	Neale B	UKBB	2017
rs395962	G	T	Trunk predicted mass	Neale B	UKBB	2017
rs395962	G	T	Weight	Neale B	UKBB	2017
rs395962	G	T	Whole body fat-free mass	Neale B	UKBB	2017
rs395962	G	T	Whole body water mass	Neale B	UKBB	2017
rs395962	G	T	Age at menarche	ReproGen	25231870	2014
rs4340786	A	T	Age at menarche	Neale B	UKBB	2017
rs437836	T	C	Comparative height size at age 10	Neale B	UKBB	2017
rs437836	T	C	Sitting height	Neale B	UKBB	2017
rs4561063	G	T	Age at menarche	Neale B	UKBB	2017

rs4804025	A	G	Hematocrit	Astle W	27863252	2016
rs4804025	A	G	Hemoglobin concentration	Astle W	27863252	2016
rs4804025	A	G	Red blood cell count	Astle W	27863252	2016
rs4804025	A	G	Arm fat mass left	Neale B	UKBB	2017
rs4804025	A	G	Arm fat mass right	Neale B	UKBB	2017
rs4804025	A	G	Arm fat percentage left	Neale B	UKBB	2017
rs4804025	A	G	Arm fat percentage right	Neale B	UKBB	2017
rs4804025	A	G	Arm fat-free mass left	Neale B	UKBB	2017
rs4804025	A	G	Arm fat-free mass right	Neale B	UKBB	2017
rs4804025	A	G	Arm predicted mass left	Neale B	UKBB	2017
rs4804025	A	G	Arm predicted mass right	Neale B	UKBB	2017
rs4804025	A	G	Basal metabolic rate	Neale B	UKBB	2017
rs4804025	A	G	Body fat percentage	Neale B	UKBB	2017
rs4804025	A	G	Body mass index	Neale B	UKBB	2017
rs4804025	A	G	Comparative body size at age 10	Neale B	UKBB	2017
rs4804025	A	G	Hip circumference	Neale B	UKBB	2017
rs4804025	A	G	Impedance of arm left	Neale B	UKBB	2017
rs4804025	A	G	Impedance of arm right	Neale B	UKBB	2017
rs4804025	A	G	Impedance of leg left	Neale B	UKBB	2017
rs4804025	A	G	Impedance of leg right	Neale B	UKBB	2017
rs4804025	A	G	Impedance of whole body	Neale B	UKBB	2017
rs4804025	A	G	Leg fat mass left	Neale B	UKBB	2017
rs4804025	A	G	Leg fat mass right	Neale B	UKBB	2017
rs4804025	A	G	Leg fat percentage left	Neale B	UKBB	2017
rs4804025	A	G	Leg fat percentage right	Neale B	UKBB	2017
rs4804025	A	G	Leg fat-free mass left	Neale B	UKBB	2017
rs4804025	A	G	Leg fat-free mass right	Neale B	UKBB	2017
rs4804025	A	G	Leg predicted mass left	Neale B	UKBB	2017
rs4804025	A	G	Leg predicted mass right	Neale B	UKBB	2017
rs4804025	A	G	Trunk fat mass	Neale B	UKBB	2017
rs4804025	A	G	Trunk fat percentage	Neale B	UKBB	2017
rs4804025	A	G	Trunk fat-free mass	Neale B	UKBB	2017
rs4804025	A	G	Trunk predicted mass	Neale B	UKBB	2017
rs4804025	A	G	Waist circumference	Neale B	UKBB	2017
rs4804025	A	G	Weight	Neale B	UKBB	2017
rs4804025	A	G	Whole body fat mass	Neale B	UKBB	2017
rs4804025	A	G	Whole body fat-free mass	Neale B	UKBB	2017
rs4804025	A	G	Whole body water mass	Neale B	UKBB	2017
rs4836984	C	T	Age at menarche	Neale B	UKBB	2017
rs4886140	A	G	Sleeplessness or insomnia	Neale B	UKBB	2017
rs4897178	G	T	Hematocrit	Astle W	27863252	2016
rs4897178	G	T	Hemoglobin concentration	Astle W	27863252	2016
rs4897178	G	T	Type II diabetes adjusted for BMI	DIAGRAM	28566273	2017
rs4897178	G	T	Type II diabetes	DIAGRAM	28566273	2017
rs4945266	A	G	Age at menarche	Neale B	UKBB	2017
rs4945266	A	G	Impedance of arm left	Neale B	UKBB	2017
rs4945266	A	G	Impedance of arm right	Neale B	UKBB	2017
rs4945266	A	G	Impedance of whole body	Neale B	UKBB	2017

rs506589	C	T	Body mass index females	Akiyama M	28892062	2017
rs506589	C	T	Body mass index	Akiyama M	28892062	2017
rs506589	C	T	Childhood BMI	EGGC	26604143	2016
rs506589	C	T	Body mass index in physically active females	GIANT	28448500	2017
rs506589	C	T	Body mass index in physically active indivdiuals	GIANT	28448500	2017
rs506589	C	T	Body mass index in physically active indivdiuals	GIANT	28448500	2017
rs506589	C	T	Body mass index in females greater than 50 years of age	GIANT	26426971	2015
rs506589	C	T	Body mass index in females less than or equal to 50 years of age	GIANT	26426971	2015
rs506589	C	T	Body mass index in females	GIANT	23754948	2013
rs506589	C	T	Body mass index in females	GIANT	25673413	2015
rs506589	C	T	Body mass index in males	GIANT	25673413	2015
rs506589	C	T	Body mass index in female non-smokers	GIANT	28443625	2017
rs506589	C	T	Body mass index in non-smokers	GIANT	28443625	2017
rs506589	C	T	Body mass index in non-smokers	GIANT	28443625	2017
rs506589	C	T	Body mass index tails	GIANT	23563607	2013
rs506589	C	T	Body mass index ajusted for physical activity in females	GIANT	28448500	2017
rs506589	C	T	Body mass index adjusted for physical activity	GIANT	28448500	2017
rs506589	C	T	Body mass index adjusted for physical activity	GIANT	28448500	2017
rs506589	C	T	Body mass index adjusted for smoking in females	GIANT	28443625	2017
rs506589	C	T	Body mass index adjusted for smoking	GIANT	28443625	2017
rs506589	C	T	Body mass index adjusted for smoking	GIANT	28443625	2017
rs506589	C	T	Body mass index	Speliotes EK	20935630	2010
rs506589	C	T	Body mass index	GIANT	23754948	2013
rs506589	C	T	Body mass index	GIANT	25673413	2015
rs506589	C	T	Body mass index	GIANT	25673413	2015
rs506589	C	T	Hip circumference in females	GIANT	25673412	2015
rs506589	C	T	Hip circumference	GIANT	25673412	2015
rs506589	C	T	Hip circumference	GIANT	25673412	2015
rs506589	C	T	Obesity class 1	GIANT	23563607	2013
rs506589	C	T	Obesity class 2	GIANT	23563607	2013
rs506589	C	T	Overweight	GIANT	23563607	2013
rs506589	C	T	Waist circumference in females	GIANT	25673412	2015
rs506589	C	T	Waist circumference	GIANT	23754948	2013
rs506589	C	T	Waist circumference	GIANT	25673412	2015
rs506589	C	T	Waist circumference	GIANT	25673412	2015
rs506589	C	T	Weight in females	GIANT	23754948	2013
rs506589	C	T	Weight	GIANT	23754948	2013
rs506589	C	T	Body mass index	Speliotes EK	20935630	2010
rs506589	C	T	Body mass index	Monda KL	23583978	2013
rs506589	C	T	Age at menarche	Neale B	UKBB	2017
rs506589	C	T	Arm fat mass left	Neale B	UKBB	2017
rs506589	C	T	Arm fat mass right	Neale B	UKBB	2017
rs506589	C	T	Arm fat percentage left	Neale B	UKBB	2017
rs506589	C	T	Arm fat percentage right	Neale B	UKBB	2017
rs506589	C	T	Arm fat-free mass left	Neale B	UKBB	2017
rs506589	C	T	Arm fat-free mass right	Neale B	UKBB	2017

rs506589	C	T	Arm predicted mass left	Neale B	UKBB	2017
rs506589	C	T	Arm predicted mass right	Neale B	UKBB	2017
rs506589	C	T	Basal metabolic rate	Neale B	UKBB	2017
rs506589	C	T	Body fat percentage	Neale B	UKBB	2017
rs506589	C	T	Body mass index	Neale B	UKBB	2017
rs506589	C	T	Comparative body size at age 10	Neale B	UKBB	2017
rs506589	C	T	Comparative height size at age 10	Neale B	UKBB	2017
rs506589	C	T	Hip circumference	Neale B	UKBB	2017
rs506589	C	T	Impedance of arm left	Neale B	UKBB	2017
rs506589	C	T	Impedance of arm right	Neale B	UKBB	2017
rs506589	C	T	Impedance of leg left	Neale B	UKBB	2017
rs506589	C	T	Impedance of leg right	Neale B	UKBB	2017
rs506589	C	T	Impedance of whole body	Neale B	UKBB	2017
rs506589	C	T	Leg fat mass left	Neale B	UKBB	2017
rs506589	C	T	Leg fat mass right	Neale B	UKBB	2017
rs506589	C	T	Leg fat percentage left	Neale B	UKBB	2017
rs506589	C	T	Leg fat percentage right	Neale B	UKBB	2017
rs506589	C	T	Leg fat-free mass left	Neale B	UKBB	2017
rs506589	C	T	Leg fat-free mass right	Neale B	UKBB	2017
rs506589	C	T	Leg predicted mass left	Neale B	UKBB	2017
rs506589	C	T	Leg predicted mass right	Neale B	UKBB	2017
rs506589	C	T	Trunk fat mass	Neale B	UKBB	2017
rs506589	C	T	Trunk fat percentage	Neale B	UKBB	2017
rs506589	C	T	Trunk fat-free mass	Neale B	UKBB	2017
rs506589	C	T	Trunk predicted mass	Neale B	UKBB	2017
rs506589	C	T	Waist circumference	Neale B	UKBB	2017
rs506589	C	T	Weight	Neale B	UKBB	2017
rs506589	C	T	Whole body fat mass	Neale B	UKBB	2017
rs506589	C	T	Whole body fat-free mass	Neale B	UKBB	2017
rs506589	C	T	Whole body water mass	Neale B	UKBB	2017
rs506589	C	T	Age at menarche	ReproGen	25231870	2014
rs552491	G	A	Age at menarche	Neale B	UKBB	2017
rs55680968	A	G	Diastolic blood pressure	Neale B	UKBB	2017
rs55680968	A	G	Heel bone mineral density	Neale B	UKBB	2017
rs55680968	A	G	Self-reported hypertension	Neale B	UKBB	2017
rs55680968	A	G	Vascular or heart problems diagnosed by doctor: high blood pressure	Neale B	UKBB	2017
rs55680968	A	G	Vascular or heart problems diagnosed by doctor: none of the above	Neale B	UKBB	2017
rs56409371	A	G	Age at menarche	Neale B	UKBB	2017
rs582780	A	G	Height in males	GIANT	23754948	2013
rs582780	A	G	Height tails	GIANT	23563607	2013
rs582780	A	G	Height	GIANT	20881960	2010
rs582780	A	G	Height	GIANT	23754948	2013
rs582780	A	G	Height	GIANT	25282103	2014
rs582780	A	G	Weight	GIANT	23754948	2013
rs582780	A	G	Height	GIANT	20881960	2010
rs582780	A	G	Arm fat-free mass left	Neale B	UKBB	2017
rs582780	A	G	Arm fat-free mass right	Neale B	UKBB	2017

rs582780	A	G	Arm predicted mass left	Neale B	UKBB	2017
rs582780	A	G	Arm predicted mass right	Neale B	UKBB	2017
rs582780	A	G	Basal metabolic rate	Neale B	UKBB	2017
rs582780	A	G	Comparative height size at age 10	Neale B	UKBB	2017
rs582780	A	G	Forced expiratory volume in 1-second	Neale B	UKBB	2017
rs582780	A	G	Forced vital capacity	Neale B	UKBB	2017
rs582780	A	G	Forced vital capacity, best measure	Neale B	UKBB	2017
rs582780	A	G	Height	Neale B	UKBB	2017
rs582780	A	G	Leg fat-free mass left	Neale B	UKBB	2017
rs582780	A	G	Leg fat-free mass right	Neale B	UKBB	2017
rs582780	A	G	Leg predicted mass left	Neale B	UKBB	2017
rs582780	A	G	Leg predicted mass right	Neale B	UKBB	2017
rs582780	A	G	Pulse rate	Neale B	UKBB	2017
rs582780	A	G	Sitting height	Neale B	UKBB	2017
rs582780	A	G	Trunk fat-free mass	Neale B	UKBB	2017
rs582780	A	G	Trunk predicted mass	Neale B	UKBB	2017
rs582780	A	G	Weight	Neale B	UKBB	2017
rs582780	A	G	Whole body fat-free mass	Neale B	UKBB	2017
rs582780	A	G	Whole body water mass	Neale B	UKBB	2017
rs6185	G	C	Length of menstrual cycle	Neale B	UKBB	2017
rs62104180	A	G	Age at menarche	Neale B	UKBB	2017
rs62104180	A	G	Arm fat mass left	Neale B	UKBB	2017
rs62104180	A	G	Arm fat mass right	Neale B	UKBB	2017
rs62104180	A	G	Arm fat percentage left	Neale B	UKBB	2017
rs62104180	A	G	Arm fat percentage right	Neale B	UKBB	2017
rs62104180	A	G	Arm fat-free mass left	Neale B	UKBB	2017
rs62104180	A	G	Arm fat-free mass right	Neale B	UKBB	2017
rs62104180	A	G	Arm predicted mass left	Neale B	UKBB	2017
rs62104180	A	G	Arm predicted mass right	Neale B	UKBB	2017
rs62104180	A	G	Basal metabolic rate	Neale B	UKBB	2017
rs62104180	A	G	Body fat percentage	Neale B	UKBB	2017
rs62104180	A	G	Body mass index	Neale B	UKBB	2017
rs62104180	A	G	Comparative body size at age 10	Neale B	UKBB	2017
rs62104180	A	G	Comparative height size at age 10	Neale B	UKBB	2017
rs62104180	A	G	Hip circumference	Neale B	UKBB	2017
rs62104180	A	G	Impedance of arm left	Neale B	UKBB	2017
rs62104180	A	G	Impedance of arm right	Neale B	UKBB	2017
rs62104180	A	G	Impedance of leg left	Neale B	UKBB	2017
rs62104180	A	G	Impedance of leg right	Neale B	UKBB	2017
rs62104180	A	G	Impedance of whole body	Neale B	UKBB	2017
rs62104180	A	G	Leg fat mass left	Neale B	UKBB	2017
rs62104180	A	G	Leg fat mass right	Neale B	UKBB	2017
rs62104180	A	G	Leg fat percentage left	Neale B	UKBB	2017
rs62104180	A	G	Leg fat percentage right	Neale B	UKBB	2017
rs62104180	A	G	Leg fat-free mass left	Neale B	UKBB	2017
rs62104180	A	G	Leg fat-free mass right	Neale B	UKBB	2017
rs62104180	A	G	Leg predicted mass left	Neale B	UKBB	2017
rs62104180	A	G	Leg predicted mass right	Neale B	UKBB	2017

rs62104180	A	G	Trunk fat mass	Neale B	UKBB	2017
rs62104180	A	G	Trunk fat percentage	Neale B	UKBB	2017
rs62104180	A	G	Trunk fat-free mass	Neale B	UKBB	2017
rs62104180	A	G	Trunk predicted mass	Neale B	UKBB	2017
rs62104180	A	G	Waist circumference	Neale B	UKBB	2017
rs62104180	A	G	Weight	Neale B	UKBB	2017
rs62104180	A	G	Whole body fat mass	Neale B	UKBB	2017
rs62104180	A	G	Whole body fat-free mass	Neale B	UKBB	2017
rs62104180	A	G	Whole body water mass	Neale B	UKBB	2017
rs62379978	G	T	Age at menarche	Neale B	UKBB	2017
rs62379978	G	T	Height	Neale B	UKBB	2017
rs62379978	G	T	Relative age of first facial hair	Neale B	UKBB	2017
rs62379978	G	T	Relative age voice broke	Neale B	UKBB	2017
rs643428	C	T	Comparative body size at age 10	Neale B	UKBB	2017
rs654354	T	A	Eosinophil count	Astle W	27863252	2016
rs654354	T	A	Eosinophil percentage of granulocytes	Astle W	27863252	2016
rs654354	T	A	Eosinophil percentage of white cells	Astle W	27863252	2016
rs654354	T	A	Neutrophil percentage of granulocytes	Astle W	27863252	2016
rs654354	T	A	Sum eosinophil basophil counts	Astle W	27863252	2016
rs654354	T	A	Allergic disease	Ferreira M	29083406	2017
rs654354	T	A	Asthma	Neale B	UKBB	2017
rs654354	T	A	Doctor diagnosed hayfever or allergic rhinitis	Neale B	UKBB	2017
rs654354	T	A	Hayfever, allergic rhinitis or eczema	Neale B	UKBB	2017
rs654354	T	A	No blood clot, bronchitis, emphysema, asthma, rhinitis, eczema or allergy diagnosed by doctor	Neale B	UKBB	2017
rs654354	T	A	Self-reported asthma	Neale B	UKBB	2017
rs654354	T	A	Self-reported hayfever or allergic rhinitis	Neale B	UKBB	2017
rs6590889	C	T	Age at menarche	Neale B	UKBB	2017
rs6678140	C	T	Eosinophil percentage of white cells	Astle W	27863252	2016
rs6678140	C	T	Allergic disease	Ferreira M	29083406	2017
rs6678140	C	T	Femoral neck bone mineral density	GEFOS	22504420	2012
rs6678140	C	T	Body fat percentage	Neale B	UKBB	2017
rs6678140	C	T	Diastolic blood pressure	Neale B	UKBB	2017
rs6678140	C	T	Hayfever, allergic rhinitis or eczema	Neale B	UKBB	2017
rs6678140	C	T	Heel bone mineral density	Neale B	UKBB	2017
rs6678140	C	T	Heel bone mineral density left	Neale B	UKBB	2017
rs6678140	C	T	Heel bone mineral density right	Neale B	UKBB	2017
rs6678140	C	T	No blood clot, bronchitis, emphysema, asthma, rhinitis, eczema or allergy diagnosed by doctor	Neale B	UKBB	2017
rs6678140	C	T	Systolic blood pressure	Neale B	UKBB	2017
rs6678140	C	T	Trunk fat percentage	Neale B	UKBB	2017
rs6678140	C	T	Schizophrenia	PGC	25056061	2014
rs6735626	A	G	Body mass index	Neale B	UKBB	2017
rs6735626	A	G	Impedance of arm left	Neale B	UKBB	2017
rs6735626	A	G	Impedance of arm right	Neale B	UKBB	2017
rs6735626	A	G	Impedance of whole body	Neale B	UKBB	2017
rs6864818	C	T	Age at menarche	ReproGen	25231870	2014
rs6931884	C	T	Age at menarche	Neale B	UKBB	2017
rs6931884	C	T	Age at menarche	ReproGen	25231870	2014

rs6933660	A	C	Menarche age at onset	ReproGen	25231870	2014
rs6933660	A	C	Age at menarche	Neale B	UKBB	2017
rs6933660	A	C	Heel bone mineral density	Neale B	UKBB	2017
rs6933660	A	C	Heel bone mineral density left	Neale B	UKBB	2017
rs6933660	A	C	Heel bone mineral density right	Neale B	UKBB	2017
rs6933660	A	C	Age at menarche	ReproGen	25231870	2014
rs7077302	C	G	Heel bone mineral density right	Neale B	UKBB	2017
rs7114175	A	T	Age at menarche	Neale B	UKBB	2017
rs7114175	A	T	Height	Neale B	UKBB	2017
rs7114175	A	T	Relative age of first facial hair	Neale B	UKBB	2017
rs7114175	A	T	Relative age voice broke	Neale B	UKBB	2017
rs7132908	A	G	Childhood BMI	EGGC	26604143	2016
rs7132908	A	G	Childhood obesity	EGGC	22484627	2012
rs7132908	A	G	Body mass index in physically active indivdiuals	GIANT	28448500	2017
rs7132908	A	G	Body mass index in females greater than 50 years of age	GIANT	26426971	2015
rs7132908	A	G	Body mass index in females less than or equal to 50 years of age	GIANT	26426971	2015
rs7132908	A	G	Body mass index in females	GIANT	25673413	2015
rs7132908	A	G	Body mass index in males	GIANT	25673413	2015
rs7132908	A	G	Body mass index in non-smokers	GIANT	28443625	2017
rs7132908	A	G	Body mass index adjusted for physical activity	GIANT	28448500	2017
rs7132908	A	G	Body mass index adjusted for physical activity	GIANT	28448500	2017
rs7132908	A	G	Body mass index adjusted for smoking in females	GIANT	28443625	2017
rs7132908	A	G	Body mass index adjusted for smoking	GIANT	28443625	2017
rs7132908	A	G	Body mass index adjusted for smoking	GIANT	28443625	2017
rs7132908	A	G	Body mass index	Speliotes EK	20935630	2010
rs7132908	A	G	Body mass index	GIANT	23754948	2013
rs7132908	A	G	Body mass index	GIANT	25673413	2015
rs7132908	A	G	Body mass index	GIANT	29273807	2018
rs7132908	A	G	Body mass index	GIANT	25673413	2015
rs7132908	A	G	Body mass index	GIANT	29273807	2018
rs7132908	A	G	Obesity class 1	GIANT	23563607	2013
rs7132908	A	G	Waist circumference	GIANT	25673412	2015
rs7132908	A	G	Waist circumference	GIANT	25673412	2015
rs7132908	A	G	Weight	GIANT	23754948	2013
rs7132908	A	G	Body mass index	Speliotes EK	20935630	2010
rs7132908	A	G	Body mass index	Paternoster L	21935397	2011
rs7132908	A	G	Body mass index 25 kgm2	GIANT	23563607	2013
rs7132908	A	G	Body mass index 30 kgm2	GIANT	23563607	2013
rs7132908	A	G	Body mass index 35 kgm2	GIANT	23563607	2013
rs7132908	A	G	Obesity body mass index	Paternoster L	21935397	2011
rs7132908	A	G	Obesity with early age of onset age 2	EGGC	22484627	2012
rs7132908	A	G	Childhood body mass index	EGGC	26604143	2016
rs7132908	A	G	Arm fat mass left	Neale B	UKBB	2017
rs7132908	A	G	Arm fat mass right	Neale B	UKBB	2017
rs7132908	A	G	Arm fat percentage left	Neale B	UKBB	2017
rs7132908	A	G	Arm fat percentage right	Neale B	UKBB	2017

rs7132908	A	G	Arm fat-free mass left	Neale B	UKBB	2017
rs7132908	A	G	Arm fat-free mass right	Neale B	UKBB	2017
rs7132908	A	G	Arm predicted mass left	Neale B	UKBB	2017
rs7132908	A	G	Arm predicted mass right	Neale B	UKBB	2017
rs7132908	A	G	Basal metabolic rate	Neale B	UKBB	2017
rs7132908	A	G	Body fat percentage	Neale B	UKBB	2017
rs7132908	A	G	Body mass index	Neale B	UKBB	2017
rs7132908	A	G	Comparative body size at age 10	Neale B	UKBB	2017
rs7132908	A	G	Comparative height size at age 10	Neale B	UKBB	2017
rs7132908	A	G	Hip circumference	Neale B	UKBB	2017
rs7132908	A	G	Impedance of arm left	Neale B	UKBB	2017
rs7132908	A	G	Impedance of arm right	Neale B	UKBB	2017
rs7132908	A	G	Impedance of leg left	Neale B	UKBB	2017
rs7132908	A	G	Impedance of leg right	Neale B	UKBB	2017
rs7132908	A	G	Impedance of whole body	Neale B	UKBB	2017
rs7132908	A	G	Leg fat mass left	Neale B	UKBB	2017
rs7132908	A	G	Leg fat mass right	Neale B	UKBB	2017
rs7132908	A	G	Leg fat percentage left	Neale B	UKBB	2017
rs7132908	A	G	Leg fat percentage right	Neale B	UKBB	2017
rs7132908	A	G	Leg fat-free mass left	Neale B	UKBB	2017
rs7132908	A	G	Leg fat-free mass right	Neale B	UKBB	2017
rs7132908	A	G	Leg predicted mass left	Neale B	UKBB	2017
rs7132908	A	G	Leg predicted mass right	Neale B	UKBB	2017
rs7132908	A	G	Trunk fat mass	Neale B	UKBB	2017
rs7132908	A	G	Trunk fat percentage	Neale B	UKBB	2017
rs7132908	A	G	Trunk fat-free mass	Neale B	UKBB	2017
rs7132908	A	G	Trunk predicted mass	Neale B	UKBB	2017
rs7132908	A	G	Waist circumference	Neale B	UKBB	2017
rs7132908	A	G	Weight	Neale B	UKBB	2017
rs7132908	A	G	Whole body fat mass	Neale B	UKBB	2017
rs7132908	A	G	Whole body fat-free mass	Neale B	UKBB	2017
rs7132908	A	G	Whole body water mass	Neale B	UKBB	2017
rs7178532	A	G	Age at menarche	Neale B	UKBB	2017
rs72787511	C	G	Hair or balding pattern: pattern 4	Neale B	UKBB	2017
rs72787511	C	G	Relative age of first facial hair	Neale B	UKBB	2017
rs7359336	A	G	Body mass index in non-smokers	GIANT	28443625	2017
rs7359336	A	G	Height	GIANT	25282103	2014
rs7359336	A	G	Age at menarche	Neale B	UKBB	2017
rs7359336	A	G	Arm fat mass left	Neale B	UKBB	2017
rs7359336	A	G	Arm fat mass right	Neale B	UKBB	2017
rs7359336	A	G	Arm fat percentage left	Neale B	UKBB	2017
rs7359336	A	G	Arm fat percentage right	Neale B	UKBB	2017
rs7359336	A	G	Body fat percentage	Neale B	UKBB	2017
rs7359336	A	G	Body mass index	Neale B	UKBB	2017
rs7359336	A	G	Diabetes diagnosed by doctor	Neale B	UKBB	2017
rs7359336	A	G	Forced expiratory volume in 1-second	Neale B	UKBB	2017
rs7359336	A	G	Forced expiratory volume in 1-second, best measure	Neale B	UKBB	2017

rs7359336	A	G	Forced vital capacity	Neale B	UKBB	2017
rs7359336	A	G	Forced vital capacity, best measure	Neale B	UKBB	2017
rs7359336	A	G	Hand grip strength left	Neale B	UKBB	2017
rs7359336	A	G	Height	Neale B	UKBB	2017
rs7359336	A	G	Hip circumference	Neale B	UKBB	2017
rs7359336	A	G	Impedance of leg left	Neale B	UKBB	2017
rs7359336	A	G	Impedance of leg right	Neale B	UKBB	2017
rs7359336	A	G	Leg fat mass left	Neale B	UKBB	2017
rs7359336	A	G	Leg fat mass right	Neale B	UKBB	2017
rs7359336	A	G	Leg fat percentage left	Neale B	UKBB	2017
rs7359336	A	G	Leg fat percentage right	Neale B	UKBB	2017
rs7359336	A	G	Self-reported diabetes	Neale B	UKBB	2017
rs7359336	A	G	Sitting height	Neale B	UKBB	2017
rs7359336	A	G	Trunk fat mass	Neale B	UKBB	2017
rs7359336	A	G	Trunk fat percentage	Neale B	UKBB	2017
rs7359336	A	G	Waist circumference	Neale B	UKBB	2017
rs7359336	A	G	Whole body fat mass	Neale B	UKBB	2017
rs7359336	A	G	Age at menarche	ReproGen	25231870	2014
rs7516763	A	C	Comparative body size at age 10	Neale B	UKBB	2017
rs7517629	A	G	Years of educational attainment	SSGAC	27225129	2016
rs7576624	C	T	Body mass index females	Akiyama M	28892062	2017
rs7576624	C	T	Body mass index males	Akiyama M	28892062	2017
rs7576624	C	T	Body mass index	Akiyama M	28892062	2017
rs7576624	C	T	Childhood BMI	EGGC	26604143	2016
rs7576624	C	T	Childhood obesity	EGGC	22484627	2012
rs7576624	C	T	Body mass index in physically active females	GIANT	28448500	2017
rs7576624	C	T	Body mass index in physically active males	GIANT	28448500	2017
rs7576624	C	T	Body mass index in physically active indivdiuals	GIANT	28448500	2017
rs7576624	C	T	Body mass index in physically active indivdiuals	GIANT	28448500	2017
rs7576624	C	T	Body mass index in females greater than 50 years of age	GIANT	26426971	2015
rs7576624	C	T	Body mass index in females less than or equal to 50 years of age	GIANT	26426971	2015
rs7576624	C	T	Body mass index in females	GIANT	23754948	2013
rs7576624	C	T	Body mass index in females	GIANT	25673413	2015
rs7576624	C	T	Body mass index in physically inactive females	GIANT	28448500	2017
rs7576624	C	T	Body mass index in physically inactive indivdiuals	GIANT	28448500	2017
rs7576624	C	T	Body mass index in physically inactive indivdiuals	GIANT	28448500	2017
rs7576624	C	T	Body mass index in males greater than 50 years of age	GIANT	26426971	2015
rs7576624	C	T	Body mass index in males less than or equal to 50 years of age	GIANT	26426971	2015
rs7576624	C	T	Body mass index in males	GIANT	23754948	2013
rs7576624	C	T	Body mass index in males	GIANT	25673413	2015
rs7576624	C	T	Body mass index in female non-smokers	GIANT	28443625	2017
rs7576624	C	T	Body mass index in male non-smokers	GIANT	28443625	2017
rs7576624	C	T	Body mass index in non-smokers	GIANT	28443625	2017
rs7576624	C	T	Body mass index in non-smokers	GIANT	28443625	2017
rs7576624	C	T	Body mass index in smokers	GIANT	28443625	2017
rs7576624	C	T	Body mass index in smokers	GIANT	28443625	2017

rs7576624	C	T	Body mass index tails	GIANT	23563607	2013
rs7576624	C	T	Body mass index ajusted for physical activity in females	GIANT	28448500	2017
rs7576624	C	T	Body mass index adjsuted for physical activity in males	GIANT	28448500	2017
rs7576624	C	T	Body mass index adjusted for physical activity	GIANT	28448500	2017
rs7576624	C	T	Body mass index adjusted for physical activity	GIANT	28448500	2017
rs7576624	C	T	Body mass index adjusted for smoking in females	GIANT	28443625	2017
rs7576624	C	T	Body mass index adjusted for smoking in males	GIANT	28443625	2017
rs7576624	C	T	Body mass index adjusted for smoking	GIANT	28443625	2017
rs7576624	C	T	Body mass index adjusted for smoking	GIANT	28443625	2017
rs7576624	C	T	Body mass index	Speliotes EK	20935630	2010
rs7576624	C	T	Body mass index	GIANT	23754948	2013
rs7576624	C	T	Body mass index	GIANT	25673413	2015
rs7576624	C	T	Body mass index	GIANT	25673413	2015
rs7576624	C	T	Hip circumference in females	GIANT	25673412	2015
rs7576624	C	T	Hip circumference in males	GIANT	25673412	2015
rs7576624	C	T	Hip circumference	GIANT	25673412	2015
rs7576624	C	T	Hip circumference	GIANT	25673412	2015
rs7576624	C	T	Obesity class 1	GIANT	23563607	2013
rs7576624	C	T	Obesity class 2	GIANT	23563607	2013
rs7576624	C	T	Overweight	GIANT	23563607	2013
rs7576624	C	T	Waist circumference in females	GIANT	25673412	2015
rs7576624	C	T	Waist circumference in males	GIANT	25673412	2015
rs7576624	C	T	Waist circumference	GIANT	23754948	2013
rs7576624	C	T	Waist circumference	GIANT	25673412	2015
rs7576624	C	T	Waist circumference	GIANT	25673412	2015
rs7576624	C	T	Weight in females	GIANT	23754948	2013
rs7576624	C	T	Weight in males	GIANT	23754948	2013
rs7576624	C	T	Weight	GIANT	23754948	2013
rs7576624	C	T	Body mass index	Speliotes EK	20935630	2010
rs7576624	C	T	Obesity with early age of onset age 2	EGGC	22484627	2012
rs7576624	C	T	Body fat percentage	Lu Y	26833246	2016
rs7576624	C	T	Age at menarche	Neale B	UKBB	2017
rs7576624	C	T	Arm fat mass left	Neale B	UKBB	2017
rs7576624	C	T	Arm fat mass right	Neale B	UKBB	2017
rs7576624	C	T	Arm fat percentage left	Neale B	UKBB	2017
rs7576624	C	T	Arm fat percentage right	Neale B	UKBB	2017
rs7576624	C	T	Arm fat-free mass left	Neale B	UKBB	2017
rs7576624	C	T	Arm fat-free mass right	Neale B	UKBB	2017
rs7576624	C	T	Arm predicted mass left	Neale B	UKBB	2017
rs7576624	C	T	Arm predicted mass right	Neale B	UKBB	2017
rs7576624	C	T	Basal metabolic rate	Neale B	UKBB	2017
rs7576624	C	T	Body fat percentage	Neale B	UKBB	2017
rs7576624	C	T	Body mass index	Neale B	UKBB	2017
rs7576624	C	T	Comparative body size at age 10	Neale B	UKBB	2017
rs7576624	C	T	Comparative height size at age 10	Neale B	UKBB	2017
rs7576624	C	T	Hip circumference	Neale B	UKBB	2017

rs7576624	C	T	Impedance of arm left	Neale B	UKBB	2017
rs7576624	C	T	Impedance of arm right	Neale B	UKBB	2017
rs7576624	C	T	Impedance of leg left	Neale B	UKBB	2017
rs7576624	C	T	Impedance of leg right	Neale B	UKBB	2017
rs7576624	C	T	Impedance of whole body	Neale B	UKBB	2017
rs7576624	C	T	Leg fat mass left	Neale B	UKBB	2017
rs7576624	C	T	Leg fat mass right	Neale B	UKBB	2017
rs7576624	C	T	Leg fat percentage left	Neale B	UKBB	2017
rs7576624	C	T	Leg fat percentage right	Neale B	UKBB	2017
rs7576624	C	T	Leg fat-free mass left	Neale B	UKBB	2017
rs7576624	C	T	Leg fat-free mass right	Neale B	UKBB	2017
rs7576624	C	T	Leg predicted mass left	Neale B	UKBB	2017
rs7576624	C	T	Leg predicted mass right	Neale B	UKBB	2017
rs7576624	C	T	Trunk fat mass	Neale B	UKBB	2017
rs7576624	C	T	Trunk fat percentage	Neale B	UKBB	2017
rs7576624	C	T	Trunk fat-free mass	Neale B	UKBB	2017
rs7576624	C	T	Trunk predicted mass	Neale B	UKBB	2017
rs7576624	C	T	Waist circumference	Neale B	UKBB	2017
rs7576624	C	T	Weight	Neale B	UKBB	2017
rs7576624	C	T	Whole body fat mass	Neale B	UKBB	2017
rs7576624	C	T	Whole body fat-free mass	Neale B	UKBB	2017
rs7576624	C	T	Whole body water mass	Neale B	UKBB	2017
rs7576624	C	T	Age at menarche	ReproGen	25231870	2014
rs758747	C	T	Body mass index	GIANT	25673413	2015
rs758747	C	T	Body mass index	GIANT	25673413	2015
rs758747	C	T	Body mass index	GIANT	25673413	2015
rs758747	C	T	Body mass index	GIANT	25673413	2015
rs758747	C	T	Forced vital capacity	Neale B	UKBB	2017
rs758747	C	T	Forced vital capacity, best measure	Neale B	UKBB	2017
rs7852169	C	G	Age at menarche	Neale B	UKBB	2017
rs7852169	C	G	Relative age of first facial hair	Neale B	UKBB	2017
rs7853970	C	T	Menarche age at onset	ReproGen	25231870	2014
rs7853970	C	T	Age at menarche	Neale B	UKBB	2017
rs7853970	C	T	Age at menarche	ReproGen	25231870	2014
rs7907759	A	G	Age at menarche	Neale B	UKBB	2017
rs8051833	A	G	Alcohol intake frequency	Neale B	UKBB	2017
rs8051833	A	G	Arm fat mass right	Neale B	UKBB	2017
rs8051833	A	G	Arm fat-free mass left	Neale B	UKBB	2017
rs8051833	A	G	Arm fat-free mass right	Neale B	UKBB	2017
rs8051833	A	G	Arm predicted mass left	Neale B	UKBB	2017
rs8051833	A	G	Arm predicted mass right	Neale B	UKBB	2017
rs8051833	A	G	Basal metabolic rate	Neale B	UKBB	2017
rs8051833	A	G	Impedance of leg right	Neale B	UKBB	2017
rs8051833	A	G	Leg fat-free mass left	Neale B	UKBB	2017
rs8051833	A	G	Leg fat-free mass right	Neale B	UKBB	2017
rs8051833	A	G	Leg predicted mass left	Neale B	UKBB	2017
rs8051833	A	G	Leg predicted mass right	Neale B	UKBB	2017
rs8051833	A	G	Trunk fat-free mass	Neale B	UKBB	2017

rs8051833	A	G	Trunk predicted mass	Neale B	UKBB	2017
rs8051833	A	G	Waist circumference	Neale B	UKBB	2017
rs8051833	A	G	Weight	Neale B	UKBB	2017
rs8051833	A	G	Whole body fat-free mass	Neale B	UKBB	2017
rs8051833	A	G	Whole body water mass	Neale B	UKBB	2017
rs813301	C	T	Impedance of arm left	Neale B	UKBB	2017
rs813301	C	T	Impedance of arm right	Neale B	UKBB	2017
rs813301	C	T	Impedance of leg left	Neale B	UKBB	2017
rs813301	C	T	Impedance of leg right	Neale B	UKBB	2017
rs813301	C	T	Impedance of whole body	Neale B	UKBB	2017
rs913588	A	G	Menarche age at onset	ReproGen	25231870	2014
rs913588	A	G	Age at menarche	Neale B	UKBB	2017
rs913588	A	G	Age at menarche	ReproGen	25231870	2014
rs9349203	A	G	Granulocyte percentage of myeloid white cells	Astle W	27863252	2016
rs9349203	A	G	Mean corpuscular hemoglobin	Astle W	27863252	2016
rs9349203	A	G	Mean corpuscular volume	Astle W	27863252	2016
rs9349203	A	G	Monocyte count	Astle W	27863252	2016
rs9349203	A	G	Monocyte percentage of white cells	Astle W	27863252	2016
rs9349203	A	G	Red blood cell count	Astle W	27863252	2016
rs9349203	A	G	Red cell distribution width	Astle W	27863252	2016
rs9349203	A	G	Menarche age at onset	Pickrell JK	27182965	2016
rs9349203	A	G	Age at menarche	Neale B	UKBB	2017
rs9349203	A	G	Arm fat-free mass left	Neale B	UKBB	2017
rs9349203	A	G	Arm fat-free mass right	Neale B	UKBB	2017
rs9349203	A	G	Arm predicted mass left	Neale B	UKBB	2017
rs9349203	A	G	Arm predicted mass right	Neale B	UKBB	2017
rs9349203	A	G	Basal metabolic rate	Neale B	UKBB	2017
rs9349203	A	G	Height	Neale B	UKBB	2017
rs9349203	A	G	Hip circumference	Neale B	UKBB	2017
rs9349203	A	G	Leg fat-free mass left	Neale B	UKBB	2017
rs9349203	A	G	Leg fat-free mass right	Neale B	UKBB	2017
rs9349203	A	G	Leg predicted mass left	Neale B	UKBB	2017
rs9349203	A	G	Leg predicted mass right	Neale B	UKBB	2017
rs9349203	A	G	Relative age of first facial hair	Neale B	UKBB	2017
rs9349203	A	G	Sitting height	Neale B	UKBB	2017
rs9349203	A	G	Trunk fat-free mass	Neale B	UKBB	2017
rs9349203	A	G	Trunk predicted mass	Neale B	UKBB	2017
rs9349203	A	G	Weight	Neale B	UKBB	2017
rs9349203	A	G	Whole body fat-free mass	Neale B	UKBB	2017
rs9349203	A	G	Whole body water mass	Neale B	UKBB	2017
rs9382676	C	T	Age at menarche	Neale B	UKBB	2017
rs9474996	A	T	Age at menarche	ReproGen	25231870	2014
rs9522262	C	G	Arm fat mass left	Neale B	UKBB	2017
rs9522262	C	G	Arm fat percentage left	Neale B	UKBB	2017
rs9522262	C	G	Body fat percentage	Neale B	UKBB	2017
rs9522262	C	G	Body mass index	Neale B	UKBB	2017
rs9522262	C	G	Leg fat mass left	Neale B	UKBB	2017
rs9522262	C	G	Leg fat mass right	Neale B	UKBB	2017

rs9522262	C	G	Leg fat percentage left	Neale B	UKBB	2017
rs9522262	C	G	Leg fat percentage right	Neale B	UKBB	2017
rs9522262	C	G	Pack years adult smoking as proportion of life span exposed to smoking	Neale B	UKBB	2017
rs9522262	C	G	Pack years of smoking preview only	Neale B	UKBB	2017
rs9522262	C	G	Trunk fat mass	Neale B	UKBB	2017
rs9522262	C	G	Trunk fat percentage	Neale B	UKBB	2017
rs9522262	C	G	Waist circumference	Neale B	UKBB	2017
rs9522262	C	G	Whole body fat mass	Neale B	UKBB	2017
rs9522262	C	G	Age at menarche	ReproGen	25231870	2014
rs953230	A	G	Height	Neale B	UKBB	2017
rs953230	A	G	Sitting height	Neale B	UKBB	2017
rs953230	A	G	Age at menarche	ReproGen	25231870	2014
rs9548873	C	T	Eosinophil count	Astle W	27863252	2016
rs9548873	C	T	Eosinophil percentage of granulocytes	Astle W	27863252	2016
rs9548873	C	T	Eosinophil percentage of white cells	Astle W	27863252	2016
rs9548873	C	T	Neutrophil percentage of granulocytes	Astle W	27863252	2016
rs9548873	C	T	Sum eosinophil basophil counts	Astle W	27863252	2016
rs9548873	C	T	Rheumatoid arthritis	Okada Y	24390342	2014
rs9548873	C	T	Rheumatoid arthritis	Okada Y	24390342	2014
rs9548873	C	T	Age at menarche	ReproGen	25231870	2014
rs9548873	C	T	Eosinophil count	Astle W	27863252	2016
rs9548873	C	T	Eosinophil percentage of granulocytes	Astle W	27863252	2016
rs9548873	C	T	Eosinophil percentage of white cells	Astle W	27863252	2016
rs9548873	C	T	Neutrophil percentage of granulocytes	Astle W	27863252	2016
rs9548873	C	T	Sum eosinophil basophil counts	Astle W	27863252	2016
rs9548873	C	T	Rheumatoid arthritis	Okada Y	24390342	2014
rs9548873	C	T	Rheumatoid arthritis	Okada Y	24390342	2014
rs9548873	C	T	Age at menarche	ReproGen	25231870	2014
rs9635759	A	G	Age at menarche	Elks CE	21102462	2010
rs9635759	A	G	Menarche age at onset	Elks CE	21102462	2010
rs9635759	A	G	Menarche age at onset	ReproGen	25231870	2014
rs9635759	A	G	Menarche age at onset	Pickrell JK	27182965	2016
rs9635759	A	G	Age at menarche	Neale B	UKBB	2017
rs9635759	A	G	Age at menarche	ReproGen	25231870	2014
rs9635759	A	G	Menarche	Elks CE	21102462	2010
rs9647570	G	T	Menarche age at onset	ReproGen	25231870	2014
rs9647570	G	T	Age at menarche	ReproGen	25231870	2014
rs9758500	A	G	Age at menarche	Neale B	UKBB	2017
rs9758500	A	G	Comparative height size at age 10	Neale B	UKBB	2017
rs9972653	G	T	Body mass index females	Akiyama M	28892062	2017
rs9972653	G	T	Body mass index males	Akiyama M	28892062	2017
rs9972653	G	T	Body mass index	Akiyama M	28892062	2017
rs9972653	G	T	Type II diabetes	DIAGRAM	26551672	2015
rs9972653	G	T	Type II diabetes	DIAGRAM	28566273	2017
rs9972653	G	T	Age at menarche	Neale B	UKBB	2017
rs9972653	G	T	Alcohol intake frequency	Neale B	UKBB	2017
rs9972653	G	T	Arm fat mass left	Neale B	UKBB	2017

rs9972653	G	T	Arm fat mass right	Neale B	UKBB	2017
rs9972653	G	T	Arm fat percentage left	Neale B	UKBB	2017
rs9972653	G	T	Arm fat percentage right	Neale B	UKBB	2017
rs9972653	G	T	Arm fat-free mass left	Neale B	UKBB	2017
rs9972653	G	T	Arm fat-free mass right	Neale B	UKBB	2017
rs9972653	G	T	Arm predicted mass left	Neale B	UKBB	2017
rs9972653	G	T	Arm predicted mass right	Neale B	UKBB	2017
rs9972653	G	T	Average weekly beer plus cider intake	Neale B	UKBB	2017
rs9972653	G	T	Average weekly red wine intake	Neale B	UKBB	2017
rs9972653	G	T	Basal metabolic rate	Neale B	UKBB	2017
rs9972653	G	T	Body fat percentage	Neale B	UKBB	2017
rs9972653	G	T	Body mass index	Neale B	UKBB	2017
rs9972653	G	T	Comparative body size at age 10	Neale B	UKBB	2017
rs9972653	G	T	Diabetes diagnosed by doctor	Neale B	UKBB	2017
rs9972653	G	T	Getting up in morning	Neale B	UKBB	2017
rs9972653	G	T	Heel bone mineral density	Neale B	UKBB	2017
rs9972653	G	T	Hip circumference	Neale B	UKBB	2017
rs9972653	G	T	Illnesses of father: diabetes	Neale B	UKBB	2017
rs9972653	G	T	Impedance of arm left	Neale B	UKBB	2017
rs9972653	G	T	Impedance of arm right	Neale B	UKBB	2017
rs9972653	G	T	Impedance of leg left	Neale B	UKBB	2017
rs9972653	G	T	Impedance of leg right	Neale B	UKBB	2017
rs9972653	G	T	Impedance of whole body	Neale B	UKBB	2017
rs9972653	G	T	Leg fat mass left	Neale B	UKBB	2017
rs9972653	G	T	Leg fat mass right	Neale B	UKBB	2017
rs9972653	G	T	Leg fat percentage left	Neale B	UKBB	2017
rs9972653	G	T	Leg fat percentage right	Neale B	UKBB	2017
rs9972653	G	T	Leg fat-free mass left	Neale B	UKBB	2017
rs9972653	G	T	Leg fat-free mass right	Neale B	UKBB	2017
rs9972653	G	T	Leg predicted mass left	Neale B	UKBB	2017
rs9972653	G	T	Leg predicted mass right	Neale B	UKBB	2017
rs9972653	G	T	Medication for cholesterol, blood pressure or diabetes: none of the above	Neale B	UKBB	2017
rs9972653	G	T	Morning or evening person	Neale B	UKBB	2017
rs9972653	G	T	Self-reported breast cancer	Neale B	UKBB	2017
rs9972653	G	T	Self-reported diabetes	Neale B	UKBB	2017
rs9972653	G	T	Self-reported hypertension	Neale B	UKBB	2017
rs9972653	G	T	Sleep duration	Neale B	UKBB	2017
rs9972653	G	T	Snoring	Neale B	UKBB	2017
rs9972653	G	T	Sodium in urine	Neale B	UKBB	2017
rs9972653	G	T	Treatment with blood pressure medication	Neale B	UKBB	2017
rs9972653	G	T	Treatment with metformin	Neale B	UKBB	2017
rs9972653	G	T	Trunk fat mass	Neale B	UKBB	2017
rs9972653	G	T	Trunk fat percentage	Neale B	UKBB	2017
rs9972653	G	T	Trunk fat-free mass	Neale B	UKBB	2017
rs9972653	G	T	Trunk predicted mass	Neale B	UKBB	2017
rs9972653	G	T	Usual walking pace	Neale B	UKBB	2017
rs9972653	G	T	Vascular or heart problems diagnosed by doctor: high blood pressure	Neale B	UKBB	2017

rs9972653	G	T	Vascular or heart problems diagnosed by doctor: none of the above	Neale B	UKBB	2017
rs9972653	G	T	Waist circumference	Neale B	UKBB	2017
rs9972653	G	T	Weight	Neale B	UKBB	2017
rs9972653	G	T	Whole body fat mass	Neale B	UKBB	2017
rs9972653	G	T	Whole body fat-free mass	Neale B	UKBB	2017
rs9972653	G	T	Whole body water mass	Neale B	UKBB	2017
rs999885	G	A	Pulse rate	Neale B	UKBB	2017

Table S11 – Phenotype associations at genome-wide significance level ($p<5x10^{-8}$) of instrumental variants for age at menopause on PhenoScanner. SNP= single-nucleotide polymorphism, PMID = PubMed ID.

SNP	Alllele 1	Allele 2	Trait	Study	PMID/Source	Year
rs10255049	A	G	Height	Neale B	UKBB	2017
rs1044595	T	C	Ever used hormone-replacement therapy	Neale B	UKBB	2017
rs10477172	C	T	Self-reported testicular cancer	Neale B	UKBB	2017
rs10743724	C	T	Forced expiratory volume in 1-second	Neale B	UKBB	2017
rs10743724	C	T	Forced expiratory volume in 1-second, best measure	Neale B	UKBB	2017
rs10743724	C	T	Forced vital capacity	Neale B	UKBB	2017
rs10743724	C	T	Forced vital capacity, best measure	Neale B	UKBB	2017
rs10769315	C	T	Diastolic blood pressure	Neale B	UKBB	2017
rs10769315	C	T	Height	GIANT	25282103	2014
rs10769315	C	T	Height	Neale B	UKBB	2017
rs10769315	C	T	Medication for cholesterol, blood pressure or diabetes: blood pressure medication	Neale B	UKBB	2017
rs10769315	C	T	Self-reported hypertension	Neale B	UKBB	2017
rs10769315	C	T	Systolic blood pressure	Neale B	UKBB	2017
rs10769315	C	T	Vascular or heart problems diagnosed by doctor: high blood pressure	Neale B	UKBB	2017
rs10769315	C	T	Vascular or heart problems diagnosed by doctor: none of the above	Neale B	UKBB	2017
rs10823203	C	G	Arm fat-free mass left	Neale B	UKBB	2017
rs10823203	C	G	Arm fat-free mass right	Neale B	UKBB	2017
rs10823203	C	G	Arm predicted mass right	Neale B	UKBB	2017
rs10823203	C	G	Basal metabolic rate	Neale B	UKBB	2017
rs10823203	C	G	Comparative height size at age 10	Neale B	UKBB	2017
rs10823203	C	G	Height	Neale B	UKBB	2017
rs10823203	C	G	Leg fat-free mass left	Neale B	UKBB	2017
rs10823203	C	G	Leg fat-free mass right	Neale B	UKBB	2017
rs10823203	C	G	Leg predicted mass left	Neale B	UKBB	2017
rs10823203	C	G	Leg predicted mass right	Neale B	UKBB	2017
rs10823203	C	G	Sitting height	Neale B	UKBB	2017
rs10823203	C	G	Trunk fat-free mass	Neale B	UKBB	2017
rs10823203	C	G	Trunk predicted mass	Neale B	UKBB	2017
rs10823203	C	G	Whole body fat-free mass	Neale B	UKBB	2017
rs10823203	C	G	Whole body water mass	Neale B	UKBB	2017
rs10899493	C	T	Impedance of arm left	Neale B	UKBB	2017
rs10899493	C	T	Impedance of arm right	Neale B	UKBB	2017
rs10899493	C	T	Impedance of whole body	Neale B	UKBB	2017
rs11031006	A	G	Bilateral oophorectomy	Neale B	UKBB	2017
rs11031006	A	G	Excessive, frequent and irregular menstruation	Neale B	UKBB	2017

rs11031006	A	G	Length of menstrual cycle	Neale B	UKBB	2017
rs11031006	A	G	Luteinizing hormone levels in polycystic ovary syndrome	Hayes MG	26284813	2015
rs11031006	A	G	Polycystic ovary syndrome	Hayes MG	26284813	2015
rs11031006	A	G	Polycystic ovary syndrome	Day FR	26416764	2015
rs11031006	A	G	Spontaneous dizygotic twinning	Mbarek H	27132594	2016
rs11031006	A	G	Years since last cervical smear test	Neale B	UKBB	2017
rs11571815	A	G	Home area population density: Scotland large urban area	Neale B	UKBB	2017
rs11571815	A	G	Illnesses of father: lung cancer	Neale B	UKBB	2017
rs11668344	A	G	Ever used hormone-replacement therapy	Neale B	UKBB	2017
rs11668344	A	G	Had menopause	Neale B	UKBB	2017
rs11668344	A	G	Primary ovarian insufficient menopause 40	Perry JR	23307926	2013
rs11699793	C	T	Arm fat-free mass left	Neale B	UKBB	2017
rs11699793	C	T	Arm fat-free mass right	Neale B	UKBB	2017
rs11699793	C	T	Arm predicted mass left	Neale B	UKBB	2017
rs11699793	C	T	Arm predicted mass right	Neale B	UKBB	2017
rs11699793	C	T	Basal metabolic rate	Neale B	UKBB	2017
rs11699793	C	T	Comparative height size at age 10	Neale B	UKBB	2017
rs11699793	C	T	Height	Neale B	UKBB	2017
rs11699793	C	T	Impedance of leg left	Neale B	UKBB	2017
rs11699793	C	T	Impedance of leg right	Neale B	UKBB	2017
rs11699793	C	T	Impedance of whole body	Neale B	UKBB	2017
rs11699793	C	T	Leg fat-free mass left	Neale B	UKBB	2017
rs11699793	C	T	Leg fat-free mass right	Neale B	UKBB	2017
rs11699793	C	T	Leg predicted mass left	Neale B	UKBB	2017
rs11699793	C	T	Leg predicted mass right	Neale B	UKBB	2017
rs11699793	C	T	Sitting height	Neale B	UKBB	2017
rs11699793	C	T	Trunk fat-free mass	Neale B	UKBB	2017
rs11699793	C	T	Trunk predicted mass	Neale B	UKBB	2017
rs11699793	C	T	Weight	Neale B	UKBB	2017
rs11699793	C	T	Whole body fat-free mass	Neale B	UKBB	2017
rs11699793	C	T	Whole body water mass	Neale B	UKBB	2017
rs11767307	C	G	Arm fat-free mass left	Neale B	UKBB	2017
rs11767307	C	G	Arm fat-free mass right	Neale B	UKBB	2017
rs11767307	C	G	Arm predicted mass left	Neale B	UKBB	2017
rs11767307	C	G	Arm predicted mass right	Neale B	UKBB	2017
rs11767307	C	G	Basal metabolic rate	Neale B	UKBB	2017
rs11767307	C	G	Birth weight	Neale B	UKBB	2017
rs11767307	C	G	Comparative height size at age 10	Neale B	UKBB	2017
rs11767307	C	G	Forced expiratory volume in 1-second	Neale B	UKBB	2017
rs11767307	C	G	Forced expiratory volume in 1-second, best measure	Neale B	UKBB	2017
rs11767307	C	G	Forced vital capacity	Neale B	UKBB	2017

rs11767307	C	G	Forced vital capacity, best measure	Neale B	UKBB	2017
rs11767307	C	G	Height	GIANT	25282103	2014
rs11767307	C	G	Height	Neale B	UKBB	2017
rs11767307	C	G	Leg fat-free mass left	Neale B	UKBB	2017
rs11767307	C	G	Leg fat-free mass right	Neale B	UKBB	2017
rs11767307	C	G	Leg predicted mass left	Neale B	UKBB	2017
rs11767307	C	G	Leg predicted mass right	Neale B	UKBB	2017
rs11767307	C	G	Sitting height	Neale B	UKBB	2017
rs11767307	C	G	Trunk fat-free mass	Neale B	UKBB	2017
rs11767307	C	G	Trunk predicted mass	Neale B	UKBB	2017
rs11767307	C	G	Whole body fat-free mass	Neale B	UKBB	2017
rs11767307	C	G	Whole body water mass	Neale B	UKBB	2017
rs12053063	A	G	Impedance of arm left	Neale B	UKBB	2017
rs12605881	A	T	Hair or balding pattern: pattern 4	Neale B	UKBB	2017
rs1264191	C	T	Height	Neale B	UKBB	2017
rs1264191	C	T	High light scatter percentage of red cells	Astle W	27863252	2016
rs1264191	C	T	High light scatter reticulocyte count	Astle W	27863252	2016
rs1264191	C	T	Pulse rate	Neale B	UKBB	2017
rs1264191	C	T	Reticulocyte count	Astle W	27863252	2016
rs1264191	C	T	Reticulocyte fraction of red cells	Astle W	27863252	2016
rs1264191	C	T	Sitting height	Neale B	UKBB	2017
rs12825762	A	G	Basal metabolic rate	Neale B	UKBB	2017
rs12825762	A	G	Height	Neale B	UKBB	2017
rs12825762	A	G	Leg fat-free mass left	Neale B	UKBB	2017
rs12825762	A	G	Leg fat-free mass right	Neale B	UKBB	2017
rs12825762	A	G	Leg predicted mass left	Neale B	UKBB	2017
rs12825762	A	G	Leg predicted mass right	Neale B	UKBB	2017
rs12825762	A	G	Mean platelet volume	Astle W	27863252	2016
rs12825762	A	G	Weight	Neale B	UKBB	2017
rs12879626	G	T	Impedance of leg right	Neale B	UKBB	2017
rs12879626	G	T	Leg fat-free mass left	Neale B	UKBB	2017
rs12879626	G	T	Leg fat-free mass right	Neale B	UKBB	2017
rs12879626	G	T	Leg predicted mass left	Neale B	UKBB	2017
rs12879626	G	T	Leg predicted mass right	Neale B	UKBB	2017
rs12898357	A	G	Height	GIANT	25282103	2014
rs138430	C	T	Pulse rate	Neale B	UKBB	2017
rs1467044	A	G	Height	Neale B	UKBB	2017
rs1467044	A	G	Sitting height	Neale B	UKBB	2017
rs1565920	G	A	Allergic disease	Ferreira M	29083406	2017
rs1565920	G	A	Asthma	Neale B	UKBB	2017
rs1565920	G	A	Basophil count	Astle W	27863252	2016
rs1565920	G	A	Doctor diagnosed asthma	Neale B	UKBB	2017

rs1565920	G	A	Forced expiratory volume in 1-second, predicted percentage	Neale B	UKBB	2017
rs1565920	G	A	Granulocyte count	Astle W	27863252	2016
rs1565920	G	A	Granulocyte percentage of myeloid white cells	Astle W	27863252	2016
rs1565920	G	A	Inflammatory bowel disease	IBDGC	26192919	2015
rs1565920	G	A	Monocyte percentage of white cells	Astle W	27863252	2016
rs1565920	G	A	Myeloid white cell count	Astle W	27863252	2016
rs1565920	G	A	Neutrophil count	Astle W	27863252	2016
rs1565920	G	A	No blood clot, bronchitis, emphysema, asthma, rhinitis, eczema or allergy diagnosed by doctor	Neale B	UKBB	2017
rs1565920	G	A	Rheumatoid arthritis	Okada Y	24390342	2014
rs1565920	G	A	Self-reported asthma	Neale B	UKBB	2017
rs1565920	G	A	Sum basophil neutrophil counts	Astle W	27863252	2016
rs1565920	G	A	Sum neutrophil eosinophil counts	Astle W	27863252	2016
rs1565920	G	A	White blood cell count	Astle W	27863252	2016
rs16991615	A	G	Breast cancer	Michailidou K	29059683	2017
rs16991615	A	G	Ever used hormone-replacement therapy	Neale B	UKBB	2017
rs16991615	A	G	Had menopause	Neale B	UKBB	2017
rs16991615	A	G	Menarche	He C	19448621	2009
rs17680522	A	G	Forced expiratory volume in 1-second, predicted	Neale B	UKBB	2017
rs17680522	A	G	Height	Neale B	UKBB	2017
rs17680522	A	G	Sitting height	Neale B	UKBB	2017
rs1991401	A	G	Heel bone mineral density	Neale B	UKBB	2017
rs200293726	A	T	Plateletcrit	Astle W	27863252	2016
rs200448	C	T	Heel bone mineral density	Neale B	UKBB	2017
rs2056726	A	G	Platelet distribution width	Astle W	27863252	2016
rs2277339	G	T	Arm fat-free mass left	Neale B	UKBB	2017
rs2277339	G	T	Arm fat-free mass right	Neale B	UKBB	2017
rs2277339	G	T	Arm predicted mass left	Neale B	UKBB	2017
rs2277339	G	T	Arm predicted mass right	Neale B	UKBB	2017
rs2277339	G	T	Basal metabolic rate	Neale B	UKBB	2017
rs2277339	G	T	Comparative height size at age 10	Neale B	UKBB	2017
rs2277339	G	T	Had menopause	Neale B	UKBB	2017
rs2277339	G	T	Height	GIANT	28146470	2017
rs2277339	G	T	Height	GIANT	28146470	2017
rs2277339	G	T	Height	Neale B	UKBB	2017
rs2277339	G	T	Impedance of arm left	Neale B	UKBB	2017
rs2277339	G	T	Impedance of whole body	Neale B	UKBB	2017
rs2277339	G	T	Leg fat-free mass left	Neale B	UKBB	2017
rs2277339	G	T	Leg fat-free mass right	Neale B	UKBB	2017
rs2277339	G	T	Leg predicted mass left	Neale B	UKBB	2017
rs2277339	G	T	Leg predicted mass right	Neale B	UKBB	2017

rs2277339	G	T	Mean corpuscular volume	Astle W	27863252	2016
rs2277339	G	T	Mean corpuscular volume	Astle W	27863252	2016
rs2277339	G	T	Plateletcrit	Astle W	27863252	2016
rs2277339	G	T	Plateletcrit	Astle W	27863252	2016
rs2277339	G	T	Trunk fat-free mass	Neale B	UKBB	2017
rs2277339	G	T	Trunk predicted mass	Neale B	UKBB	2017
rs2277339	G	T	Whole body fat-free mass	Neale B	UKBB	2017
rs2277339	G	T	Whole body water mass	Neale B	UKBB	2017
rs28416520	A	G	Had menopause	Neale B	UKBB	2017
rs2844466	T	C	Arm fat-free mass left	Neale B	UKBB	2017
rs2844466	T	C	Arm fat-free mass right	Neale B	UKBB	2017
rs2844466	T	C	Arm predicted mass left	Neale B	UKBB	2017
rs2844466	T	C	Arm predicted mass right	Neale B	UKBB	2017
rs2844466	T	C	Basal metabolic rate	Neale B	UKBB	2017
rs2844466	T	C	Comparative body size at age 10	Neale B	UKBB	2017
rs2844466	T	C	Comparative height size at age 10	Neale B	UKBB	2017
rs2844466	T	C	Forced expiratory volume in 1-second, predicted percentage	Neale B	UKBB	2017
rs2844466	T	C	Granulocyte count	Astle W	27863252	2016
rs2844466	T	C	Height	Neale B	UKBB	2017
rs2844466	T	C	IgA deficiency	Bronson P	27723758	2016
rs2844466	T	C	Illnesses of siblings: diabetes	Neale B	UKBB	2017
rs2844466	T	C	Insulin-dependent diabetes mellitus	Neale B	UKBB	2017
rs2844466	T	C	Intestinal malabsorption	Neale B	UKBB	2017
rs2844466	T	C	Leg fat-free mass left	Neale B	UKBB	2017
rs2844466	T	C	Leg fat-free mass right	Neale B	UKBB	2017
rs2844466	T	C	Leg predicted mass left	Neale B	UKBB	2017
rs2844466	T	C	Leg predicted mass right	Neale B	UKBB	2017
rs2844466	T	C	Lymphocyte count	Astle W	27863252	2016
rs2844466	T	C	Medication for cholesterol, blood pressure or diabetes: insulin	Neale B	UKBB	2017
rs2844466	T	C	Medication for pain relief, constipation, heartburn: none of the above	Neale B	UKBB	2017
rs2844466	T	C	Medication for pain relief, constipation, heartburn: paracetamol	Neale B	UKBB	2017
rs2844466	T	C	Monocyte count	Astle W	27863252	2016
rs2844466	T	C	Myeloid white cell count	Astle W	27863252	2016
rs2844466	T	C	Nervous feelings	Neale B	UKBB	2017
rs2844466	T	C	Neutrophil count	Astle W	27863252	2016
rs2844466	T	C	Primary sclerosing cholangitis	Ji S	27992413	2017
rs2844466	T	C	Red cell distribution width	Astle W	27863252	2016
rs2844466	T	C	Reticulocyte count	Astle W	27863252	2016
rs2844466	T	C	Schizophrenia	PGC	25056061	2014
rs2844466	T	C	Self-reported hyperthyroidism or thyrotoxicosis	Neale B	UKBB	2017

rs2844466	T	C	Self-reported hypothyroidism or myxoedema	Neale B	UKBB	2017
rs2844466	T	C	Self-reported malabsorption or coeliac disease	Neale B	UKBB	2017
rs2844466	T	C	Self-reported type 1 diabetes	Neale B	UKBB	2017
rs2844466	T	C	Sitting height	Neale B	UKBB	2017
rs2844466	T	C	Started insulin within one year diagnosis of diabetes	Neale B	UKBB	2017
rs2844466	T	C	Sum basophil neutrophil counts	Astle W	27863252	2016
rs2844466	T	C	Sum neutrophil eosinophil counts	Astle W	27863252	2016
rs2844466	T	C	Treatment with insulin	Neale B	UKBB	2017
rs2844466	T	C	Treatment with insulin product	Neale B	UKBB	2017
rs2844466	T	C	Treatment with levothyroxine sodium	Neale B	UKBB	2017
rs2844466	T	C	Trunk fat-free mass	Neale B	UKBB	2017
rs2844466	T	C	Trunk predicted mass	Neale B	UKBB	2017
rs2844466	T	C	Weight	Neale B	UKBB	2017
rs2844466	T	C	White blood cell count	Astle W	27863252	2016
rs2844466	T	C	Whole body fat-free mass	Neale B	UKBB	2017
rs2844466	T	C	Whole body water mass	Neale B	UKBB	2017
rs35067339	A	T	Lymphocyte count	Astle W	27863252	2016
rs35067339	A	T	Lymphocyte percentage of white cells	Astle W	27863252	2016
rs35067339	A	T	Neutrophil percentage of white cells	Astle W	27863252	2016
rs35067339	A	T	Platelet count	Astle W	27863252	2016
rs35067339	A	T	Plateletcrit	Astle W	27863252	2016
rs35067339	A	T	Years of educational attainment	SSGAC	27225129	2016
rs35067339	A	T	Years of educational attainment in females	SSGAC	27225129	2016
rs353478	C	T	Had menopause	Neale B	UKBB	2017
rs3750243	C	G	Had menopause	Neale B	UKBB	2017
rs3796624	G	C	Arm fat-free mass left	Neale B	UKBB	2017
rs3796624	G	C	Arm fat-free mass right	Neale B	UKBB	2017
rs3796624	G	C	Arm predicted mass left	Neale B	UKBB	2017
rs3796624	G	C	Arm predicted mass right	Neale B	UKBB	2017
rs3796624	G	C	Basal metabolic rate	Neale B	UKBB	2017
rs3796624	G	C	Height	Neale B	UKBB	2017
rs3796624	G	C	Leg fat-free mass left	Neale B	UKBB	2017
rs3796624	G	C	Leg fat-free mass right	Neale B	UKBB	2017
rs3796624	G	C	Leg predicted mass left	Neale B	UKBB	2017
rs3796624	G	C	Leg predicted mass right	Neale B	UKBB	2017
rs3796624	G	C	Trunk fat-free mass	Neale B	UKBB	2017
rs3796624	G	C	Trunk predicted mass	Neale B	UKBB	2017
rs3796624	G	C	Whole body fat-free mass	Neale B	UKBB	2017
rs3796624	G	C	Whole body water mass	Neale B	UKBB	2017
rs4668354	C	G	Comparative height size at age 10	Neale B	UKBB	2017
rs4668354	C	G	Height	Neale B	UKBB	2017

rs4668354	C	G	Sitting height	Neale B	UKBB	2017
rs4716056	A	G	Hemoglobin concentration	Astle W	27863252	2016
rs4716056	A	G	High light scatter percentage of red cells	Astle W	27863252	2016
rs4716056	A	G	High light scatter reticulocyte count	Astle W	27863252	2016
rs4716056	A	G	Mean corpuscular hemoglobin	Astle W	27863252	2016
rs4716056	A	G	Mean corpuscular hemoglobin concentration	Astle W	27863252	2016
rs4716056	A	G	Mean corpuscular hemoglobin concentration	van der Harst P	23222517	2012
rs4716056	A	G	Mean corpuscular volume	Astle W	27863252	2016
rs4716056	A	G	Reticulocyte fraction of red cells	Astle W	27863252	2016
rs5030755	A	G	Mean corpuscular hemoglobin	Astle W	27863252	2016
rs5030755	A	G	Mean corpuscular volume	Astle W	27863252	2016
rs5030755	A	G	Mean corpuscular volume	Astle W	27863252	2016
rs55873183	A	G	Testicular germ cell tumor	Wang Z	28604732	2017
rs606920	A	G	Height	Neale B	UKBB	2017
rs62156756	A	G	Hair or balding pattern: pattern 4	Neale B	UKBB	2017
rs62244773	A	T	Hair or balding pattern: pattern 4	Neale B	UKBB	2017
rs62356073	A	G	Trunk fat-free mass	Neale B	UKBB	2017
rs62356073	A	G	Trunk predicted mass	Neale B	UKBB	2017
rs62356073	A	G	Whole body fat-free mass	Neale B	UKBB	2017
rs62356073	A	G	Whole body water mass	Neale B	UKBB	2017
rs6430545	A	T	Forced vital capacity	Neale B	UKBB	2017
rs6430545	A	T	Hand grip strength left	Neale B	UKBB	2017
rs6430545	A	T	Hand grip strength right	Neale B	UKBB	2017
rs6430545	A	T	Trunk fat percentage	Neale B	UKBB	2017
rs6569648	C	T	Arm fat mass left	Neale B	UKBB	2017
rs6569648	C	T	Arm fat mass right	Neale B	UKBB	2017
rs6569648	C	T	Arm fat-free mass left	Neale B	UKBB	2017
rs6569648	C	T	Arm fat-free mass right	Neale B	UKBB	2017
rs6569648	C	T	Arm predicted mass left	Neale B	UKBB	2017
rs6569648	C	T	Arm predicted mass right	Neale B	UKBB	2017
rs6569648	C	T	Basal metabolic rate	Neale B	UKBB	2017
rs6569648	C	T	Body mass index	GIANT	29273807	2018
rs6569648	C	T	Breast cancer	Milne RL	29058716	2017
rs6569648	C	T	Breast cancer	Michailidou K	29059683	2017
rs6569648	C	T	Breast cancer estrogen receptor negative	Milne RL	29058716	2017
rs6569648	C	T	Comparative height size at age 10	Neale B	UKBB	2017
rs6569648	C	T	Forced expiratory volume in 1-second	Neale B	UKBB	2017
rs6569648	C	T	Forced expiratory volume in 1-second, best measure	Neale B	UKBB	2017
rs6569648	C	T	Forced expiratory volume in 1-second, predicted	Neale B	UKBB	2017
rs6569648	C	T	Forced vital capacity	Neale B	UKBB	2017
rs6569648	C	T	Forced vital capacity, best measure	Neale B	UKBB	2017

rs6569648	C	T	Hand grip strength left	Neale B	UKBB	2017
rs6569648	C	T	Hand grip strength right	Neale B	UKBB	2017
rs6569648	C	T	Heel bone mineral density	Neale B	UKBB	2017
rs6569648	C	T	Height	GIANT	20881960	2010
rs6569648	C	T	Height	GIANT	23754948	2013
rs6569648	C	T	Height	GIANT	25282103	2014
rs6569648	C	T	Height	GIANT	28146470	2017
rs6569648	C	T	Height	GIANT	28146470	2017
rs6569648	C	T	Height	GIANT	20881960	2010
rs6569648	C	T	Height	Soler Artigas M	21946350	2011
rs6569648	C	T	Height	Fatemifar G	23704328	2013
rs6569648	C	T	Height	GIANT	20881960	2010
rs6569648	C	T	Height	Neale B	UKBB	2017
rs6569648	C	T	Height in males	GIANT	23754948	2013
rs6569648	C	T	Hip circumference	GIANT	25673412	2015
rs6569648	C	T	Hip circumference	GIANT	25673412	2015
rs6569648	C	T	Hip circumference	Neale B	UKBB	2017
rs6569648	C	T	Hip circumference adjusted for BMI	GIANT	25673412	2015
rs6569648	C	T	Hip circumference in males	GIANT	25673412	2015
rs6569648	C	T	Impedance of arm left	Neale B	UKBB	2017
rs6569648	C	T	Impedance of arm right	Neale B	UKBB	2017
rs6569648	C	T	Impedance of whole body	Neale B	UKBB	2017
rs6569648	C	T	Leg fat mass left	Neale B	UKBB	2017
rs6569648	C	T	Leg fat mass right	Neale B	UKBB	2017
rs6569648	C	T	Leg fat-free mass left	Neale B	UKBB	2017
rs6569648	C	T	Leg fat-free mass right	Neale B	UKBB	2017
rs6569648	C	T	Leg predicted mass left	Neale B	UKBB	2017
rs6569648	C	T	Leg predicted mass right	Neale B	UKBB	2017
rs6569648	C	T	Lymphocyte count	Astle W	27863252	2016
rs6569648	C	T	Lymphocyte counts	Astle W	27863252	2016
rs6569648	C	T	Maternal effects on offspring birthweight	EGGC	29309628	2018
rs6569648	C	T	Sitting height	Neale B	UKBB	2017
rs6569648	C	T	Trunk fat mass	Neale B	UKBB	2017
rs6569648	C	T	Trunk fat-free mass	Neale B	UKBB	2017
rs6569648	C	T	Trunk predicted mass	Neale B	UKBB	2017
rs6569648	C	T	Weight	GIANT	23754948	2013
rs6569648	C	T	Weight	Neale B	UKBB	2017
rs6569648	C	T	Whole body fat mass	Neale B	UKBB	2017
rs6569648	C	T	Whole body fat-free mass	Neale B	UKBB	2017
rs6569648	C	T	Whole body water mass	Neale B	UKBB	2017
rs6578283	A	G	Impedance of leg left	Neale B	UKBB	2017
rs6578283	A	G	Impedance of leg right	Neale B	UKBB	2017

rs6578283	A	G	Impedance of whole body	Neale B	UKBB	2017
rs6793835	A	G	Body mass index	Neale B	UKBB	2017
rs6793835	A	G	Comparative height size at age 10	Neale B	UKBB	2017
rs6793835	A	G	Height	GIANT	23754948	2013
rs6793835	A	G	Height	GIANT	25282103	2014
rs6793835	A	G	Height	Neale B	UKBB	2017
rs6793835	A	G	Impedance of arm left	Neale B	UKBB	2017
rs6793835	A	G	Impedance of arm right	Neale B	UKBB	2017
rs6793835	A	G	Impedance of whole body	Neale B	UKBB	2017
rs6793835	A	G	Worrier or anxious feelings	Neale B	UKBB	2017
rs6930435	A	G	Asthma	Neale B	UKBB	2017
rs6930435	A	G	Basophil count	Astle W	27863252	2016
rs6930435	A	G	Eosinophil count	Astle W	27863252	2016
rs6930435	A	G	Forced expiratory volume in 1-second	Neale B	UKBB	2017
rs6930435	A	G	Forced expiratory volume in 1-second, best measure	Neale B	UKBB	2017
rs6930435	A	G	Forced expiratory volume in 1-second, predicted percentage	Neale B	UKBB	2017
rs6930435	A	G	Granulocyte count	Astle W	27863252	2016
rs6930435	A	G	Heel bone mineral density	Neale B	UKBB	2017
rs6930435	A	G	IgA deficiency	Bronson P	27723758	2016
rs6930435	A	G	IgA deficiency	Bronson P	27723758	2016
rs6930435	A	G	Intestinal malabsorption	Neale B	UKBB	2017
rs6930435	A	G	Lymphocyte count	Astle W	27863252	2016
rs6930435	A	G	Monocyte count	Astle W	27863252	2016
rs6930435	A	G	Myeloid white cell count	Astle W	27863252	2016
rs6930435	A	G	Neutrophil count	Astle W	27863252	2016
rs6930435	A	G	Peak expiratory flow	Neale B	UKBB	2017
rs6930435	A	G	Potassium in urine	Neale B	UKBB	2017
rs6930435	A	G	Primary sclerosing cholangitis	Ji S	27992413	2017
rs6930435	A	G	Schizophrenia	PGC	25056061	2014
rs6930435	A	G	Self-reported asthma	Neale B	UKBB	2017
rs6930435	A	G	Self-reported hyperthyroidism or thyrotoxicosis	Neale B	UKBB	2017
rs6930435	A	G	Self-reported malabsorption or coeliac disease	Neale B	UKBB	2017
rs6930435	A	G	Self-reported psoriasis	Neale B	UKBB	2017
rs6930435	A	G	Sum basophil neutrophil counts	Astle W	27863252	2016
rs6930435	A	G	Sum eosinophil basophil counts	Astle W	27863252	2016
rs6930435	A	G	Sum neutrophil eosinophil counts	Astle W	27863252	2016
rs6930435	A	G	White blood cell count	Astle W	27863252	2016
rs7091889	A	G	High light scatter percentage of red cells	Astle W	27863252	2016
rs7091889	A	G	High light scatter reticulocyte count	Astle W	27863252	2016
rs7091889	A	G	Past tobacco smoking	Neale B	UKBB	2017
rs7091889	A	G	Reticulocyte count	Astle W	27863252	2016

rs7091889	A	G	Reticulocyte fraction of red cells	Astle W	27863252	2016
rs72934556	G	T	Chronic ischaemic heart disease	Neale B	UKBB	2017
rs72934556	G	T	Coronary artery disease	CARDIoGRAMplusC4D	26343387	2015
rs72934556	G	T	Coronary artery disease	Nelson CP	28714975	2017
rs72934556	G	T	Coronary artery disease	van der Harst P	29212778	2018
rs72934556	G	T	Coronary artery disease	van der Harst P	29212778	2018
rs72934556	G	T	Myocardial infarction	CARDIoGRAMplusC4D	26343387	2015
rs72934556	G	T	Sitting height	Neale B	UKBB	2017
rs7414807	A	G	Hematocrit	Astle W	27863252	2016
rs7414807	A	G	Hemoglobin concentration	Astle W	27863252	2016
rs746748	C	T	Pulse rate	Neale B	UKBB	2017
rs75770066	A	G	Had menopause	Neale B	UKBB	2017
rs76928871	A	G	Age at menarche	Neale B	UKBB	2017
rs780088	C	T	Alcohol intake frequency	Neale B	UKBB	2017
rs780088	C	T	Granulocyte percentage of myeloid white cells	Astle W	27863252	2016
rs780088	C	T	Had menopause	Neale B	UKBB	2017
rs780088	C	T	Height	Neale B	UKBB	2017
rs780088	C	T	Monocyte percentage of white cells	Astle W	27863252	2016
rs780088	C	T	Platelet count	Astle W	27863252	2016
rs780088	C	T	Plateletcrit	Astle W	27863252	2016
rs780088	C	T	Self-reported gout	Neale B	UKBB	2017
rs780088	C	T	Self-reported high cholesterol	Neale B	UKBB	2017
rs9796	T	A	Diastolic blood pressure	Neale B	UKBB	2017
rs9796	T	A	Height	GIANT	25282103	2014
rs9796	T	A	Height	Neale B	UKBB	2017
rs9968117	C	T	Height	Neale B	UKBB	2017
rs9968117	C	T	Pulse rate	Neale B	UKBB	2017
rs9968117	C	T	Sitting height	Neale B	UKBB	2017
rs9968117	C	T	Trunk fat-free mass	Neale B	UKBB	2017
rs9968117	C	T	Trunk predicted mass	Neale B	UKBB	2017
rs9968117	C	T	Whole body fat-free mass	Neale B	UKBB	2017
rs9968117	C	T	Whole body water mass	Neale B	UKBB	2017
